# Feeding design in free-living mesostigmatid chelicerae (Acari: Anactinotrichida)

**DOI:** 10.1007/s10493-021-00612-8

**Published:** 2021-04-30

**Authors:** Clive E. Bowman

**Affiliations:** Mathematical Institute, University of Oxford, Oxford, OX2 6GG UK

**Keywords:** Airoryhnchy versus klinorhynchy, Biomechanical adaptation, Carnivore ecomorphology, Functional morphological form, Heuristics, Rollplatte, Stochastic prediction

## Abstract

A model based upon mechanics is used in a re-analysis of historical acarine morphological work augmented by an extra seven zoophagous mesostigmatid species. This review shows that predatory mesostigmatids *do* have cheliceral designs with clear rational purposes. Almost invariably within an overall body size class, the switch in predatory style from a worm-like prey feeding (‘crushing/mashing’ kill) functional group to a micro-arthropod feeding (‘active prey cutting/slicing/slashing' kill) functional group is matched by: an increased cheliceral reach, a bigger chelal gape, a larger morphologically estimated chelal crunch force, and a drop in the adductive lever arm velocity ratio of the chela. Small size matters. Several uropodines (*Eviphis ostrinus*, the omnivore *Trachytes aegrota*, *Urodiaspis tecta* and, *Uropoda orbicularis*) have more elongate chelicerae (greater reach) than their chelal gape would suggest, even allowing for allometry across mesostigmatids. They may be: plesiosaur-like high-speed strikers of prey, scavenging carrion feeders (like long-necked vultures), probing/burrowing crevice feeders of cryptic nematodes, or small morsel/fragmentary food feeders. Some uropodoids have chelicerae and chelae which probably work like a construction-site mechanical excavator-digger with its small bucket. Possible hoeing/bulldozing, spore-cracking and tiny sabre-tooth cat-like striking actions are discussed for others. Subtle changes lead small mesostigmatids to be predator–scavengers (mesocarnivores) or to be predator–fungivores (hypocarnivores). Some uropodines (e.g., the worm-like prey feeder *Alliphis siculus* and, *Uropoda orbicularis*) show chelae similar in design to astigmatids and cryptostigmatids indicating possible facultative saprophagy. Scale matters—obligate predatory designs (hypercarnivory) start for mesostigmatids with chelal gape > 150 μm and cheliceral reach > 350 μm (i.e., about 500–650 μm in body size). Commonality of trophic design in these larger species with solifugids is indicated. *Veigaia* species with low chelal velocity ratio and other morphological strengthening specialisms, appear specially adapted in a concerted way for predating active soft and fast moving springtails (Collembola). *Veigaia cerva* shows a markedly bigger chelal gape than its cheliceral reach would proportionately infer suggesting it is a crocodile-like sit-and-wait or ambush predator *par excellence*. A small chelal gape, low cheliceral reach, moderate velocity ratio variant of the worm-like feeding habit design is supported for phytoseiid pollenophagy. Evidence for a resource partitioning model in the evolution of gnathosomal development is found. A comparison to crustacean claws and vertebrate mandibles is made. *Alliphis siculus* and *Rhodacarus strenzkei* are surprisingly powerful mega-cephalics for their small size. Parasitids show a canid-like trophic design. The chelicera of the nematophagous *Alliphis halleri* shows felid-like features. *Glyphtholaspis confusa* has hyaena-like cheliceral dentition. The latter species has a markedly smaller chelal gape than its cheliceral reach would suggest proportionately, which together with a high chelal velocity ratio and a high estimated chelal crunch force matches a power specialism of feeding on immobile tough fly eggs/pupae by crushing (durophagy). A consideration of gnathosomal orientation is made. Predatory specialisms appear to often match genera especially in larger mesostigmatids, which may scale quite differently. Comparison to holothyrids and opilioacarids indicates that the cheliceral chelae of the former are cutting-style and those of the latter are crushing-style. A simple validated easy-to-use ‘2:1 on’ predictive algorithm of feeding habit type is included based on a strength-speed tradeoff in chelal velocity ratio for ecologists to test in the field.

## Introduction

Many mesostigmatid mites are free-living predators (Zakhvatkin 1952; Krantz and Walter 2009), whether residing: edaphically/hemi-edaphically in forest litter, eudaphically in soil (Kühnelt 1961; Walter and Proctor 2013), on plants (Evans et al. 1961), or in temporary accumulations like nests, dung, and carcasses. On the forest floor, their large size and enormous gluttony make up for their only modest density in their importance to the community (Van der Drift 1950).

Their anterior feeding structure comprises the gnathosoma and palps. The terminology used for some of the gnathosomal structures has been subject to instability (Kazemi 2020). The mesostigmatid gnathosoma (confusingly sometimes referred to as a capitulum; Gorirossi and Wharton 1953) is dominated by a symmetrical pair of retractile three-article jointed chelicerae each usually furnished with a grasping or cutting chela distally (Gorirossi 1955c; Alberti and Coons 1999). Each tubular, elliptical in cross-section, cheliceral shaft is typically two-segmented with a proximal (to the opisthosoma) basal segment (I) and a distal segment (middle article-II). Other variants occur (Evans 1992; Alberti and Coons 1999). Using mechanisms common for leg movement in arachnids (Manton 1958b), muscles attached within the opisthosoma retract the chelicerae—intra-idiosomal hydrostatic pressure extends them. The chelicerae are capable of retraction and protraction independently or in unison (Evans 1979; Alberti and Coons 1999). Cheliceral retraction draws grabbed prey towards the hypostome often impaling it upon gnathotectal processes and corniculi (Zukowski 1964). For instance, the long lanceolate ventrally curved gnathotectum of eviphidids may assist in the holding or perhaps the piercing of their nematode prey. Salivary enzymes (Bowman 2019) delivered by stylets (Evans 1992; Krantz and Walter 2009) facilitate prey liquefaction. The gnathotectum above the chelicerae together with the palps (Bowman 1984), plus the tritosternum and gnathosomal groove (Wernz and Krantz 1976) can maintain a fluid cylinder extended around the hypostome and mouth (Alberti and Coons 1999).

**Fig. 1:**
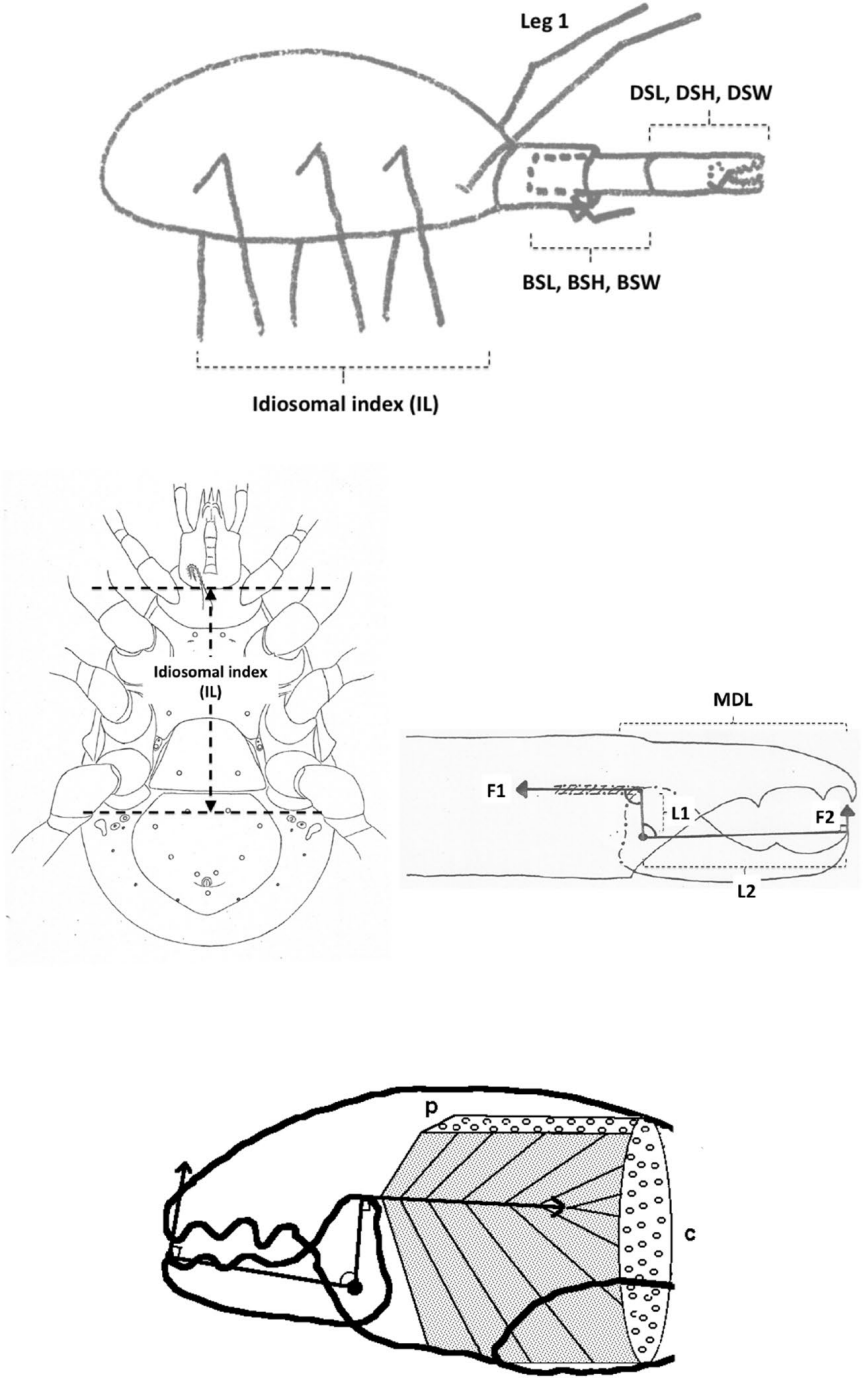
Morphology and cheliceral physics in mesostigmatids. Diagram of measurements made (*IL* idiosomal index Lynch 1989, *BSL* basal cheliceral segment length, *DSL* distal cheliceral segment length, *MDL* moveable digit length, *HDS* height of distal cheliceral segment, *WDS* width of cheliceral distal segment, *HBS* height of basal cheliceral segment, *WBS* width of basal cheliceral segment, *L1* adductive ‘input’ lever arm of moveable digit, *L2* adductive ‘output’ lever arm of moveable digit); and, derived adductive force measurements (F1, F2). *Upper* Stylised lateral view of whole mesostigmatid mite. Cheliceral segment heights, widths and lengths taken where they were orthogonally at their maximum. Cheliceral length CL = BSL + DSL. Pedipalps drawn under gnathosoma and second chelicera omitted for clarity. *Middle* *Left* Idiosomal index (IL in μm) measured orthogonally and ventrally from circum-capitular groove (gnathosomal socket) in front of coxae I to line touching rear of coxae IV. Annotated from a personal drawing of Don E Johnston. *Right* Stylised mesostigmatid cheliceral chela as a Class 1 lever system. Dot is position of articulating dicondyle. Levator tendon shaded. All angles assumed to be 90 °C for simplicity (see Materials and methods section). Moveable digit length (MDL) is from the extremum posterior of condyle to tip of moveable digit. Moveable digit depressor tendon ommitted. *Note* MDL $> \text {L}2$. Annotated from a personal drawing of Don E Johnston. *Lower* Generic mite cheliceral chela showing two assumptions regarding levator muscle force F1. *p* pennate. *c* circular

The mouthparts of arthropods are very hard (Bailey 1954)—just scratching calcite with difficulty (= three on Moh’s scale). A variety of differently sclerotised cheliceral chelae is found in mesostigmatids (Hirschmann 1959). Their darkness is interpreted as evidence of increased strengthening. In plesiomorphic form, the chela comprises an opposable dentate moveable digit (article III—sometimes confusingly labeled as an ’apotele’) whose acetabulae rotate in a chitinous dicondyle (Woodring and Galbraith 1976; Krantz and Walter 2009), or pivoting articulation (Manton 1958b) arising from the internal faces of article II. This ginglymic ‘jaw’ closes vertically (mygalomorph-like; Bristowe 1954) against an often toothed fixed digit extension of the distal segment of the chelicera (Grandjean 1947; Krantz and Walter 2009). The rotation axis and fulcrum of this class 1 lever assembly is at these two condylar articulation points (Fig. 1)—for a definition of lever classes see Davidovits (2018). The tip of one or the other digit can be hooked (like the beaks of birds of prey where it is used to tear and pull at the prey rather than killing it; Brown and Amadon 1968). Muscles (taxonomically highly conserved across the Chelicerata; Snodgrass 1948) within the cheliceral shaft connected by strong tendons in article I and II can close and (unlike scorpion pedipalp chelae; Matthew 1965) open each chela independently. In essence the whole assembly within the gnathosoma acts like a protrusible fish jaw (with the ability for the mesostigmatid left and right side to chew independently). This has significant advantages for dealing with evasive or substrate-dwelling prey (Hulsey et al. 2010).

### Diversity of form and function

Traditionally the Mesostigmata are divided into gamasid and uropodoid forms, the latter being generally feeders on fungal mycelium, spores and decaying plant material with weakly developed chelicerae (Hughes 1959). Most species of the suborder Uropodina show bizarre morphological specialisations that obscure their underlying taxonomic relationships (Babaeian et al. 2018). Although usually predaceous, some gamasid species graze on fungi, others ingest fungal spores and hyphae (Walter and Proctor 2013). Despite some being exclusive nematophages (Evans 1992), most species of eviphidids are coprophilous (Mašán and Halliday 2010) with 50% of European genera being detriticoles. Some uropodine mites feed on plant sap (Forsslund 1943). Many gamasines readily feed on nematode prey (Imbriani and Mankau 1983; Walter 1987; Krantz and Walter 2009; Stirling et al. 2017; Manwaring et al. 2020) their actions prejudicing the use of nematodes as biocontrol agents (Epsky et al. 1988). In all grasping forms, chelate chelicerae are used in feeding upon small prey or other food stuffs and port material into the labral area for potential straining (Flechtmann et al. 1994) and uptake via the pre-oral groove (Evans and Loots 1975). Ingestion of resultant prey fluids is the norm (Evans 1992; Alberti and Coons 1999). Diversity of structure between species and between active stases is found (Evans 1992; Alberti and Coons 1999). Some digits are tong-like. Other chelae are serrate having a row of small, closely set teeth resembling a saw. Snapping forms often have a few widely spaced teeth. Some mesostigmatid species lack or have a reduced fixed digit extension, relying upon impaling their prey with a sharp moveable digit (often edentate with no teeth—Evans et al. 1961). Examples of species with highly elongate moveable digits are *Blattisocius tarsalis* (Berlese) which stabs insect eggs and drains them, or the moth-associated Otopheidomenidae (Treat 1975). Some species may have longer fixed digits than moveable digits (Adar et al. 2012) commensurate with stabbing surfaces (like leaves) or prey. In contrast, parasitic forms have highly derived gnathosomas (Lagutenko 1967; Radovsky 1968) and are not considered herein.

Every species is specialised in some way to some extent (Futuyma 1979). A central issue in evolutionary biology concerns whether morphology, performance and habitat use have coevolved. Evolutionary morphologists seek patterns in scaling relationships and form-function correlations to gain insights into the biomechanical or adaptive significance of differences in morphology among species (Radinksy 1985). Carnivorous vertebrates show a remarkable history of adaptive radiation characterised by the repeated independent evolution of similar morphologies in distinct clades (Van Valkenburgh 2007). For many mites, especially the Mesostigmata, nematodes are a preferred prey (see references in Epsky et al. 1988; Evans 1992; Walter and Proctor 2013). Are there clear functional groups in mesostigmatids?^1^ Ecological studies (e.g., Walter et al. 1988) systematically list prey types for different mesostigmatid species. Typically (e.g., Sadar and Murphy 1987), apart from any extreme morphological forms, these studies list most species as polyphagous, or result in inconclusive assignations to trophic groups. There are also many historical anecdotal records of specific free-living mesostigmatid feeding; e.g., Karg (1962) describing *Pergamasus misellus* (Berlese) as an ‘oligophagous gamaside’ because it fed on collembola and nematodes, or Costa (1964) describing *Parasitus copridis* Costa using its chelicerae to mash to a pulp dung-living nematodes three to four times its own size. Kinn (1971) records *Eugamasus* spp. feeding on nematodes and mites in bark beetle galleries. Kühnelt (1950) suggested that many of the larger predatory mesostigmatids may be also facultatively coprophagous or necrophagous. Evans (1992) states that *Uroobovella marginata* (Koch), *Uroobovella rackei* (Oudemans), *Uropoda orbicularis* (Müller) and *Uropoda sellnicki* Hirschmann and Zirngiebl-Nicol prefer nematodes as food. Porcelli et al. (2009) associates *Centrouropoda almerodai* and *Uroobovella marginata* feeding with the red palm weevil in palm tissue borings. *Gamasellus racovitzai* (Trouessart) preys upon larvae and nymphs of the oribatid *Alaskozetes* but not its adults (Block 1984)—yet also eats antarctic nematodes. Some uropodines like *Protodinychus* feed on animal material both living, injured and dead (Kühnelt 1950) but most sedentary uropodids tend to be mycetophagous (like *Fuscuropoda marginata* (Koch); Evans et al. 1961) or coprophagous. Manure-inhabiting species will eat nematodes (Ito 1971). The myrmecophilous uropodine *Oplitis* is reported to suck on the excrement of its host (Constantinescu 2012).

### Are there adaptive designs?

Notwithstanding this diversity, could the design of mite mouthparts *actually* reflect specific adaptations for predation? Given that phylogenetic relationship is an indication of shared constraints on behaviour, physiology and morphology (Walter and Ikonen 1989)—where in the classificatory hierarchy is there commonality in these trophic abilities?

Gamasids show a remarkable range of forms, many of which feed on eggs, pupae and larvae of insects, small worms, other mites, and collembola (Hughes 1959), but cannibalism is rare (Walter and Proctor 2013). However, there are a limited number of ways to ecologically partition the carnivore niche given the material properties of animal tissues (Van Valkenburgh 2007). Karg (1983) suggested that families and genera of mesostigmatid mites have become specialist feeders with those specialisms instantiated in their mouthpart structure. Hirschmann (1956) suggested a dual function for their chelicerae: ‘Greiffunktion’ (gripping) and ‘Kaufunktion’ (chewing). Hirschmann (1959) noted a trend for longer and narrower chelicerae in mesostigmatid carnivores.

Following in the tradition of Hespenheide (1973), Buryn and Brandl (1992) undertook a comparative morphology study of mesostigmatids more than 25 years ago using principal components analysis (PCA) attempting to relate various cheliceral measurements to feeding specialisms. This review revisits this and extends it. Could distinctive cheliceral forms provide an indication of how their owners made a living?

Implicit in Buryn and Brandl (1992)’s approach was that in some space—*at any scale*—‘two different designs fit all’ of the predatory mesostigmatids in general (with a third design—polyphagous, and a fourth design—omnivore, as some sort of compromises between the two for exceptional species who might feed upon various proportions of animal and plant tissues). Buryn and Brandl (1992) found only weak associations of relative cheliceral size and absolute dentition levels with the ability to handle either worm-like or arthropod prey. In retrospect, this is rather surprising given the earlier success of relating chelal form to function in cryptostigmatids (Schuster 1956; Kaneko 1988; Perdomo et al. 2012) and astigmatids (Akimov and Gaichenko 1976). Moreover, later work (Adar et al. 2012; Liu et al. 2017) focusing on a narrow set of phytoseiid plant–pest predators has suggested that some morphometrics of mainly the chelicerae of their gnathosoma *are* related to their feeding habits.

Although Walter and Ikonen (1989) concludes that feeding behaviours in grassland soil mesostigmatids are rarely predictable above the generic level, given that most other animals’ bodies suit their life-style and home (Manton 1958a), and that certainly vertebrate animal jaws and teeth show adaptations for food or morsel processing, it is worth trying to extend and augment Buryn and Brandl (1992)’s approach to see if stronger further insights can be gained. Furthermore to validate these new insights using the data from Adar et al. (2012) and Liu et al. (2017) for phytoseiids whose cheliceral morphologies are historically hard to correlate with feeding types a priori (McMurtry 1982). An extra objective is also to seek to better understand the trophic design for phytoseiid pollenophagy—a common habit in that group, where they digest the exine externally and ingest resultant fluids (Walter and Proctor 2013). An interim comparison will also be made to primitive anactinotrichids. This is the general aim of this hypothesis-testing paper. Additionally, although direct feeding observations are not made, any consilience with possible style of predation will be pointed out (‘cruising/pursuit predators’ versus ‘ambush predators’—Walter and Proctor 2013).

## Rationale for approach

An ‘equilibrium analysis’ is when organisms are considered to be in equilibrium with the environment and present-day environmental correlates of structure are sought in an attempt to explain morphology (Lauder 1982). Morphologists study structure and function so as to produce insights for field ecologists to interpret species that they may find which have not been well studied, for example, in birds (Grant 1986), cichlid fish (references in Bouton et al. 2002), or lizards (references in Bickel and Losos 2002). Hopefully a predictive algorithm of food preferences from morphology can be built for field use. It is important to best phrase the functional groups expected in terms that make practical sense.

So, three topics should be considered in such ecomorphological studies together with their corrolaries:

(i) The span of species used;
(ii) The analytical space over which comparisons are made or ordinations prepared; and,
(iii) The physics (Gans 1974) and expected results of any tacit mechanical model employed to explain the biology involved.

Taking each of these in turn.

### The span of species used

The conclusions one can make regarding a phenomenon depend crucially upon the ascertainment ($\equiv$ collection process) of the actual sample over which one wishes to make any inference (point (i) above). Buryn and Brandl (1992) did well in choosing a large number of mesostigmatid species (52 within 29 genera) over a wide range of idiosomal sizes (smallest 299.3 μm = *Rhodacarellus sileciacus* Willmann to largest 1323.3 μm = *Pergamasus septentrionalis* (Oudemans)). Size certainly matters to mites (Proctor and Walter 2018), with some mesostigmatids being of enormous scale (Cómbita-Heredia et al. 2018, 2020). The gnathosoma of one of these has already been investigated in depth (Gorirossi 1956). However, Buryn and Brandl (1992) may have been unlucky in their attempted coverage of all chelate morphotypes. For instance relatively few uropodines and phytoseiids were examined, nor were all definite nematode or fly egg/larvae/pupae consuming specialists included. They did, however, include veigaids thought to be specialist collembola feeders (Hurlbutt 1965; *Veigaia pusilla* (Berlese) is a springtail predator; Walter and Ikonen 1989; Walter et al. 1988).

The tendency to evolve highly convergent ecomorphs is most apparent among feeding extremes where performance requirements tend to be more acute (Van Valkenburgh 2007). So, this study seeks to augment the previous species used over the same size range by adding:

- A facultatively nematophagous dung-living common mycophagous uropodine with markedly elongate chelicerae *Uropoda orbicularis* DN (Faasch 1972). Many uropodoids are regarded as consumers of dead things but if they do consume small soil animals they cannot take fast-moving collembola (Kühnelt 1961).
- A predatory uropodine *Polyaspis* n.sp. DN ex Brazil.
- A large common known predator of various fly developmental stages in dung *Glyphtholaspis confusa* (Foa) female.
- Another species of fluid feeding (Walter et al. 1988) small nematode specialists (Karg 1971) with short stubby chelicerae *Alliphis halleri* female from compost.
- Deutonymphs of more species of the common genus *Parasitus* (Hyatt 1980). Only one species of the latter genus was used by Buryn and Brandl (1992) despite their ubiquity as common phoretics of carabid and silphid beetles (Krantz 1978). Gamasid deuteronymphs do feed even when on their host (Evans et al. 1961). Three more are included to offer another within-genus series:
  - the dung-inhabiting, beetle elytra-sheltering *Parasitus coleoptratorum* DN is an enchytraeid feeder (Rapp 1959), a nematode feeder (Karg 1971), and is included as a positive control. It also attacks fly larvae (Wernz and Krantz 1976) in dung (Krantz 1978).
  - the *Bombus* bumble-bee commensal *Parasitus* (now *Parasitellus*) *fucorum* DN. As reported by Karg (1971), it was thought by Vitzthum (1930) to be predatory. Although data concerning it in the literature is generally scarce (Chmielewski 1971), Vitzthum (1943) later thought it be a coprophage. Its large blunt teeth resemble those of snail-crushing lizards (Dalrymple 1979a).
  - the mite *Parasitus (Cornigamasus) lunaris* DN, known from dung (Evans et al. 1961) but collected in compost/grass cuttings.
- A new sample of *Veigaia nemorensis* (Koch) female—a massive chelicerae mesostigmatid (Evans 1955) known to feed on tyrophagids and immature Oribatei (Karg 1961) is included again for cross-study comparison. *Veigaia nemorensis* is a feeder of soft-bodied acarid, tydeid and immature cryptostigmatid mites as well as collembola (Karg 1971).

Given the extensive coverage by Adar et al. (2012), no extra phytoseiids were added. *Polyaspis* spp. are known feeders on rhabditid nematodes (Muraoka and Ishibashi 1976). Gamasid deuteronymphs do feed even when on their host (Evans et al. 1961). In all cases each taxon sample is assumed to be typical for its geography. Some consilience of the results with taxonomic position is expected as it is assumed that the evolution of the mites’ external morphology has already reached a high degree of perfection and that genetically-mediated adaptive changes in these species now proceed largely through physiological channels (Dobzhansky 1956).

### The analytical space over which comparisons are made

Ecomorphological correlates are often displayed as ordinations. In animals, their size (i.e., their scale) is very important (Calder 1983; Schmidt-Nielsen 1984)—point (ii) above. Trophically this is pertinent. For instance a large lion does not generally predate small mice (although if it caught them, it would eat them—just as large snakes have no lower limit to prey mass; Rodríguez-Robles 2002). If one wants to arrange morphological data within a rigorous scientific explanation—rather than just simply preparing an empirical ordination based upon the observed variation of that which was measured—then any analysis space used needs to have a coherent and consistent basis. In other words—it should be isotropic (up to any linear proportionality factor) in all directions with respect to a change in magnitude. This is particularly true when using an analysis method, like the orthogonal singular value decompositions (SVD) in PCA, for it then to be appropriately interpretable mechanistically. What is needed is that a unit change in any direction in the multidimensional space of the morphological measurements taken should be linearly the same wherever you are in that space. That is, it should be both independent of the type of measurement made and what general size the object being measured is. This is especially important for any across-species morphological SVD analysis with individuals which vary markedly in size—as tensors such as correlation or covariation matrices are Riemannian not Euclidean. Accordingly this study herein seeks to use a modest-span isotropic metric space whenever practical and unlike earlier authors does not use chelal dentition explicitly in its analysis. Although, Karg (1983) uses dentition as well as the stoutness of cheliceral chelae in classifying nematophagous forms, focusing on dentition before general design considerations are understood can yield confusing conclusions (e.g., *Cheiroseius* spp. being placed in both Type 2 and Type 3 designs by Walter and Ikonen 1989).

With respect to its diameter increasing, the volume of an inflating balloon grows much faster than its surface area increases. Physics intervenes for any phenomenon under a scale change. When three dimensional living species get bigger, similarly scaled versions of themselves do not stay the same shape (Rosen 1967). Consider that the design of an elephant leg (and how it is used in walking) is different from the design of a mouse leg. It would be impossible for a mouse the size of an elephant to function—its legs would collapse under its own body weight. Conversely a mouse does not walk ‘stiff-legged’ like an elephant and unlike pachyderms it can do a spring-jump easily. A size normaliser is thus needed in comparisons. The evolution of large size and carnivory may be favoured at the individual level (Van Valkenburgh 2007) but natural selection does not see ratios so dividing (or regressing) out size seems inappropriate in any primary analysis. Rather nature does see actual size on some tangible scale. So, eschewing the fine detail of measurement covariation within a species, a general purpose transformation based upon metabolic scaling arguments (Cloudsley-Thompson 1977), that up to a linear proportionality factor in orthogonal directions ensures modest scale isotropy between species—is the log transform of continuous measurements. This effectively steers consideration of natural selection mechanism towards multiplicative processes. This has been used widely in arthropod research (e.g., Huber 1985).

Using this global geometric similarity (Huxley 1924) or allometric relationship (Gould 1966, 1971) as the basis of comparative measurements means that a major size change will engender a shape change as you would expect automatically. The starkness of this perceived shape change depending upon the value of the allometric power parameter. In biology over the decades there are many examples of the consequences of such run-away growth gradients like: in spider mouthparts (Lockett 1932), in horned beetles (Arrow 1951), in the sexual selection of mandibles of different size stag beetle species (Otto and Stayman 1979), or the antlers in deer for example (Alexander 1971). Evolution affecting characters within, and further evolution between, species can then superimpose other particular local growth gradients on this base mechanism metric to differentiate particular trophic forms (for example, Leamy and Bradley 1982; Leamy and Atchley 1984; Schluter and Grant 1982; Francis and Guralnick 2010; Kaliontzopoulou et al. 2010). Mesostigmatids will be checked for any marked allometry. Although male mesostigmatids chelicerae are often used for sperm transfer (Hirschmann 1954; Alberti and Coons 1999), by focussing on females and deuteronymphs any aspects of sexual selection on chelal form need not be considered in this study. Similarly no conclusions are made as to cheliceral utility for mesostigmatid fighting (Lindquist and Walter 1989) or, in the first instance, for holding onto a host during phoretic dispersal (e.g., Błoszyk et al. 2006).

The log transformation of measurements also has the effect in multivariate analyses of making any variation more homogeneous between species and so simple statistical tests more defendable. Both linearity and multivariate normality are often more closely approximated by logarithms than the original variables (Pimentel 1978). Covariance estimates of data after log transformations approximate the independence from scale and magnitude of the original variables considered by a correlation analysis of the original measurements. Although Buryn and Brandl (1992) use this log transformation, they then go on to produce measurement residuals based upon idiosomal size regressions in their principle analysis. This shift is fine but the latter approach moves one into the space of the *relative* sizes of structures (to effectively dimensionless quantities; Radinsky 1981). Using these indices might be suitable for an abstract discussion of design form, but nature sees the physicality of trophic structures, and whether their *actual* sizes confer a selective advantage or not (Calder 1984). Adaptations to nature are often concerted too (e.g., skull changes in finch feeding; Bowman 1961). Nature should guide mathematics (Paine 1996) not vice versa. This study herein therefore uses, in the first instance, the actual sizes in any plots or any multidimensional ordination. Correlation analysis rather than covariation analysis is used so as to avoid the scales of measurement variation confounding any biological analysis.

Initial prey capture (i.e., seizing) is not explicitly considered here to necessarily be determined by cheliceral design. For instance the enchytraeid-feeding *Parasitus coleoptratorum* will happily take astigmatid mites or even its own juveniles if they cannot run away fast (Rapp 1959)—showing that a gnathosomal design for one purpose can be reused for another under certain circumstances. In fact, Usher and Bowring (1984) show that raptorial success in gamasellid mites is much better related to the length of the first leg (rather than the pedipalps or chelicerae as others have suggested), and thus by implication is dependant upon body scale. Accordingly idiosomal size is not partialed out in this study like in Buryn and Brandl (1992) but is used as a surrogate measure to classify mites into potential prey size groupings. Similarly piercing *per se* by chelicerae is not considered except in the discussion on pollen feeding.

### The physics to explain the biology involved

Finally turning to point (iii) above. Jaws have been well studied in vertebrates since Smith (1968) popularised mathematics to understand biology. Although mechanically inefficient, viewing a ‘jaw’ (like the chelal moveable digit) as a lever with a condyle as its fulcrum (Fig. 1) is a widely accepted model (Smith 1978). Statics considers bodies at rest and the forces between them at equilibrium (i.e., a strength focus around a beam model)—see Barghusen (1972). Dynamics considers forces producing a changing direction of movement (i.e., a speed focus around a swinging motion model)—see Olson (1961). Carnivores have different mechanical designs according to their trophic habit. For predators this is dependent upon their differences in killing behaviour (Radinsky 1981). Therefore a rephrasing of Buryn and Brandl (1992)’s groups is made. Herein there is a contrast (Walter and Proctor 2013) between designs that can cut, slice, tear and rip holes in the side of animals and lacerate/shred their internal tissues, versus those designs that hold, mash and chew prey bodies. This parallels the contrast of chelae found in Crustacea. It is acknowledged that, as in carnivorous vertebrates, this categorisation is not entirely discrete and that they grade into one another to some extent. However, this is useful as there are limited ways to subdivide the carnivore niche for any broad analysis (the other factors being prey size and the proportion of foodstuff types consumed; Van Valkenburgh 2007). In both cases tissue maceration i.e., food softening by soaking in an extracorporeal liquid is assumed before ingestion (Bowman 2019).

Micro-arthropod feeding is mapped to ‘slicing’, and worm-like feeding is mapped to ‘crushing’ on the grounds that except for gigantic predators, crushing of whole actively-moving food can only occur for prey substantially smaller than oneself. So, if one puts aside matters of how prey is actively searched for, or detected (say, by leg 1), or grabbed/pinned down (say, by the predator’s legs with the assistance of pedipalps; Lee 1974; Usher and Bowring 1984; Krantz and Walter 2009), a simple tacit model of mite feeding design is that the food which can be dealt with, especially for mesostigmatid predators working individually, depends upon the mites’:

1. physical size (which determines their body access to locations or pores where prey may be found and can be punctured—Walter and Ikonen (1989), or determines how big a prey that can be attacked),
2. cheliceral reach (which determines gnathosomal access to actual food material to be triturated and ingested, especially if it is mobile prey where it impacts the likely gnathosomal ‘attack radius’),
3. chelal gape, i.e., the cutting/grasping surface (which determines the maximal food morsel size), and
4. crunch or grasping force that their cheliceral chelae can deliver (which determines how tough a possible prey food material can be successfully cut or mashed, and together with gape effectively determines how quickly a jaw can snap shut).

What are the underlying assumptions being made here? The model scheme assumes that any use for facilitating grasping a partner for copulation (or for spermatophore transfer in males) is not important. The model scheme assumes that micro-arthropod cuticle cannot be easily cracked, but needs to be cut. The model scheme assumes that most ‘worm-like prey’ are soft and unarmoured. The model scheme assumes that nematode cuticle is amenable to rupture by crushing. The model scheme assumes that, all other matters being equal, micro-arthropods can move and escape faster than worm-like prey. These are all reasonable simplifications in the first instance.

Additionally, the model scheme assumes that most general predators predate other animals of an approximately similar order of body magnitude as themselves (Polidori et al. 2010). For instance *Gamasellus racovitzai*, an active-searching broad-diet generalist attacks most appropriate size invertebrates it encounters (Block 1984). Phytoseiids are thus searchers (Evans 1992) rather than pursuers.

Only specialist creatures adopt adaptations to consume prey of markedly different scales—for instance, consider large grazing whales developing oral baleen to sieve out very small krill from sea-water. Therefore, common-sense suggests that of the four factors numbered above, one would expect size (1) and crunch force (4) to be the primary determinants of a predatory mesostigmatid’s feeding habit, i.e., the capability of the system. Insect cuticle (and probably other arthropod cuticle) is an excellent material for resisting bending (Wainwright et al. 1976), so a substantial force applied is to be expected. Of course for those soil predatory mesostigmatids observed to attack a large worm prey simultaneously in numbers (see Walter and Proctor 2013), size (1) may be less important relative to crunch force (4). Muscular force typically scales with cross-sectional area—muscular action in feeding often working indirectly on food via levers.

Fundamentally an animal has a choice between a musculoskeletal system aimed at a large adductive force but with relatively little motion and speed thereof of that movement at the tip of the jaw (i.e., a strength design) or the opposite mechanical properties of a fast-striking grabbing and capturing design (Carlson and Wainwright 2010; Schenk and Wainwright 2001). So, then one would expect gape (3), and then reach (2) to be the next most important determinants. That is, only once one has body access/ability to grasp prey and the capability to rupture it in some manner, does the issues of how deep you can reach into something and how big a bite can be taken from it comes into play (i.e., the capacity of the system). After that exact dentition and their form may be important in clarifying which trophic design is best suited for an ‘active slicing’ functional group versus a ‘crushing/mashing’ one.

### Corrollaries of using a mechanical model

The pinching forces of Crustacea are remarkably comparable to the values for the closing forces of vertebrate jaws (Claussen et al. 2008), and so those of mite chelae are not expected to be fundamentally different either (given of course, their scale). Maximum closing forces vary tremendously among both crustaceans (Taylor 2000) and animals in general, with body size and food habits being among the most important determining factors. Similar should be expected of mesostigmatids. Powerful crushing forms in percids (Carlson and Wainwright 2010) are used in biting relatively slow-moving prey or prey items attached to the substrate. These designs complement other forms designed for the biting and picking of elusive prey (long reach in mites being functionally equivalent to the ‘suction onto the mouth’ competency in fish feeding).

Historically only general statements about the raptorial advantages of acarine cheliceral design have been made (e.g., Hirschmann 1956). Karg (1961) noted that nematode feeders have short chelicerae with large teeth for crushing prey. The chelicerae of mites predacious on other mites and on Collembola are known to be long, slender, and possess backward pointing teeth. Karg (1971) characterised nematode feeders further in having short, stout digits with a few often offset teeth (a type commonly found in the Eviphididae (Evans 1992). He also characterised those catching rapidly moving prey as having longer more slender digits and those polyphagous predators such as *Hypoaspis aculeifer* (Canestrini) as having strong digits with a row of small closely set teeth on the fixed digit (Karg 1971; Evans 1992).

In fact, clear ecomorphological correlates arise if physics is included in animal morphological analyses (Smith and Savage 1959; Bowman 1961). Ordination in metric spaces is directly interpretable with euclidean models such as comparing the mechanical advantage of levers deployed by animals (Alexander 1968). Static jaw systems in vertebrates with large velocity ratios at the fulcrum are associated with semi-immobile hard-to-cut food material (cf. tough herbivore skin) or hard-to-crush food material (cf. carrion bone). Those jaw systems with small velocity ratios are typified by gentle tweezer actions (cf. insectivorous beaks in birds), cutting/slicing prey (cf. crustacean chelae), or needing to quickly grasp excessively slippery material (cf. crocodiles holding fish, or shrews holding worms).

This study also assumes that the slicing habit in mesostigmatid chelae is more like the design action of the beaks of

- *Certhidea* spp. warbler finches and tools like ‘needle nose pliers’
- *Pinaroloxias* spp. Cocos Island finches and tools like ‘curved needle nose pliers’
- *Cactospiza* spp. woodpecker and mangrove finches and tools like ‘long chain nose pliers’

see Bowman (1963). Further, that the crushing habit in mesostigmatid chelae is more like the design action of the beaks of

- *Platyspiza* spp. vegetarian finches and tools like ‘parrot-head gripping pliers’
- *Geospiza* spp. ground and cactus finches and tools like ‘heavy duty linesman’s pliers’
- *Carnarhynchus* spp. tree finches and tools like ‘high leverage diagonal pliers’.

Despite the clue about aspect ratios in Table 2 of Buryn and Brandl (1992), that their second principal component (PC2) differentially weights the (size-adjusted) width of chelal measures against their (size-adjusted) lengths, they did not use a mechanical model. This study deploys one and, so far as practical, re-analyses their data, testing it against phytophagous mite-consuming phytoseiids (Adar et al. 2012; Liu et al. 2017). Differences in bill musculature is correlated with differences in adductive power in finch feeding (Bowman 1961)—so explicitly including an estimated crushing force allows adding conclusions around soft-body mite/collembola prey feeding specialisms in mesostigmatids that Buryn and Brandl (1992) could not make easily (and also to make omnivory easy to explain). Retaining body scale (not partialing it out) allows contrasts to be made between mites within the same size classes, as well as examining overall scale effects within a predation-style functional group.

Retaining a simple clear distinction between functional groups with predictive value is essential in order for ecologists to use them (Walter and Ikonen 1989). Accordingly this review looks simply at the design contrast “worm-like prey” versus “micro-arthropod prey” feeding and attempts to build a useful deployable stochastic predictor.

## Expected results for free-living mesostigmatids

As in carnivorous vertebrates, a limited array of ecomorphologies is expected (Van Valkenburgh 2007). However, it is not immediately clear why departures from expected relative reach or expected relative gape values (i.e., the factors arising from Buryn and Brandl 1992’s regressions) should be biologically important in determining predator performance. Rather, it is posed herein that whether a mesostigmatid focuses on feeding upon worm-like prey (‘crushing/mashing’) or micro-arthropods (’active cutting/slicing’) is:

(i) unrelated to overall size, as
  worm-like prey or arthropods could be of any size, and
  environmental pores that a mite accesses could be of any size and, although the prey must also be able to access the same location, pore size is not *intrinsically* related to food type (except at minute scales);
(ii) not related to cheliceral reach, unless
  feeding in deep crevices within a pore size is related to *predatory* food type, which is unlikely, or
  one type of prey has a changing nutritive composition with scale compared to another (e.g., perhaps micro-arthropod highly nutritionally rich “goodies” are found deeper in their body than those in worm-like prey?);
(iii) may be related to chelal gape, in that
  collembola/springtail feeding specialists ought to have crocodile-like jaws enabling snapping shut quickly, and
  saprophagous omnivores ought to have a small gape for feeding on fungal, plant, dead tissue etc fragments—even if they have have specially adapted chelate chelicerae for squeezing the liquid contents out of mycelial masses and into their prebuccal cavity (Walter and Lindquist 1989) rather than ingesting solids;
(iv) related to the velocity ratio of the chela, as
  related arthropods like Crustacea carry distinct ‘cutting’ chelae and ‘crushing’ chelae—Hughes (2000);
(v) clearly related to the chelal crunch force, as
  size-for-size micro-arthropods are more armoured in general than worm-like prey such as nematodes and enchytraeids, and
  scorpion species are already characterised as with strong pincers or with weak pincers (Meijden et al. 2010, 2012a).

These hypotheses are labeled (i)–(v) for easy cross-reference in the Results section. It is expected that there will be found more evidence for (v) than for (iv) than for (iii)—with little evidence found for (i) or for (ii).

## Materials and methods

*Parasitus fucorum* (De Geer) was collected from *Bombus lapidarius* (L) in the UK. *Parasitus lunaris* (Berlese), *Polyaspis* n.sp. and *Veigaia nemorensis* were taken from the Ohio State University, Acarology Laboratory spirit collection. Almost certainly, the polyaspid was that later described by Hirschmann and Kemnitzer (1989) as *Polyaspis (Polyaspis) flechtmanni*. The remaining additional mite species (see Introduction section) were collected by hand from the waste facility in Columbus Ohio. All were cleared in lactic acid and examined under Nomarski interference light microscopy. The following mite measurements were taken from drawings with a micrometer scale and expressed in microns (μm): IL = idiosomal index (Lynch 1989); BSL = basal cheliceral segment length; DSL = distal cheliceral segment length; MDL = moveable digit length; HDS = height of distal cheliceral segment; WDS = width of cheliceral distal segment; HBS = height of basal cheliceral segment; WBS = width of basal cheliceral segment; L1 = adductive ‘input’ lever arm of moveable digit; L2 = adductive ‘output’ lever arm of moveable digit; see Fig. 1. Cheliceral length (CL) was calculated as BSL + DSL. Extra articles as in some uropodines (Athias-Binche 1982) were ignored (unless marked in Tables). Only one example of a structure was used per individual mite examined. Data was stored and displayed with Excel-2011. Principal component analyses of correlation matrices used R version 3.4.4 (2018-03-15). Only DSL, MDL and HDS were directly comparable to those measures from Buryn and Brandl (1992)—respectively column one, seven and two in their “APPENDIX Table of mean values”. The idiosomal index (Griffiths et al. 1990) was used as an estimate of overall mite body size so as to avoid bias by squashing the full opisthosoma—as such it is akin to the use of the sternum in the morphometric comparison of spiders. It is acknowledged it is not ortho-iconographic (Vercammen-Grandjean 1972). IL was taken to indicate ‘size’. CL was taken to indicate cheliceral ‘reach’. MDL was taken to indicate chelal ‘gape’. Cheliceral aspect ratio was taken as CL/average(HBS,HDS). Natural logarithms were used throughout unless stated to be $\text {log}_{{10}}$.

All measurements were taken in only two orthogonal directions (on the common registration of centroids, reflection and rotational reorientation of each drawing). An estimate of the relative orthogonal allometric rescaling to give a metric space for any morphometrics was done by log-log linear regression (through zero) of each measure against the idiosomal index (IL). An average common value of slopes was then used to calculate the relative factor of horizontal (slope = 0.7366) versus vertical (slope = 0.5896) growth gradients (for the measures common between this study and Buryn and Brandl 1992). This relative factor (value = 1.249, by simple division of slopes) was used as a pre-multiplier of any height log values before correlation analysis—it is recognised that this is only a very simple approximation to partialing out size differences (see Smith 1980; Seim and Saether 1983 etc.).

The chelal velocity ratio (VR—see Davidovits 2018 for definition) for each individual was estimated as the ratio of the two moveable digit lever arms, i.e., $\frac{L1}{L2}$ on the raw data, and taken to be the theoretical mechanical advantage (MA) of the static ‘first-class lever’ of the chela (the condylar joint is assumed frictionless). Measurements of actual bite force (as in Meijden et al. 2012b) on such small arthropods is not feasible. Also as Perdomo et al. (2012) points out, the bouquet shape of the levator muscles complicates the estimation of its cross-sectional area (and therefore the force it delivers) from a two dimensional view. So, two estimates of the adductive or levator force F1 along the closing tendon were calculated from the physical measurements—assuming a pennate model, and assuming a circular model of muscle structure optimally arranged within the space available inside the whole chelicera (Fig. 1). Pinnate jaw musculature pre-adapts finches for their various specialised methods of feeding (Bowman 1961). Pinnate muscles do not swell and can contract in a confined space (Evans 1979). As in Meijden et al. (2012b), the angle of the adductive tendon was ignored to estimate the maximum theoretical force at the tip of the moveable digit when fully closed (given the tendon is positioned parallel to but halfway through the cheliceral shaft). So the pennate-assumption force $F1P=(\frac{HDS+WDS+HBS+WBS}{4*2})\cdot (CL-(1.1*MDL))$; and, the circular-assumption force $F1C=\frac{\pi }{2}\cdot (\frac{HDS+WDS+HBS+WBS}{4})^2$. The two estimates were of the same arithmetic scale per mite and were averaged ($F1AV=0.5\cdot (F1P+F1C)$), then multiplied by the velocity ratio for each individual to yield the morphologically estimated chelal “crunch force” F2AV (i.e., maximum estimated force between the tips of the moveable and fixed digit $F2AV = F1AV\cdot VR$). It is recognised that this likely overestimates the force by overestimating the physiological cross-section (PCSA—see Meijden et al. 2012b). However, such (at least) a squared model for forces has some experimental validity. Units are not ascribed to this index.

**Table 1:** Conversion of “Old measures” from previous acarological authors to those of this study (New measures)

New	“Old”	Justification	Formula
IL	Id	Regression (over *Alliphis* spp., *Veigaia nemorensis*)	$IL=0.9981*Id$
BSL	-	Regression of BSL v DSL-MDL (this study)	$BSL=0.7956*(DSL-MDL)$
DSL	“1”	Identity	$DSL= {\text{``}}1{\text{''}}$
CL	-	Addition	$CL=BSL+DSL$
MDL	“7”	Identity	$MDL={\text{``}}7{\text{''}}$
HDS	“2”	Identity	$HDS={\text{``}}2{\text{''}}$
WDS	–	Regression of WDS v HDS (this study)	$WDS=0.8280*{\text{``}}2{\text{''}}$
HBS	–	Regression of HBS v HDS (this study)	$HBS=1.0727*{\text{``}}2{\text{''}}$
WBS	–	Regression of WBS v HDS (this study)	$WBS=0.8935*{\text{``}}2{\text{''}}$
L1	–	Regression of L1 v HDS (this study)^a^	$L1=0.4076*{\text{``}}2{\text{''}}$
L2	–	Regression of L2 v MDL (this study)^b^	$L2=0.9214*{\text{``}}7{\text{''}}$
VR	–	Ratio	$VR=\frac{L1}{L2}$
L2	–	Best estimate of gape	$L2=VPMD$
VR	–	Ratio best moveable digit height:length^c^	$VR=\frac{HeightMD}{VPMD}$
CL	Cheliceral length	Identity	$CL=Cheliceral\ length$
MDL	Length of moveable digit	Identity^d^	MDL = Length of moveable digit
HDS	–	Width = Height	$HDS=WDS$
WDS	Cheliceral width	Identity	$WDS=Cheliceral\ width$
HBS	–	Identity	$HBS=HDS$
WBS	–	Identity	$WBS=WDS$
L1*	-	Regression of L1 v HDS (this study)	$L1^{*}=0.4076*HDS$
L1**	-	Trigonometry	$$L1^{**}=MDL*SIN (Angle\ of\ moveable\ digit)$$
L1	–	Best estimate average	$$\frac{L1^{*}+L1^{**}}{2}$$
L2	–	Identity	$L2=MDL$
VR	–	Ratio	$VR=\frac{L1}{L2}$
DPFD/VPMD	–	Identity	$DPFD/VPMD=\frac{Dorsal\ perimeter\ of\ fixed\ digit}{Ventral\ perimeter\ of\ moveable\ digit\ (VP)}$

*Upper* From Buryn and Brandl (1992)

^a^No extra relationship of $\frac{L1}{HDS}$ with L2 was found ie. proportion of chelicera occupied by musculature stays similar for the effective lengthening of the moveable digit

^b^Condyle position does not move as chelae elongate.

*Middle* From Adar et al. (2012)

^c^Assumes condyle is offset proportionately and analogous curvatures for DPFD and VPMD.

*Lower* From Liu et al. (2017)

^d^Slight underestimate. Calculation of F1P, F1C, F2AV, and aspect ratio follow on from Materials and methods

The morphological results of Buryn and Brandl (1992) were converted to comparable values to this study as in Table 1*Upper* (the published value for *Typhlodromus setubali* “1” was corrected to 71.0). The morphometric data set consisted of all mites from Buryn and Brandl (1992) plus the eight additional species in this study. The training data set consisted of only the mite species with designated feeding type from Buryn and Brandl (1992). The test data set comprised those without feeding type designation from Buryn and Brandl (1992) plus the eight additional species in this study. Validation datasets were constructed from Adar et al. (2012) and Liu et al. (2017) as in Table 1*Lower*, and from Hyatt (1980) as described in the text.

Generalised linear models and hypothesis tests were carried out in R using *glm* on the binary variable “worm-like prey feeder” $= 1$, “micro-arthropod feeder” $= 0$ and a *logit* link using only the historical data scored with the actual prey-type as a training set. Polyphagous and omnivorous species were scored as potentially feeding on both worm-like and micro-arthropod prey and were included in the modelling of

$$\begin{aligned} p(worm-like\ prey\ feeder\ |\ f[IL, CL, MDL, VR, F2AV]) \end{aligned}$$

or equivalently $$1-its\ value=$$

$$\begin{aligned} p(microarthropod\ feeder\ |\ f[IL, CL, MDL, VR, F2AV]) \end{aligned}$$

where *p* means probability, “|” means ‘given’ and *f* is a suitable rational function. Velocity ratio (VR) was transformed with the arcsine function ($sin^{-1}(\sqrt{(}VR))$) as a positive domain yielding approximation to the symmetric linearising *logit* function when needed. A log function (*f*[...]) was used on all variables (including the transformed velocity ratio) in line with the allometric assumption and the aim to yield isotropy for any PCA. Log transformation of bite force follows the approach of Meijden et al. (2012b). Log transforming the symmetrised velocity ratio allows for the expected different behaviour in the tails of the distribution (i.e., at different body sizes—see Results section). Then the logit link function was chosen for *g*, given $\eta =g(\nu )$ where $\eta =linear \ predictor$ and $\nu$ is the expected value for the outcome ($p(worm-like\ feeder)$) following McCullagh and Nelder (1989). Change in deviance against a null model was assessed by asymptotic $\chi ^{2}$ tests. The simplest most parsimonious model with tight distributions was sought. Conditional density plots (*cdplot* in R) of *worm-like prey feeding* as a factor were used to explore the pattern of probabilities to each measured variable. Species in the training set were predicted to examine the post-hoc consistency of the model. Predictions were then also made for each of the extra eight species (*A. halleri*, *V. nemorensis*, *G. confusa* being used for explicit validation) as well as all previously studied mites of unknown status given their data, and each mite allocated the most probable feeding type class. The validation datasets (Adar et al. 2012; Liu et al. 2017) were plotted on the resultant displays. Data for a validation cohort of parasitines was extracted from illustrations in Hyatt (1980) and means calculated as in text.

All new data generated or analysed during this study and all model specifications are included in this published article—or in compliance with EPSRC’s open access initiative are available from https://doi.org/10.5287/bodleian:NooOrQ09P.

## Results

**Table 2:** Results for extra species used in this study

Species	IL	CL	BSL	DSL	MDL	HDS	WDS	HBS	WBS	L1	L2	VR	F2AV	Aspect ratio
	(μm)	(μm)	(μm)	(μm)	(μm)	(μm)	(μm)	(μm)	(μm)	(μm)	(μm)			
This study														
* Alliphis halleri* $\female$	469.6	143.5	44.1	100.5	37.7	26.0	25.4	27.2	25.4	11.2	31.5	0.357	415.3	5.40
* Glyphtholaspis confusa* $\female$	1434.7	546.6	168.2	378.4	136.0	90.7	77.2	99.6	81.9	46.4	114.4	0.406	5867.8	5.74
* Parasitus coleoptratorum* DN	1280.5	511.6	171.3	340.3	147.3	110.9	85.7	118.6	95.4	38.4	139.0	0.276	4762.3	4.46
* Parasitus fucorum* DN	1074.3	358.5	121.7	236.8	96.6	83.2	65.3	89.2	70.0	29.8	89.1	0.334	3145.2	4.16
* Parasitus lunaris* DN	628.1	359.8	117.5	242.3	120.6	62.0	54.5	68.8	61.3	23.1	114.5	0.202	1305.3	5.50
* Polyaspis* n.sp. DN	846.4	366.6	127.5	239.1	130.4	50.8	48.6	49.5	49.5	23.8	119.2	0.199	938.0	7.31
* Uropoda orbicularis* DN	682.0	272.3	83.1	189.4	23.4	13.8	14.7	28.3	27.2	8.9	19.4	0.458	741.6	12.93
* Veigaia nemorensis * (new) $\female$	678.4	475.4	166.9	308.5	152.4	68.1	61.4	68.6	59.3	33.5	152.6	0.220	1824.2	6.95
Hurlbutt (1965, 1968)														
* Veigaia alba*		390–430			110–115									
* Veigaia bakeri*					228									
* Veigaia dendritica*		425–445			112–120									
* Veigaia bakeri*					220–235									
* Veigaia locha*		615–640			197–215									
* Veigaia mitis*					63									
* Veigaia partitus*		385–400												
* Veigaia piliseta*		450–470			117–123									

*Upper* Data table of morphometric means for the eight additional species. $n=10$ for extra studied species except $n=5$ for *Veigaia nemorensis* (new). F2AV is an indicative force for ranking species. Aspect ratio = CL/Average(HBS, HDS). *Lower* Ranges and point values from Hurlbutt (1965, 1968) for comparison with *V. nemorensis*—see also Table 7

**Table 3:** Data table of means for full analysis data set (= training + test data sets)

Species	Size	Reach	Gape	Velocity ratio	Crunch force	Aspect ratio
	IL	CL	MDL	VR	F2AV	
	(μm)	(μm)	(μm)			
*Alliphis halleri* ^a^	469.6	143.5	37.7	0.357	415.3	5.40
*Alliphis siculus*	481.1	143.9	38.0	0.745	3314.1	2.17
*Amblyseius okanagensis*	395.2	127.9	33.3	0.385	470.6	4.26
*Ameroseius* sp.	380.3	93.3	21.5	0.438	290.5	4.23
*Androlaelaps casalis*	655.8	187.2	39.6	0.361	646.0	5.59
*Arctoseius brevicheles*	404.2	147.1	25.0	0.478	584.8	5.26
*Arctoseius certratus*	344.0	129.1	32.7	0.376	434.6	4.48
*Arctoseius minutus*	341.6	112.7	24.7	0.394	304.1	4.94
*Arctoseius venustulus*	406.9	144.1	33.3	0.336	362.9	5.50
*Blattisocius keegani*	477.1	117.0	26.7	0.480	566.7	3.89
*Cheiroseius borealis*	566.4	244.0	56.7	0.256	546.7	7.18
*Dendrolaelaps foveolatus*	387.6	124.0	32.1	0.423	554.4	3.90
*Eugamasus berlesei*	1289.5	421.5	127.0	0.317	3857.8	4.47
*Eugamasus cavernicola*	1162.5	415.5	121.0	0.299	3090.0	4.91
*Eviphis ostrinus*	535.0	327.3	40.0	0.265	490.6	13.16
*Geholaspis longispinosus*	986.2	372.9	91.3	0.293	1886.7	5.95
*Geholaspis* sp.	821.2	395.6	199.0	0.215	2601.6	3.95
*Glyphtholaspis confusa* ^a^	1434.7	546.6	136.0	0.406	5867.8	5.74
*Hypoaspis aculeifer*	755.6	328.0	100.0	0.261	1472.4	5.37
*Hypoaspis angustiscutata*	797.5	347.6	107.0	0.260	1666.4	5.32
*Iphidozercon gibbus*	426.2	190.0	28.6	0.371	456.7	7.64
*Leioseius bicolor*	360.8	130.1	34.0	0.351	389.4	4.65
*Macrocheles montanus*	1252.6	448.8	126.0	0.319	4025.8	4.77
*Pachylaelaps furcifer*	940.2	328.0	91.0	0.243	1096.1	6.33
*Pachylaelaps leauchlii*	858.7	313.5	88.2	0.254	1136.3	5.97
*Pachyseius humeralis*	626.4	196.3	56.2	0.270	526.7	5.52
*Parasitus beta*	641.8	186.7	52.0	0.340	809.4	4.50
*Parasitus coleoptratorum* DN^a^	1280.5	511.6	147.3	0.276	4762.3	4.46
*Parasitus fucorum* DN^a^	1074.3	358.5	96.6	0.334	3145.2	4.16
*Parasitus lunaris* DN^a^	628.1	359.8	120.6	0.202	1305.3	5.50
*Parazercon radiatus*	349.3	141.6	31.9	0.430	617.9	4.41
*Pergamasus cornutus*	615.7	243.7	89.5	0.272	1156.4	4.28
*Pergamasus crassipes*	1144.8	430.0	167.8	0.258	3445.5	4.23
*Pergamasus digitulus*	496.3	195.8	70.8	0.278	772.9	4.25
*Pergamasus mirabilis*	769.5	417.1	158.5	0.241	2639.6	4.65
*Pergamasus misellus*	531.3	196.1	71.8	0.274	761.5	4.25
*Pergamasus oxygynelloides*	555.9	232.5	96.0	0.249	990.9	4.15
*Pergamasus quisquillarum*	1225.3	547.6	199.8	0.259	5118.9	4.52
*Pergamasus runcatellus*	693.0	285.9	112.1	0.281	1901.2	3.87
*Pergamasus runciger*	791.4	317.2	121.3	0.270	2034.1	4.14
*Pergamasus septentrionalis*	1320.8	593.7	210.7	0.286	7401.0	4.21
*Pergamasus* sp	642.0	267.0	103.2	0.275	1530.4	4.02
*Pergamasus suecicus*	447.0	204.8	75.9	0.261	747.7	4.41
*Polyaspis* n.sp. DN	846.4	366.6	130.4	0.199	938.0	7.31
*Porrhostaspis lunulata*	1034.0	401.3	123.0	0.227	1576.2	6.15
*Proctolaelaps pygmaeus*	374.2	119.2	26.4	0.468	526.4	4.12
*Rhodacarellus epigynalis*	419.3	176.9	59.6	0.385	1302.6	3.29
*Rhodacarellus sileciacus*	298.7	112.4	32.9	0.386	430.9	3.78
*Rhodacarus agrestis*	444.5	236.6	94.2	0.288	1409.9	3.72
*Rhodacarus strenzkei*	585.2	327.3	133.4	0.310	3373.3	3.38
*Trachytes aegrota*	678.3	308.6	29.9	0.271	348.7	16.27
*Typhlodromus setubali*	313.4	107.9	24.6	0.450	407.7	4.17
*Urodiaspis tecta*	726.6	289.5	22.0	0.322	338.9	17.46
*Uropoda orbicularis* DN^a^	682.0	272.3	23.4	0.458	741.6	12.93
*Veigaia cerva* ^b^	815.6	801.5	305.0	0.148	2979.9	7.58
*Veigaia decurtata*	378.1	286.0	105.5	0.161	449.3	7.17
*Veigaia exigua*	394.9	296.5	114.8	0.173	602.0	6.39
*Veigaia nemorensis* (new)^a^	678.4	475.4	152.4	0.220	1824.2	6.95
*Veigaia nemorensis* (old)	670.1	425.8	153.9	0.223	2123.5	5.29
*Zercon peliatus*	455.3	157.3	41.1	0.337	513.4	4.85

All female unless stated

^a^Extra species used in this study

^b^Taken to be the same as *Veigaia cervus* in Evans (1955). $n=1$ for data extracted from Buryn and Brandl (1992); $n=10$ for extra species except $n=5$ for *Veigaia nemorensis* (new). F2AV is an indicative force for ranking species. Aspect ratio = CL/Average(HBS, HDS)

Table 2 shows the mean measurements made for each mite of the additional species used in this study. Table 3 gives the size ( $\equiv$ IL), reach ( $\equiv$ CL), gape ( $\equiv$ MDL), velocity ratio (moveable digit adductive lever arm ratio, VR), estimated crunch force indicator (F2AV) and aspect ratio (Meijden et al. 2012b) for all species (training and test data sets). The results are interpreted below in the context that chelae close in the same way across species and that chelicerae are deployed in a identical way too. Of course in practice, as in some fish (Geistdoerfer 1977), irrespective of the design of mouthparts an animal’s trophic ecology could be correlated with how the structures are used. This behavioural flexibility is not considered here. It is recognised that the idiosomal index may still slightly bias the comparative size of (almost bi-segmented) mites like the rhodacarids compared to typical gamasines but it does make the size comparison of globular uropodoids to pear-shaped parasitids and pergamasids much more defendable.

**Table 4:** *glm* logit linear model test results for each hypothesis (labeled as in Introduction section) applied to training data set

Hypothesis	Model	$\chi ^{2}_{1}$	$p\ value$	
(i)	Size: *log*(*IL*)	0.16107	0.69	n.s.
(ii)	Reach: *log*(*CL*)	0.10709	0.74	n.s
(iii)	Gape: *log*(*MDL*)	0.56808	0.45	n.s.
(iv)	Velocity ratio: $sin^{-1}(\sqrt{(}VR))$	1.7001	0.19	n.s.^a^
	Velocity ratio: $log(sin^{-1}(\sqrt{(}VR)))$	1.8348	0.18	n.s.^b^
(v)	Crunch force: *log*(*F*2*AV*)	0.025428	0.87	n.s

$$\chi ^{2}_{1}=3.841$$ for $$p=0.05$$

^a^Note no log transform (test if asymmetric tails are a better model or not)

^b^Best model (selected for predictive algorithm)

**Fig. 2:**
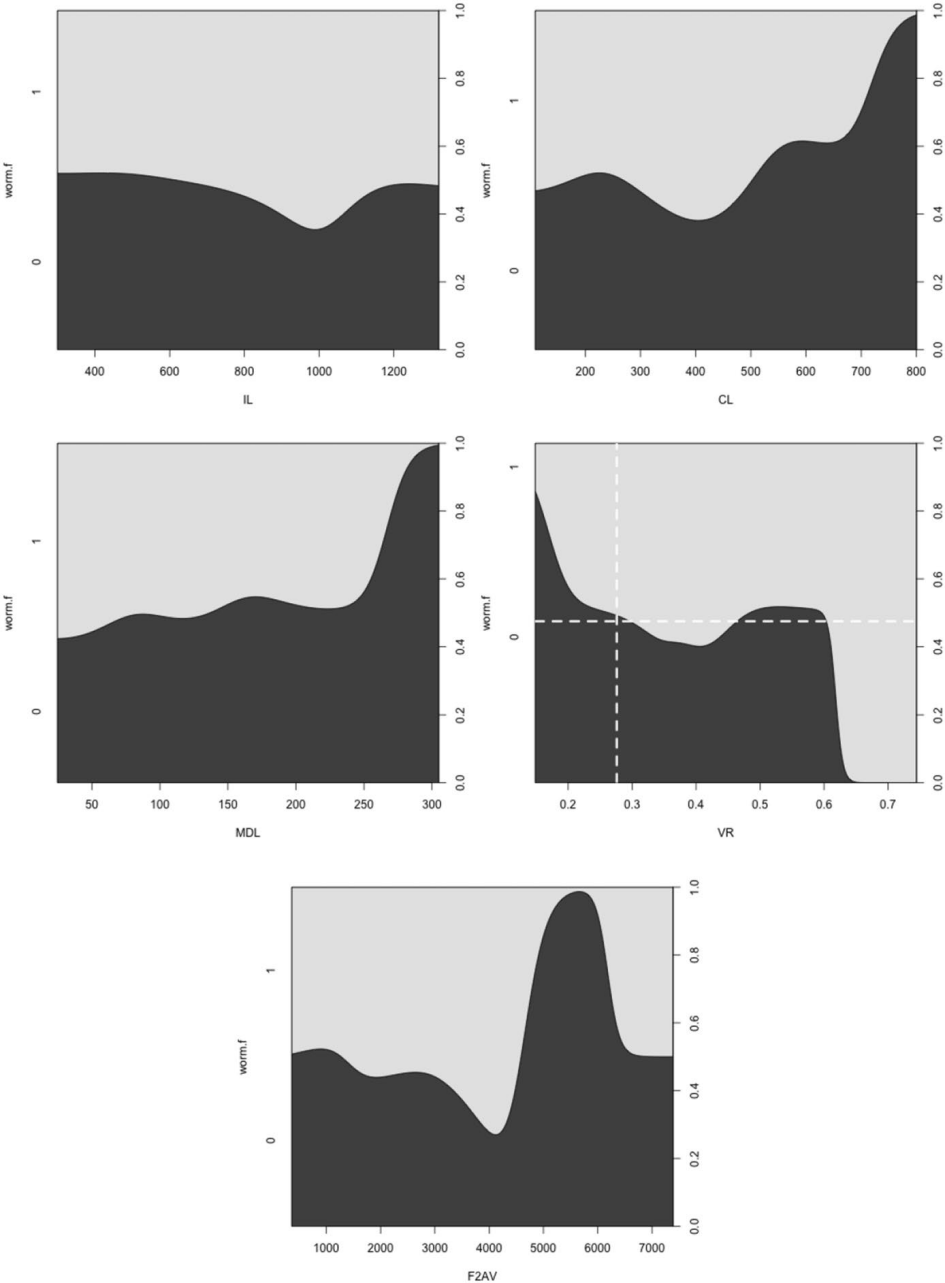
Conditional density plots of each measured variable against $worm.f=p(worm-like\ feeding)$ on Buryn and Brandl (1992) data set with known feeding type (= training data set). Pale grey zone = area of worm-like feeding. Black zone = area of microarthropod feeding. See Table 4 for explicit hypothesis tests. Velocity ratio (VR) shows strong diagonal manifold. *Top Row Left to Right* Size (IL), Reach (CL). *Middle Row Left to Right* Gape (MDL), Velocity ratio (VR) with dashed lines indicating best *glm* logit model fit threshold. *Bottom Row Left to Right* Crunch force (F2AV) indicating larger forces usually linked with micro-arthropod style of feeding

The low chelal velocity ratios typified by *Veigaia* spp. match well the 0.22–0.23 of those of the tips of the elongate chelae of some crabs (*Uca*, *Callinectes* and *Procambus*—Brown et al. 1979). Long appendages with low leverage characteristics are most effective for the capture of fast moving small prey (Gittelman 1977). The range from the highest velocity ratio that of *Alliphis halleri* at 0.745 to that of *Polyaspis* n.sp. at 0.199 compares favourably with the 2.8 times between the cutter claw and crusher claw of the lobster (Costello and Lang 1979). Table 4 gives overall test results for hypotheses (i) to (v) on the training data set. No overall model was significant due to the small sample size (39 species). The most evidence found was for a relationship between velocity ratio and the probability of feeding on worm-like prey—with an almost monotonic trend as shown in the conditional probability plot in Fig. 2. There is a trend that medium-to-large reach mites or high gape mites (Fig. 2) exhibit the micro-arthropod feeding habit. Body size in the first instance is not important per se. Allowing for different tail behaviour by log transforming the symmetrised velocity ratio gave a better fit as expected. For comparison, the velocity ratio ($\frac{l_{caLA}}{l_{LA-t1}}$) for the cryptostigmatid *Archegozetes longisetosus* from Heethoff and Norton (2009) was $\frac{25}{32}=0.7813$ This is near the jaw length moment arm (JL)/ temporalis moment arm (MAT) ratio of the means of low-crowned and high-crowned fossil horses (in Table 1 of Radinsky 1984) respectively of 0.7605 and 0.6161, indicating herbivory. The mechanical advantage values reported in saprophagous astigmatids (Akimov and Gaichenko 1976) were 0.3968 (*Carpoglyphus lactis*), 0.4571 (*Kuzinia laevis* ) and 0.5096 (*Acarus siro*). Given this, various questions and hypotheses are now examined in turn.

### Is the chelal force estimate a reasonable indicator?

The intention in this study was to produce an indicator of the likely force *in extremis* for ranking and illustration across species—not to claim that the chelal crunch force in reality is exactly proportional to the estimated one for each species. Accordingly two different assumptions regarding muscle force parametric scaling were used (see Materials and methods section). They were highly correlated for the eight additional species (F1C versus F1P: $\rho =0.962$). Doing a regression of log(IL) versus F1AV (in the style of Perdomo et al. (2012)’s supplementary information) unsurprisingly gives a strong correlation $R^2=0.592$ (*graph not shown*). At the level of the muscle fibres pulling on tendons, Alexander et al. (1981) shows that muscles can probably exert a force proportional to their $mass^{0.8}$. Regressing the estimated force F1AV for each mesostigmatid species against the $(intracheliceral\ shaft\ volume)^{0.8}$ for each yields a linear fit through zero with an $r^{2}=0.975$ (*graph not shown*). This is strong congruence (note that F1AV slightly underestimated the possible likely maximum value on the adductive tendon for the two of the larger mesostigmatids—*Pergamasus septentrionalis* and *Veigaia cerva*). At the total organism level, body weight is proportional to the cube of body length (see Perdomo et al. 2012 supplementary information for log-log fit in oribatids) and, force should be proportional to $body\ mass^{(\frac{2}{3})}$ (Alexander 1985). A power regression line fitted to F2AV versus $\text {IL}^3$ for the eight additional species gives an exponent of 0.7443. That for the species recalculated from Buryn and Brandl (1992) yields an exponent of 0.5056. Treating combining these as a question of a simple unweighted meta-analysis yields an exponent overall of 0.6250—close to the theoretical expectation. On both grounds, the indicative estimator of chelal force thus looks reasonable.

Chelal adductive force in its own right is not strongly predictive of likely feeding style in this study although for the most part high values are associated with the micro-arthropod feeding habit (Fig. 2).

A low position (more than in astigmatids) for the condyle was observed in most mesostigmatids. This together with any asymmetric triangular dentition on the mesostigmatid moveable digit would ensure that on a closing rotation, some of the vector of static crushing/slicing forces of the chela will be directed up and backwards away from the prey—facilitating the cutting effect of the retractive force of the whole chelicera. In piranhas (Shellis and Berkovitz 1976) a similar movement of posteriorly inclined dentary teeth backwards and upwards on jaw closure inside the tips of the premaxillary teeth to rest between the bases of the latter all against an essentially immobile maxilla, severs fragments of flesh from their prey. In amphisbaenians, this action traps the prey and provides further shearing forces cutting the chitinous armour of small arthropods (Gans 1974). A large moveable digit adductive lever arm L1 (for any given lever arm size L2) in a mesostigmatid chela will then both ensure effective multiplication of the chelal muscle force of such teeth as well as maintaining a moderate gap between the teeth rows of the fixed and moveable digit. A high condyle position would engender the opposite effect. Mesostigmatids do look as if they are evolved for predation.

Of course, crushing or cutting forces when the chela is open will *per force* be reduced. A more accurate estimate could be made for the likely force at various morsel size values being held by that gape through rectification with the subtended angle of the levator tendon at that point. A better estimate of the PCSA of the muscle could also be made by micro-tomography in a synchrotron beam (using methods in Heethoff and Norton 2009). Similarly, forces at each chelal tooth could have been estimated rather than just at the tip as previously done in crabs (Warner and Jones 1976). This all awaits further work focused on one or two particular species where different forces along a specific dentition is being hypothesised as pertinent in a mesostigmatid feeding action.

### Were Buryn and Brandl (1992) unlucky in their span of morphometric types used for their PCA?

**Fig. 3:**
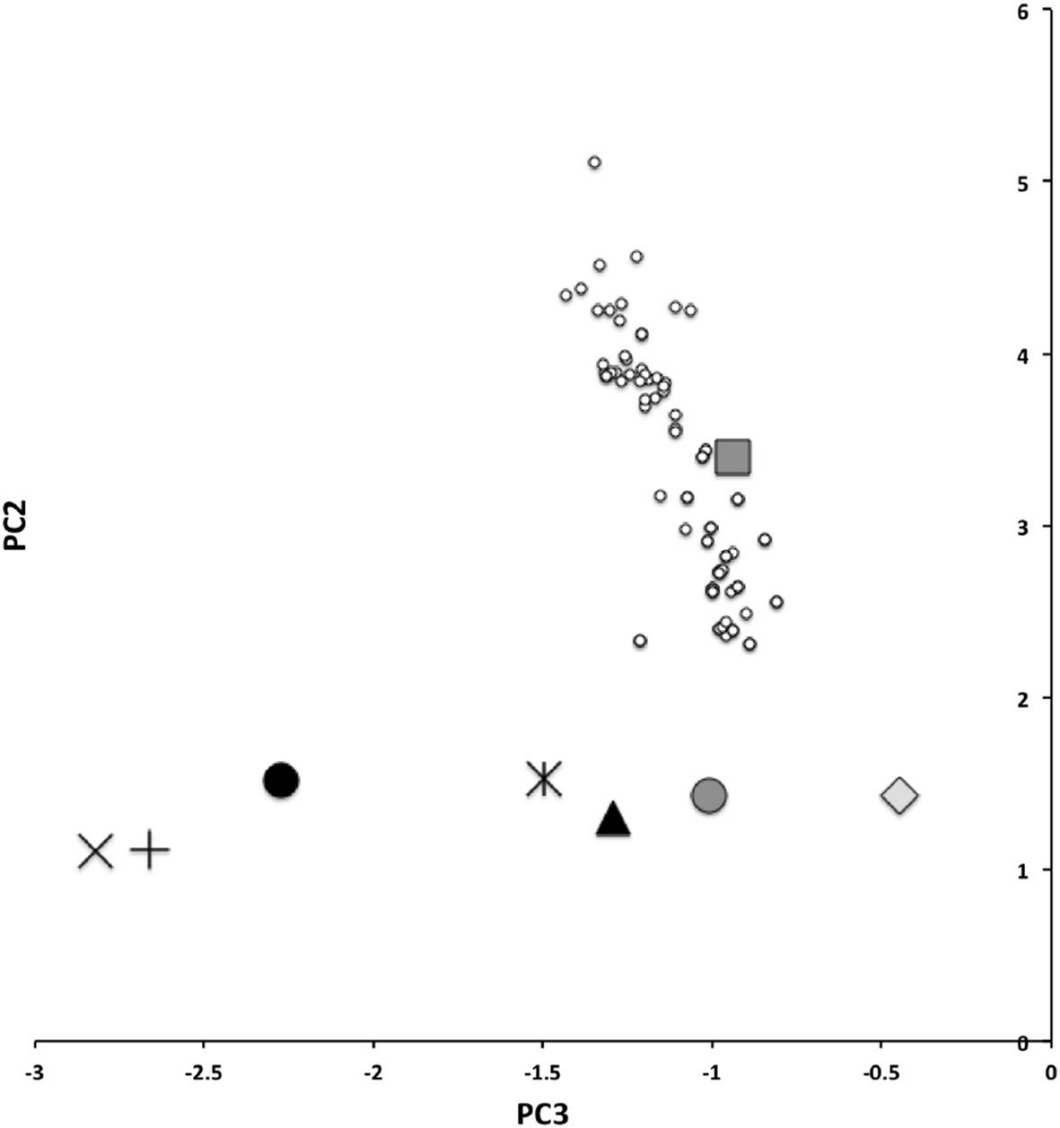
Adding extra species is useful. $PCA_2$ versus $PCA_3$ from correlation matrix of log(DSL), log(MDL) and log(HDS) mean measures over eight additional species (HDS vertical log measure inflated by 1.249) used in this study. $PC_n$ = Scaled principal components—see Table 5. Symbols as mean position of: * = *Alliphis halleri*; Black circle = *Glyphtholaspis confusa*; + = *Parasitus coleoptratorum*; x = *Parasitus fucorum*; Black triangle = *Parasitus lunaris*; Grey diamond = *Polyaspis* n.sp.; Grey square = *Uropoda orbicularis*; Grey circle = *Veigaia nemorensis* (new sample); Small open circles = where different species means from Buryn and Brandl (1992) plot in this space

Ordination of the scaled log mean values for the DSL, MDL and HDS measures over the eight species of this study (using PC2 and PC3 from a cross-species correlation analysis) for each individual specimen plus calculating the component scores for each species and genus using the (log) ordinary mean values and those from Buryn and Brandl (1992) is shown in Fig. 3 from the vectors in Table 5. PC1 was simply a large eigenvalue overall inflation factor across the eight species (in line with the basic equalised allometry). This is expected as the original and extra mites were purposely chosen to vary in size in both studies.

**Table 5:** Estimates from PCA of correlation matrix of log(DSL), log(MDL) and log(HDS) mean measures over eight additional species (vertical scales inflated by 1.249 for isotropy, see text)

	$PC_1$	$PC_2$	$PC_3$
DSL	− 0.5475885	0.8343956	− 0.06269695
MDL	− 0.5947336	− 0.335407	0.73061218
HDS	− 0.5885906	− 0.4373628	− 0.67990804
Standard deviations ($\lambda$)	1.6189	0.5543	0.26846

$PC_n$ = principal components. $\lambda _n$ eigenvalues

PC2 could be interpreted as a cheliceral shape direction—contrasting DSL growth to MDL and HDS diminution, or conversely MDL and HDS inflation to DSL shrinkage. That is, effectively if (part of) the cheliceral reach goes up, the chelicera itself gets narrow and the moveable digit smaller across the eight additional species (more than equal allometry would suggest). Or conversely, moveable digits getting bigger occurs when the chelicera gets taller and the distal part of cheliceral reach gets smaller in the eight species (more than equal allometry would suggest). This matches a model of the partitioning of finite resources during the evolution of the gnathosoma. For their size (PC1), six out of the eight extra species in this study have markedly unusual designs in this way (for this sample of these three morphological measurements) than those three of Buryn and Brandl (1992). Even *A. halleri* and *U. orbicularis* appear somewhat distinct for their size (PC1). The size of *V. nemorensis* is similar across the two studies but its position in the pattern of character covariation is not. This all points to the extra utility of including these new taxa in this review.

PC3 here could be interpreted as an ‘aspect ratio’ contrast of MDL versus HDS. That is, cheliceral height increasing at the expense of moveable digit length and vice versa (over and above what equal allometry would suggest). Again this fits a model of finite resource partitioning during the evolutionary control of development. If HDS is a limiting factor for the magnitude of the in-lever moment arm L1—then this component is indicating something to do with mechanical advantage of the chela (arising from its velocity ratio)—MDL indicating the out-lever moment arm L2. At least three of the eight additional species (*G. confusa*, *P. coleoptratorum* and *P. fucorum*) show markedly different values than those studied by Buryn and Brandl (1992) on this axis.

However the exact interpretations of what PC2 and PC3 are, is *not* of great consequence at this point—the issue is that the *pattern* of covariation over the additional species captures something more than the almost linear arrangement of all the species examined by Buryn and Brandl (1992) (that is, along these two components for these three morphological measurements which were exactly in common over the two studies). It would appear that Buryn and Brandl (1992) may have been somewhat unlucky. While *U. orbicularis* is broadly similar to the previous species studied, the act of including specimens of *G. confusa*, *P. coleoptratorum* and *P. fucorum* in this study brings something distinctly different in cheliceral design (as does *A. halleri* and *P. lunaris* somewhere in between them). This span of extra morphotypes across the eight species appears useful as an addition.

### What does simple morphology indicate?

One expects a relationship between diet and trophic structures—if only at the most general level (Rotenberry 1980). So, rather than complicated morphometrics, simply examining the conditional density plots of the training data set (Fig. 2) does confirm that

- overall size *is unrelated* to feeding type—hypothesis (i) ✓;
- feeding type *is not related* to reach (except for very large chelicerae) *nor related* to gape (except for very large chelae)—hypotheses (ii) ✓ and (iii) ✓;
- feeding type *is related* to chelal velocity ratio at low and high values—hypothesis (iv) ✓; and,
- feeding type *is in a relationship* with chelal crunch force (but in a complicated way)—hypothesis (v) ✓.

The underlying assumptions of this study are *all* supported.

Alexander et al. (1979) points out that if animals of different sizes were geometrically similar to each other, then the lengths and diameters of corresponding structures like limb bones would be proportional to $body\ mass^{0.33}$. Conversely if they were elastic similar (as in McMahon’s theory), lengths would be proportional to $body\ mass^{0.25}$ and diameters to $body\ mass^{0.38}$. The mesostigmatids were not weighed in this study but given an assumption of a uniform density such could be considered estimated as $IL^3$. A power regression over species of this versus various mesostigmatid measures gives: CL $exponent=0.358, R^{2}=0.735$; MDL $exponent=0.415, R^{2}=0.518$; L2 $exponent=0.415, R^{2}=0.518$; average (HBS, WBS, HDS, WDS), i.e., cheliceral diameter $exponent=0.297, R^{2}=0.471$; L1 $exponent=0.297, R^{2}=0.471$ None of these are strong fits and some measures capture essentially the same information as others, however, it does suggest that comparatively more elongation than even geometric similarity is present over this set of species. Further that the moment arm L1 scales like a diameter (much as would be expected if contained under a gnathotectum in a cylindrical gnathosoma).

Recalling that body size (or weight) is related to prey size in organisms (Hespenheide 1973), rather than doing an analytical size adjustment (as in Buryn and Brandl 1992), it is illuminating to next look at the simple relationships of gape, reach and mechanical-model derived crunch force with preferred food type categorised by body size (IL) class (as a surrogate for the length of leg 1; Usher and Bowring 1984). One is not making a morphometric assertion, rather by controlling body size into ’bins’ this means that one is comparing mite designs under conditions of approximately equal access to prey locations or an approximately equal ability to grab prey of the same size (i.e., within a predation functional group). Larger mites ought to be able to grab vagile prey at a distance and not rely upon proximity for predation success. Is this the case?

**Fig. 4:**
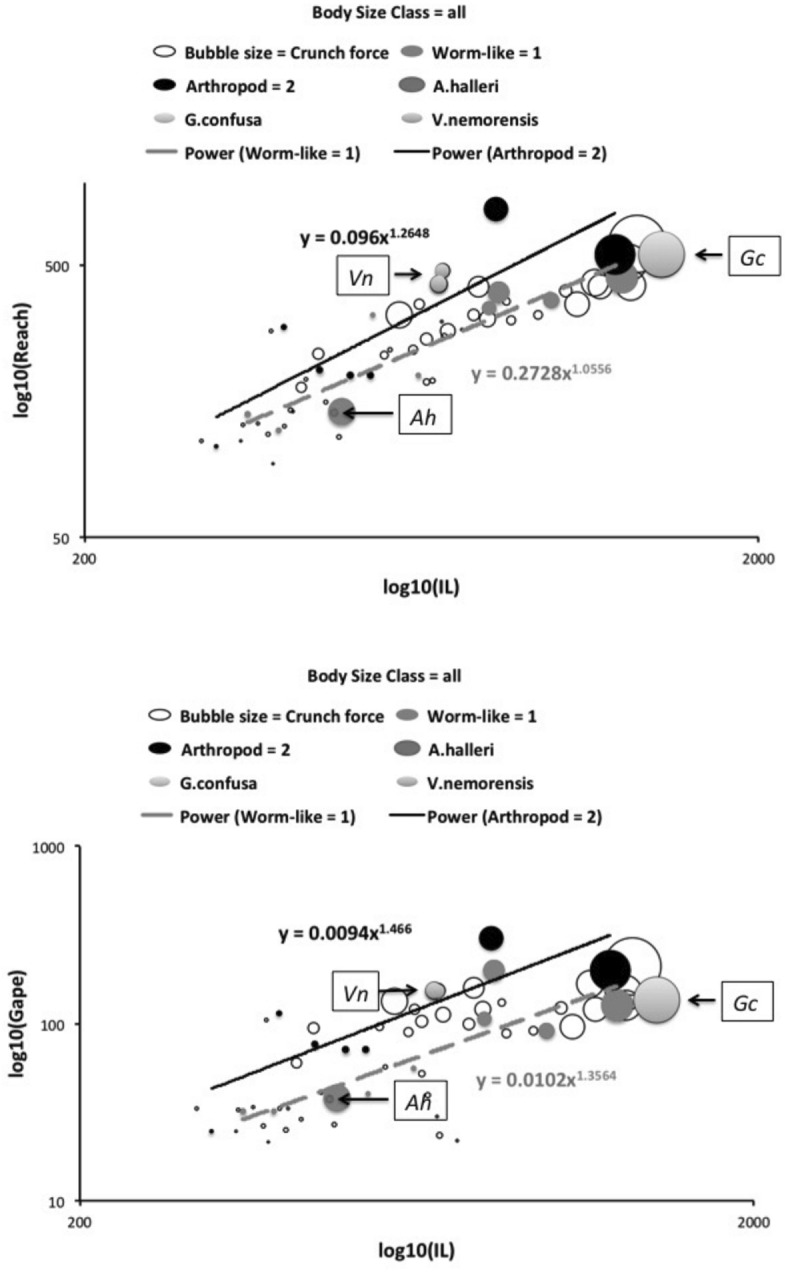
Validating historical conclusions using this study + data from Buryn and Brandl (1992). Bubble size is indicative chelal crunch force (F2AV). Grey circles and grey dashed line = worm-like prey; Black circles and black solid line = microarthropod prey. Note gap between lines confirms Buryn and Brandl (1992)’s morphometric result. Also note agreement with three extra species of known feeding type (*Ah* = worm-like prey feeder; *Vn* = microarthropod feeder; *Gc* = insect larva/eggs feeder i.e., worm-like prey). *Upper*: Plot of cheliceral reach (CL) versus idiosomal index (IL) on log log scale with separate regression lines for each feeding type. All data equation $y=0.3003x^{1.0472}$ *Lower*: Plot of chelal gape (MDL) versus idiosomal index (IL) on log log scale with separate regression lines for each feeding type. All data equation $y=0.0338x^{1.1866}$

**Fig. 5:**
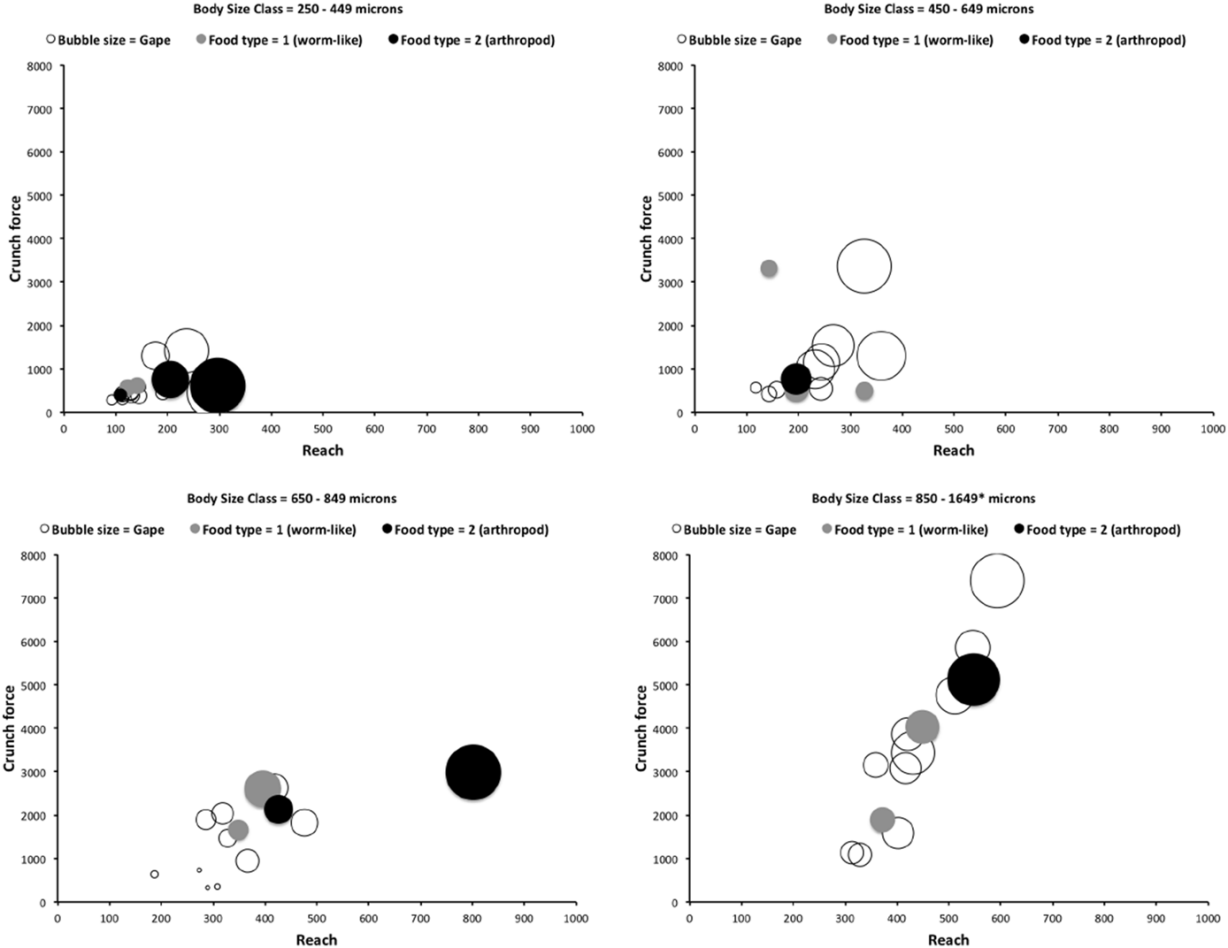
Cross-checking historical conclusion. Plot of chelal crunch force indicator (F2AV) versus cheliceral reach (CL) categorised by idiosomal size class (this study + from Buryn and Brandl 1992). Grey circles = worm-like prey feeders; Black circles = microarthropod prey feeders). Bubble size is chelal gape (MDL). Relationships emerge with size increase ($450\text {-}649\,\mu \text {m}$ and above). Large mites with large reach and powerful bite tend to have a slicing/cutting feeding habit and a big gape

Firstly, overall neither reach (CL) nor gape (MDL) are strongly allometric (Fig. 4).Unlike in spiders (Lockett 1932) linear body scaling is acceptable if needed. Secondly, Fig. 5 shows that even though a clear relationship of crunch force F2AV only appears at large mite sizes, within each body magnitude class (except in the general mid-range 450-649 μm IL range), moving from a worm-like crushing-kill predation habit to a micro-arthropod slicing/cutting-killfeeding habit infers a bigger reach, a larger gape and an increased estimated chelal crunch force. The same conclusion arises if one plots the data having swopped the bubble size values for the x-axis values. These trophic relationships with reach and gape are consilient with the argument around relative sizes and food type in Buryn and Brandl (1992). Furthermore, their results are nicely confirmed by Fig. 4 where *A. halleri* plots amongst the worm-like feeders, *Veigaia nemorensis* (both studies) plots squarely upon the micro-arthropod feeding regression line and *G. confusa* is on the far right hand side where a mite could be facultatively either feeding type. While the body size measurement in Adar et al. (2012) and Liu et al. (2017) is not exactly the same, the phytoseiids nicely plot in the top left sub-panel of Fig. 5 (*result not shown*). In a further study it would be useful to see if pedipalp length matched accordingly. Simple morphology *is* useful. It is expected, as in finches (Bowman 1961), that mites with less specialised mouthparts (and thus here a less specialised size—large or small) should have the more generalised diet. Of course even amongst generalist designed mites, some may be opportunistic feeders and others prey specifically on particular prey taxa for other nutritional or habitat driven reasons (Polidori et al. 2010).

### What does physics tell us about trophic design?

Examining the relationships of mechanical-model derived chelal crunch force (F2AV) and chelal velocity ratio (VR) with preferred food type against body size (IL) class (as a surrogate for the length of leg 1) offers an even simpler explanation than that above (and one amenable to developing a simple predictor for a field ecologist to use).

**Table 6:** Phytoseiid validation. Buryn and Brandl (1992) values added for comparison from Table 8

Source	Feeding group	Species	DPFD/VPMD	VR	p (worm-like feeding)	F2AV	Aspect ratio
Adar et al. (2012)	Generalist	*Amblyseius largoensis*	0.903	0.472	0.672		
Adar et al. (2012)	Generalist	*Amblyseius swirskii*	0.928	0.485	0.684		
Adar et al. (2012)	Generalist	*Kampimodromus aberrans*	1.139	0.387	0.593		
Adar et al. (2012)	Generalist	*Neoseiulus cucumeris*	0.899	0.442	0.646		
Adar et al. (2012)	Generalist	*Phytoseius plumifer*	1.077	0.376	0.582		
Adar et al. (2012)	Generalist	*Typhlodromus exhilaratus*	1.052	0.375	0.581		
Adar et al. (2012)	Generalist	*Typhlodromus pyri*	1.029	0.408	0.614		
Liu et al. (2017)	Generalist	<various>	0.925	0.460	0.662	556.9	4.02
Adar et al. (2012)	Pollen	*Euseius ovalis*	1.164	0.362	0.568		
Adar et al. (2012)	Pollen	*Euseius scutalis*	1.17	0.349	0.555		
Liu et al. (2017)	Pollen	*Euseius utilis*	1.108	0.443	0.646	454.3	3.17
Adar et al. (2012)	Pollen	*Iphiseius degenerans*	1.069	0.418	0.623		
Adar et al. (2012)	Specialist	*Neoseiulus californicus*	0.885	0.550	0.735		
Adar et al. (2012)	Specialist	*Phytoseiulus longipes*	0.963	0.397	0.604		
Adar et al. (2012)	Specialist	*Phytoseiulus persimilis*	0.932	0.436	0.640		
Liu et al. (2017)	Specialist	<various>	0.915	0.454	0.657	347.4	4.06
Buryn and Brandl (1992)	?	*Amblyseius okanagensis*	–	0.385	0.597	470.6	4.26
Buryn and Brandl (1992)	?	*Typhlodromus setualbi*	–	0.450	0.645	407.7	4.17

See Liu et al. (2017) for species included in groupings labeled as <various>. Aspect ratio = CL/Average(HBS,HDS)

**Fig. 6:**
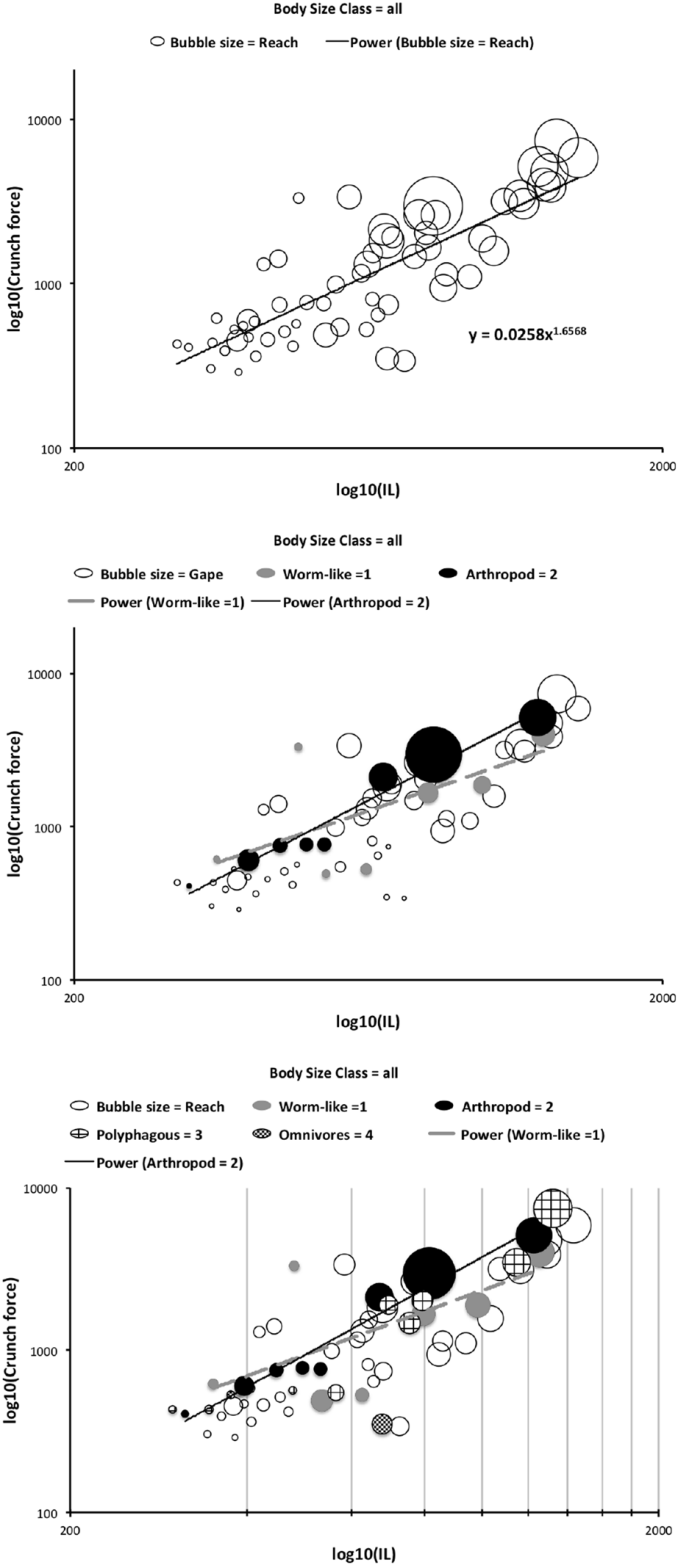
Plot of estimated chelal crunch force (F2AV) versus size (IL) for mesostigmatids (this study + from Buryn and Brandl 1992). *Upper* Common log log regression line. Bubble size is cheliceral reach (CL). *Middle* Regression lines fitted to known feeding preferences (grey circles and grey dashed = worm-like prey; black circle and black solid = microarthropod prey). Bubble size is chelal gape (MDL). *Lower* Polyphagous mites (code = 3) filled with open squares pattern. Omnivore mites (code = 4) filled with spotted pattern fill. Bubble size is cheliceral reach (CL). Vertical lines at 200 μm intervals in idiosomal length (IL). Note regression lines cross in 450–649 μm range

Looking across body sizes, unsurprisingly, small mites have small estimated adductive chelal forces, large mites dramatically stronger ones. In fact, mites with a large cheliceral reach have very large estimated chelal crunch forces that are proportionately larger than simple allometry would suggest (Fig. 6*Upper*). This suggests a runaway process to allow the equivalent of ’bone-cracking’ (and perhaps carcass scavenging as in hyaenids) in these mites. Could this differential power be the clue as to how ologamasids can attack armoured oribatids (Walter and Proctor 2013)? Table 6 validates that phytoseiids have consilient chelal crunch force F2AV values in the range 350–550. Mites with an idiosomal index (IL) more than approximately 500 μm (which note sits in the generalist 450–649 μm IL range and equals the crossing point of regressions in Fig. 6*Middle*) show clear trophic adaptation - with arthropod feeding habit mites exhibiting markedly larger forces (note the log scale). Further work should assess to what degree chitinous strengthening to cope with any increased cheliceral stresses and strains are present. The larger polyphagous mites (Buryn and Brandl (1992)’s code = 3) *Pergamasus runcatellus*, *Hypoaspis aculeifer*, *Pergamasus runciger*, *Pergamasus crassipes*, *Pergamasus septentrionalis*—sit nicely between the two feeding type regression lines (Fig. 6*Middle*) suggesting a compromise design—as Buryn and Brandl (1992) would have hoped. Smaller mites than these have estimated chelal crunch forces suitable in general for the toughness of either (or both) types of prey food at that size of prey capture or that size of prey access. Amongst these smaller size mites, there is a noticeable group of small chelal gape species with low reach and particularly low chelal crunch force values (Fig. 6*Middle*). These look like small fragmentary feeders. They contain the only two omnivorous (Buryn and Brandl 1992’s code = 4) species examples—*Proctolaelaps pygmaeus* and *Trachytes aegrota* (see Fig. 6*Lower*). Hypothesis *(v)* is supported in general. Although surprisingly hypothesis *(v)* is the worst fit model overall (Table 4), the two low crunch force moderate size mites with moderate reach are the omnivore *Trachytes aegrota* and the unknown prey feeding *Urodiaspis tecta*. This can be traced back to the complicated relationship with $p(worm-like\ feeding)$ (see Fig. 2).

**Fig. 7:**
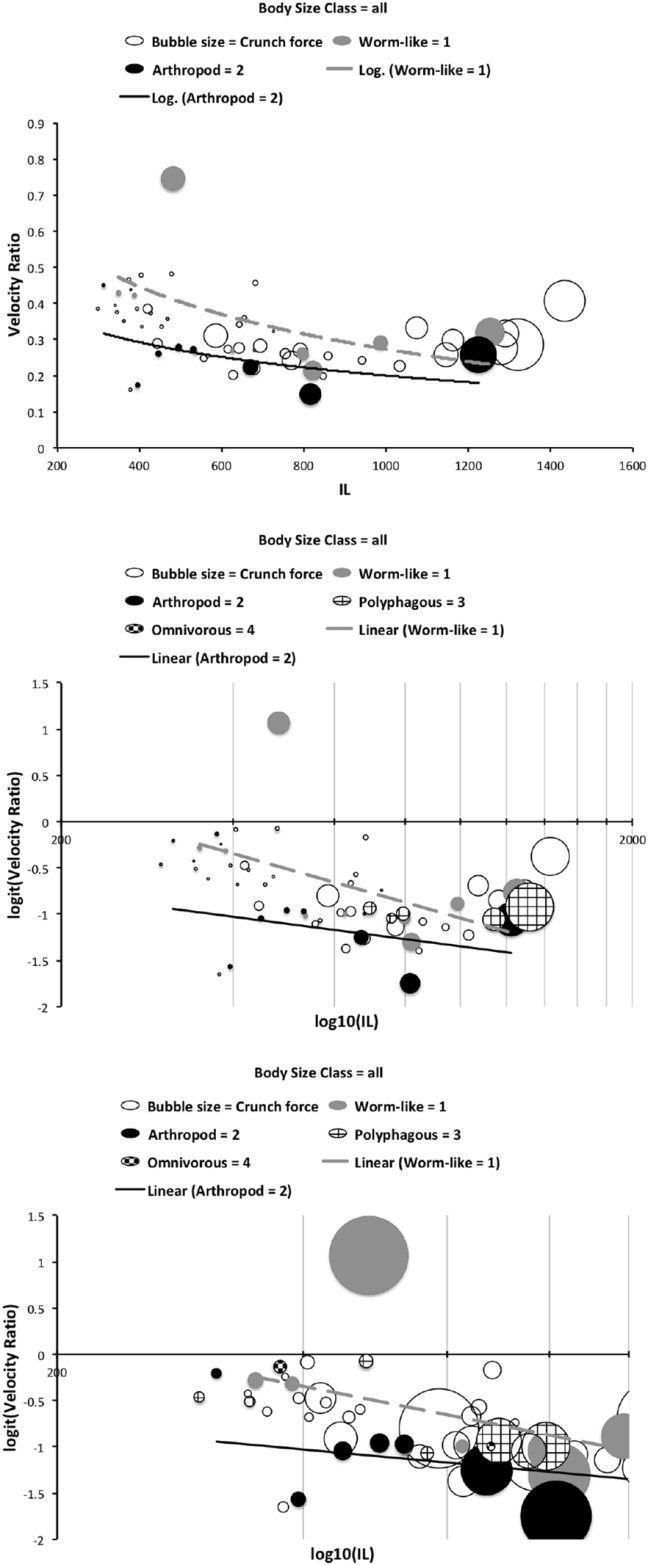
Small mites are interesting. Plot of chelal velocity ratio ($VR=\frac{L1}{L2}$) versus body size (IL) for mesostigmatids (this study + from Buryn and Brandl 1992). Bubble size is estimated chelal crunch size (F2AV). *Upper* VR versus IL. Log regression lines (*simply for illustration*) fitted to known feeding preferences (grey dashed = worm-like prey; black solid = microarthropod prey). *Middle* Logit(VR) versus $\text {log}_{10}(IL)$. Vertical lines at 200 μm intervals in idiosomal index (IL) for clarity. Polyphagous mites (code = 3) filled with open squares. Vertical lines at 200 μm intervals in idiosomal length (IL). Linear regression lines (*simply for illustration*). *Lower* Zoomed in logitVR) versus $\text {log}_{10}(IL)$. Polyphagous mites (code = 3) filled with open square pattern. Omnivore mites (code = 4) filled with spotted pattern fill. Note no clear distinction for such mites. Linear regression lines and logit without $log(sin^{-1}\sqrt{}())$ inner transform (*simply for illustration*)

Just as Perdomo et al. (2012) found for ’leverage’ (their MH/ML ≈ VR), velocity ratio in itself overall is not strongly associated with idiosomal index size - $R^2=0.097$ Fig. 7*Upper*, (or for log(IL) - $R^2=0.146$ *graph not shown*). There is an indication that larger mesostigmatids had lower velocity ratio values and in particular very small mesostigmatids had particularly high values. As expected, mesostigmatids focusing on potentially vagile micro-arthropod prey rather than worm-like prey (that cannot necessarily scoot away quickly), have lower velocity ratios in their chelae (Fig. 7*Upper*). Note how the fitted lines converge from an initial state of marked distinction at small idiosomal magnitudes. This is to be expected. If as a human, one can eat large tough nuts ($\equiv$ chitinous arthropod for a mesostigmatid), it is trivial for one to eat a squishy banana ($\equiv$ worm-like prey for a mesostigmatid) of the same size. Similarly, if as a human, one can eat a large thick skinned fruit ($\equiv$ big worm-like prey for a mesostigmatid), one can make a good attempt at chewing on a old tough steak ($\equiv$ medium size arthropod).

Small mites, however, have the issue not just of prey toughness but also one of prey escape—especially if they are both of similar size. All other matters being equal, the tip of a jaw with a low velocity ratio in snapping shut must move a greater distance (travel faster) and suffer a greater centripetal force than one with a high velocity ratio. For an analogy, consider a children’s chair swing-ride carousel at a fairground—the outer chairs suspended by chains move faster and rise higher for the same angular rotation of the central axle of the carousel engine. Small mites with elongate chelae would *per force* have a weak bite even if it closed quickly therefore struggling prey could escape. However, a jaw closing on a slow moving worm can take its time to close fairly safely with little chance of the prey escaping. Once prey are large (and so the predator’s magnitude is also large)—both soft and hard prey can be dealt with as needed (cf. a lion can chew on a softer antelope hide as much as on a harder elephant hide if it can bite the latter effectively). Conversely when prey are small and so any mesostigmatid predator (only restricted to that size small prey) is itself small (and thus itself has a small length of leg 1), such a soft-feeding specialist is limited trophically. Consider such an animal as a shrew—it cannot tackle hard seeds that an equally sized rodent could. Equally for its jaw to snap shut quickly when faced with vagile prey, at least its incisors or canines must move rather fast ($\equiv$ low velocity ratio VR, hypothesis *(iv)*). As is said, one should pay attention to small organisms (Paine 1996).

Polyphagous species plot as expected within the two regression lines (or at their approximate fusion point of IL = around 1300 μm)—see (Fig. 7*Middle*). Plotting velocity ratio by idiosomal size class in bins like Fig. 5 (*not shown*) shows that now the swop in feeding habit from worm-like prey to micro-arthropods is matched by a fall in velocity ratio for *all* size classes. Hypothesis *(iv)* is supported. Fitting $p(wormlike\ feeding)$ with a $log(sin^{-1}(\sqrt{(}VR)))$ binomial model gives the best evidence of relationship (Table 4—even better than without the log transform) in line with the conditional probability plot (Fig. 2). This is further validated by the fact that Hirschmann (1956) pointed out that an increase in height of the moveable digit (and therefore the lever arm L1) as its length shortens (and thus the lever arm L2 diminishes) in comparison to predatory forms was an adaptation to crack spores when comparing the sporophage *Pseudouropoda ovalis* to such predatory forms. Furthermore, such a shift in velocity ratio compared to the predators in this study is shown too in the known omnivore (Buryn and Brandl (1992)’s code = 4) *Proctolaelaps pygmaeus*. Similarly, comparing the two chelal forms within the crab *Macropipus depurator* shows that increased mechanical advantage is driven by altering L1 not L2 (Warner and Jones 1976). This is consilient with Perdomo et al. (2012)’s view that their ’effort arm’ ($\equiv$ to L1 herein) is the important trait in oribatid decomposers. Crushing chelae are tall with long in-levers ( $\equiv$ L1) in brachyuran crabs (Schenk and Wainwright 2001). By analogy to Hirschmann (1956), the velocity ratio values in Table 6 validate that phytoseiids must be non-vagile prey feeders (tetranychid, eriophyid and tarsonemid mites are slow movers and are searched for rather than chased). In that way phytoseiids may use their pedipalps to pin down their more sessile prey much as Muraoka and Ishibashi (1976) describes in nematophages. Physics is helpful.

### Does reach and gape modify any design conclusions?

Small gape could be prey limiting for mites as it is for snakes (Rodríguez-Robles 2002). Particularly large reach and particularly large gape is strongly associated with micro-arthropod feeding (see black zones in Fig. 2). However, as the Introduction states, it is not clear why departures from the expected relative reach or the expected relative gape values (parameters deployed by Buryn and Brandl 1992) should be *biologically* important in determining predator performance—yet it is inferred by the almost constant gaps between the regression lines in Fig. 4. Although one could suggest that an extra elongate or an extra abbreviated MDL with overall body size (i.e., non-proportionality in gape) might have a function (see Introduction), why a micro-arthropod predator for its size should have *extra* elongate chelicerae than a worm-like prey feeder of the same body size is not clear in the first instance. Perhaps excessive reach is an adaptation to ambush larger prey from longer distances like in sleeper fish (Maie et al. 2014) without alerting them to actively escape by the predator’s proximity? Or perhaps micro-arthropod cuticle size-for-size is thicker than ’worm-like’ prey integuments and would limit access to short reach chelae (much like the limitation of quill thickness on successful feeding in syringophilid mites; Casto 1974). Perhaps the large-gape, large-reach mesostigmatids are matching the design of the elongate maxillae in dacetine ants which prefer to feed upon Collembola with well developed furculae that can jump to safety quickly (Brown 1950; Wilson 1950).

Despite the larger cheliceral attack radius for such an ’excessive-reach’ mite, there are downsides to a large reach (Dalrymple 1979b)—how can you repack such long chelicerae back into the idiosoma in order to get food morsels to the pre-oral groove? Integration of any elongate cheliceral/chelal shape into the often flattened ’straitjacket’ of a uropodoid mite carapace for sure is an evolutionary challenge. The chelicerae are seemingly not folded like the necks of some turtles on retraction back into the body. Perhaps excessive gape represents some run-away developmental pathway of investment into a long carving-knife style cutting surface for a ‘slashing’ perpetrator slicing its prey anywhere and everywhere? Certainly piranha fish have a gape as much as 10% of their body size (Shellis and Berkovitz 1976). This contrast between a ‘small mouth’ (i.e., small gape) and limited ‘jaw’ protrusion (i.e., limited cheliceral reach) versus the opposite combination is precedented in animal ecomorphology—for instance it defines scarid fishes from wrasses (Wainwright et al. 2004). Those darter species (Carlson and Wainwright 2010) with the shortest jaws ($\equiv$ CL) and tiniest mouths ($\equiv$ MDL) are expert manipulators of small size benthic surface gleaned prey. Is thus *Ameroseius* sp. in Table 3 such a gleaner? Perhaps excessive gape has also to do with holding large eggs during oviposition (Marquardt et al. 2013a) or at least assisting with pedipalps in their manoeuvring from the genital area to deposition in front of the mite (Marquardt et al. 2013b) a common behaviour in mesostigmatids.

Long reach has an up-side opportunity. The elongate chelicerae of dermanyssids are claimed to have characteristic middle article flexures (Akimov and Yastrebtsov 1988). Could this function be able to move the chela deep inside a blood-oozing wound? Holothyrids can flex the middle article of their chelicera at an almost a right-angle to their body (Evans 1992). Such putative ancestral prehensile types of chelicerae Van der Hammen (1970b) also found in opilionids, palpigrades and opilioacarids and contrasts with the ‘raptorial-style’ of most acarines (Van der Hammen 1977b). In the Uropodina, the muscle group originating in the basal article which insert on the proximal edge of the middle article are claimed to be cheliceral rotators by Woodring and Galbraith (1976). Could such action sweep food towards the labrum far behind the extruded chela, or at least dramatically wiggle the chela by large amounts to avoid it getting stuck in material it had been thrust deeply into?

Whether achieved by excessive reach or excessive gape, this all indicates a ‘probing’ competency like in percine fish (Carlson and Wainwright 2010) or an ‘excavation’ competency (as in finches; Bowman 1961). Spiders of several families excavate tunnels in the ground (Bristowe 1954). Robaux et al. (1977) reports the astigmatid mite *Tyrophagus putrescentiae* to be a geophage constructing pores and aerating the substrate. Indeed, some non-uropodine mites, e.g., *Cheiroseius borealis*, *Iphidozercon gibbus*, *Pachylaelaps* spp., *Porrhostapsis lunulata* and most *Veigaia* spp. (Table 3) have very large cheliceral aspect ratios suitable for ‘hoeing’. In uropodines (Evans 1972), the often elongate attenuated second cheliceral segment may be provided with a taenidia-like supporting skeleton. If these were solely annular in form—as they seem to be in *Uropoda orbicularis* (and *Uropoda agitans*; see Gorirossi 1955a)—then they would also facilitate cheliceral shaft bending (much like the children’s spring toy $SLINKY^{TM}$—https://en.wikipedia.org/wiki/Slinky). Such effectively protrusible bendable mouthparts would offer an advantage to any ‘benthic’ surface feeder (like in fish; Alexander 1967) as substratal food could still be brought up to the general labral/pre-oral groove area, at the last minute, for ingestion ‘straight-on’. Could some uropodoid chelicerae be designed like the flexible tube on a upright vacuum cleaner used on floors and carpets?

According to Karg (1971) some species of *Cheiroseius* consume nematodes, acarid mites and immatures of Cryptostigmata. Perhaps elongate chelicerae with firmly closed chelae can be ‘fired out’ very quickly to stab active prey and the chelae then opened and closed deep inside the victim. Perhaps this is how predatory macronyssids feed (Scott and Blynn 1951; Radovsky et al 1997) slicing tissues internally? Perhaps these mites can move their whole chela and chelicera rapidly like a snake striking as has been analogously posed for plesiosaurs feeding on fish (Anon 2019). Perhaps such uropodine mite species designed like this are carrion feeders like long necked vultures, accessing deep into the carcass? Certainly their cheliceral aspect ratio (Table 3) shows that uropodines have markedly elongate chelicerae compared say to compact solifugids (aspect ratio = 1.95 in *Rhagodes melanus*, 2.41 in *Galeodes* sp.; Meijden et al. 2012b). Mammalian predators of herbivores consume the intestines first where vegetable matter is being digested (which is a source of useful vitamins; Flechtmann and McMurtry 1996). Is this what carnivorous uropodines are accessing deep inside their prey? Carrion feeders should not show adaptations to subdue struggling prey (like strong tight articulations). Is this the case for most uropodoids? *Phyllodinychus* spp. (Dinychidae) too have very elongate micro-chelate chelicerae; Krantz (1971). What do they feed on? Detailed feeding observations are needed on such mites.

**Fig. 8:**
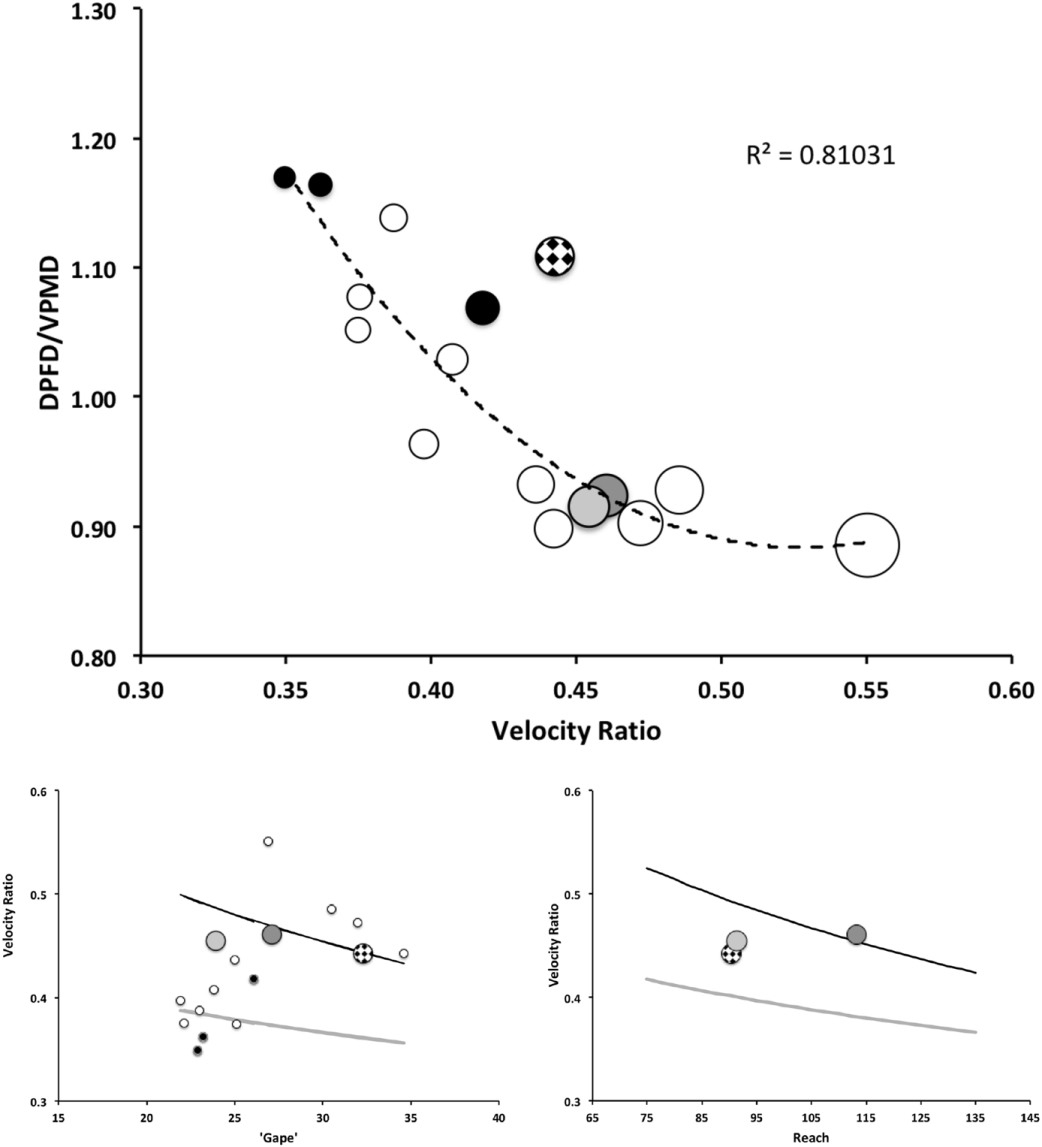
Validation with phytoseiids—they are designed as expected. *Upper* Bubble plot of DPFD/VPFD versus velocity ratio VR (bubble size = estimated $p(wormlike\ feeder)$. Open circles = predatory species from Adar et al. (2012). Black circle = pollen feeders from Adar et al. (2012). Circle with dotted interior = pollen-feeding *Euseius utilis* from Liu et al. (2017). Pale grey circle = position of specialist predatory group (*Phytoseiulus persimilis, Neoseiulus californicus, Neoseiulus pseudolongispinosus*) from Liu et al. (2017). Dark grey circle = position of generalist predatory group (*Neoseiulus barkeri, Neoseiulus bicaudus, Neoseiulus cucumeris, Neoseiulus orientalis, Amblyseius swirskii, Amblyseius tsugawai*) from Liu et al. (2017). All data in Table 6. Dotted line is quadratic trend for illustration only. *Lower left* Relationship of velocity ratio with chelal gape from Adar et al. (2012) and Liu et al. (2017) including solid black line regression for micro-arthropod feeders and solid grey regression line for worm-like feeders from Fig. 10. *Lower right* Relationship of velocity ratio with cheliceral reach for position of group data from Liu et al. (2017) including solid black line regression for micro-arthropod feeders and solid grey regression line for worm-like feeders from Fig. 10. Circle colours as in *Lower left*

**Fig. 9:**
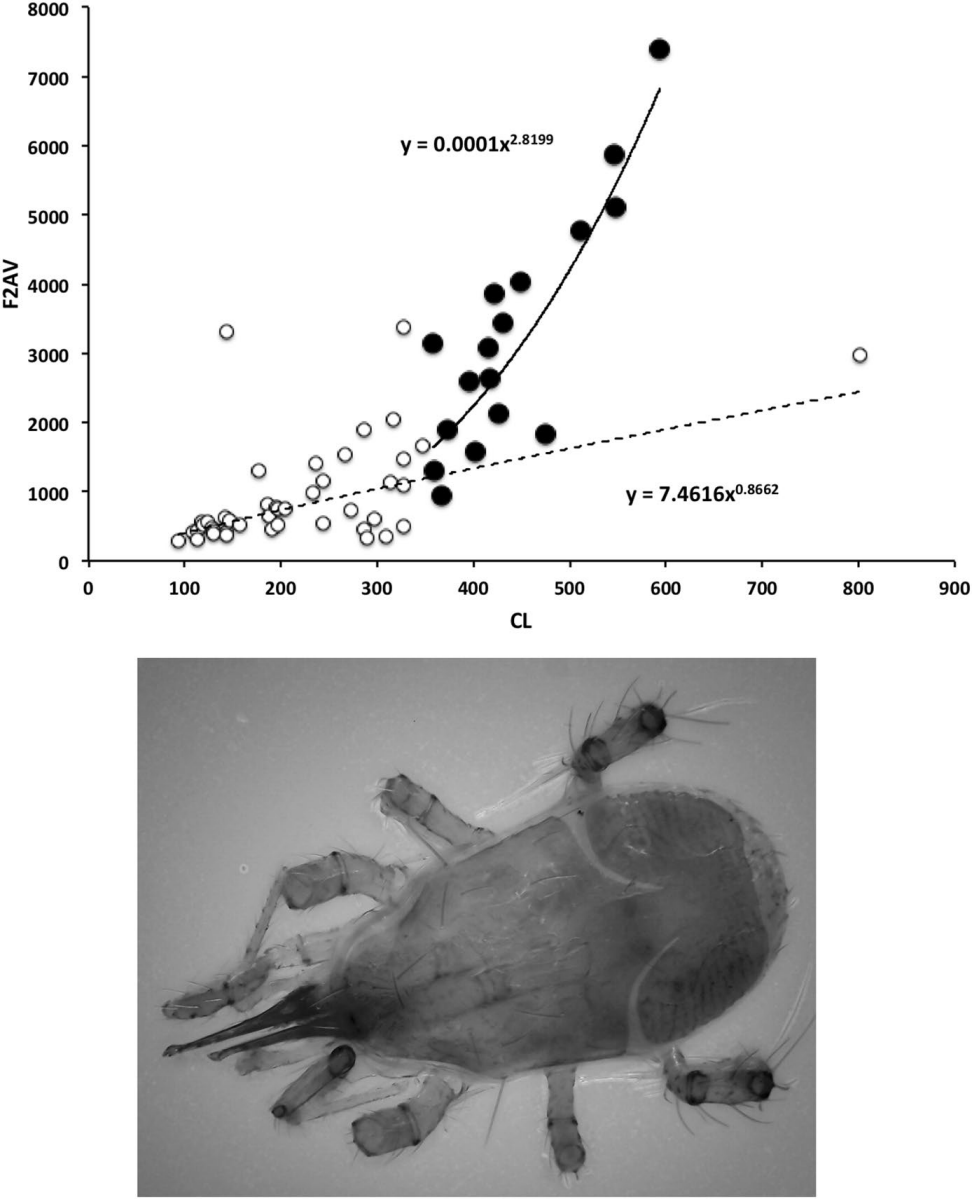
Predatory mesostigmatids are like other arachnids. *Upper* Large reach mites in black (with the exception of *Veigaia cerva*—open circle to the right) have a scaling relationship between estimated crunch force and reach (CL) just like predatory solifugids (where their scaling exponent = 2.18–3.61 in Meijden et al. 2012b). Black solid dots and solid power trend line = mites with > 350 μm reach (CL)—note fitted exponent = 2.8199. Open circles and dotted regression line = mites with < 350 μm reach (CL) (plus *Veigaia cerva*) as best fit to lower part of overall curved relationship. *Lower* *Veigaia cerva* with its crocodile-like chelicerae. From a colour photograph by Matthew Shepherd, Soil Biodiversity UK under Creative Commons BY-NC-SA 3.0 Licence

Rather, physics and taxonomic differences by evolutionary descent might offer a more interpretable conclusion to morphological selection pressures. Non-pollen feeding phytoseiids show generalist values for their aspect ratio (Table 6) and relationships with reach and gape (Fig. 8) that suggest probably a micro-arthropod predatory design as befits their feeding habit on tetranychids, eriophyids and tarsonemids (Evans 1992). Small (absolute) reach mites if predatory, have *per force* a small attack radius (Eckhardt 1979). They should behave like active searchers widely foraging for ‘hard-to-find’ but ‘easy-to-catch’ prey (Pianka 1971). As such, coexistent species which exploit the structural diversity of the foraging micro-habitat would be very similar morphologically (Eckhardt 1979)—much as many small to medium size mesostigmatids are. Large (absolute) reach mites ($>350\,\mu \text {m}$), with the exception of *Veigaia cerva*) have a relationship with crunch force almost exactly like that of predatory solifugids—Fig. 9. This is consilient with solifugids being a sister-group of mites based upon their mouthparts (and also perhaps their reproductive characters—Dunlop and Alberti 2008). *Veigaia cervus* has an unusual massive chelicera with the fixed digit considerably longer than the moveable digit (Evans 1955)—perhaps deployed as a spear against prey? Do ricinuleid chelicerae work like solifugids too (Tuxen 1974)? The observed scaling exponent for the mesostigmatids is 2.82 - right in the middle of 2.18–3.61 in solifugids (Meijden et al. 2012b).

**Fig. 10:**
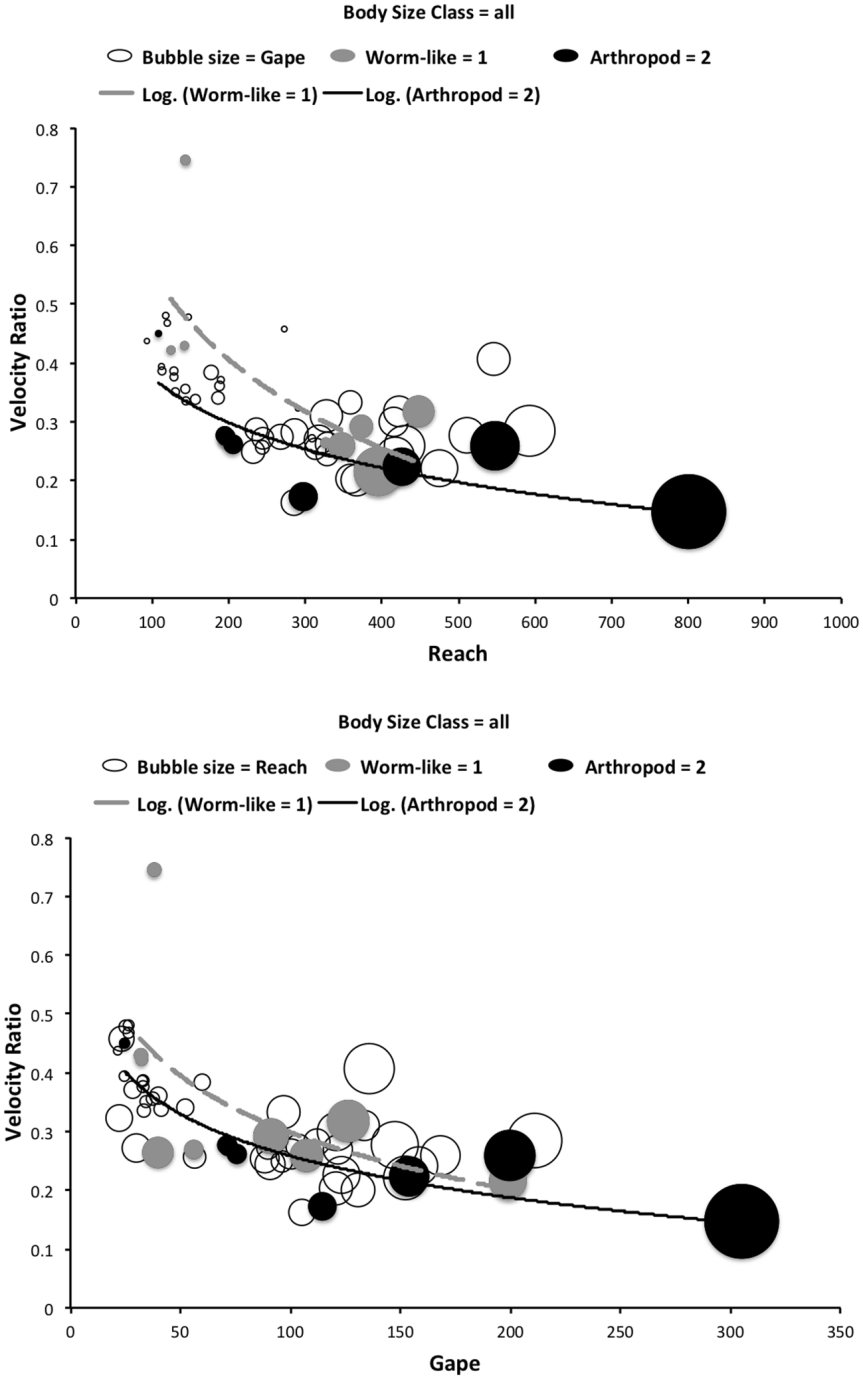
Paying attention to small organisms is illuminating. Plot of chelal velocity ratio ($\frac{L1}{L2}$) for mesostigmatids (this study + from Buryn and Brandl 1992) versus chelal gape (MDL) or cheliceral reach (CL). Log regression lines (*simply for illustration*) fitted to known feeding preferences (grey dashed = worm-like prey; black solid = microarthropod prey). Note lines join asymptotically at large mite sizes. *Upper* Against reach (CL) with gape (MDL) as bubble size. *Lower* Against gape (MDL) with reach (CL) as bubble size

In general, rapid movements are effected in arthropods by relatively longer muscles giving greater displacements (Manton 1958a)—longer chelal muscles require longer chelicerae! Only short reach mites (< 350 μm)—which contain half the uropodine species examined - have strongly elevated velocity ratios (Fig. 10*Upper*). Higher velocity ratios are also usually found for small gape species (< 150 μm—Fig. 10*Lower*)—*per force* only small prey can be killed with a crushing/mashing/chewing action. The small omnivorous species from Buryn and Brandl (1992) do show an increased velocity ratio (Fig. 7*Lower*). A plot of F2AV versus gape (MDL) shows similar dual slope behaviour to Fig. 9 using a threshold of 150 μm (*results not shown*). So, again one should pay attention to small organisms (Paine 1996).

**Fig. 11:**
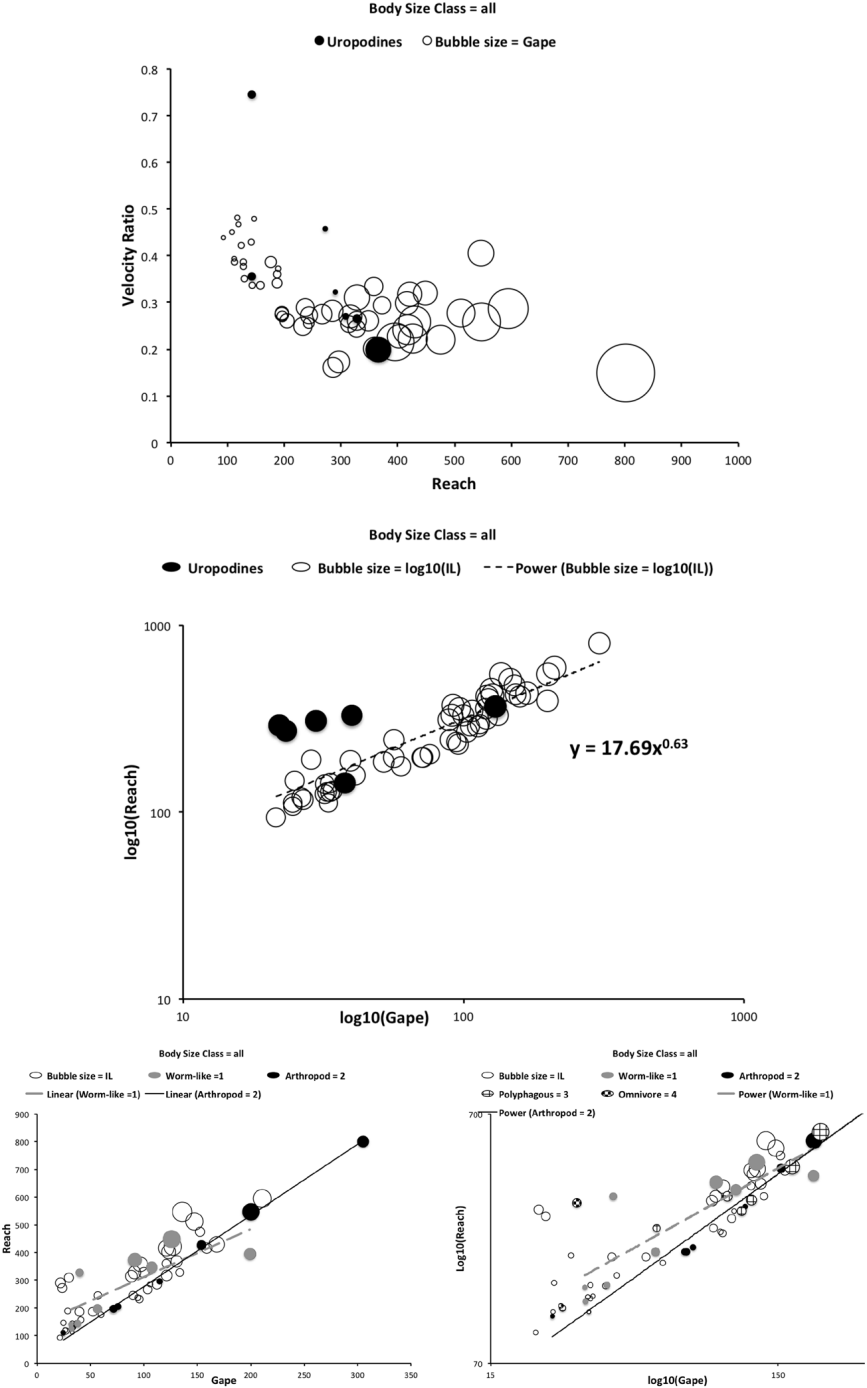
Small organisms are illuminating especially uropodines in the space of all the mesostigmatid data (this study + from Buryn and Brandl 1992). *Upper* Plot of chelal velocity ratio ($VR=\frac{L1}{L2}$) versus reach (CL) for mesostigmatids. Black solid circles = uropodines. Bubble size is estimated chelal crunch size (F2AV). Note high velocity ratios for small reach and gape uropodines. *Middle* Plot of reach (CL) versus gape (MDL) and common allometric regression line. Black solid circles = uropodines. Bubble size is log body size (IL). Note subset with a much bigger reach than the overall allometric relationship would suggest for that chelal gape (or alternatively a much smaller gape for a similar reach to other mesostigmatids). *Lower* Plot of gape (MDL) versus reach (CL) with separate regressions lines for food type. Here, grey circles and grey dashed line = worm-like prey feeding; black circles and black solid line = microarthropod feeding. Linear regression lines (*simply for illustration*). *Left*: Arithmetic scales. Note how uropodines drag the worm-like feeding regression upwards. *Right* Log–log scales blown up at low x-axis (gape) values. Polyphagous species (criss-cross open square pattern filled circles) plot in and amongst worm-like prey and micro-arthropod feeders. Omnivore circles are filled with spotted pattern. The uropodine omnivore *Trachytes aegrota* plots in the isolated subgroup

Small gape species may be nibblers like the fish *Leporinus*; Alexander (1964). Small reach mites might be surface feeders (cf. gleaners) like the fish *Pyrrhulina*; Alexander (1964). Small mites also have the design space opportunity to not just be predator-scavengers but to be frequently non-predators consuming very hard (sessile or sedentary) fungal substances through having very large velocity ratio (VR) value chelal designs. A possible example of this is the worm-like prey feeder *Alliphis siculus* in the subset of the uropodines in Fig. 11*Upper*, that includes species = *Alliphis halleri*, *Urodiaspis tecta* and *Uropoda orbicularis*. These mites often have a disproportionately large reach (beyond simple allometry based upon their gape—see Fig. 11*Middle*). This agrees with observations in Evans (1992) and suggests that these are adapted for accessing and chewing morsels of food in crevices or at least at long distance from their bodies (like *Eviphis ostrinus*, the omnivore *Trachytes aegrota*, *Urodiaspis tecta* and *Uropoda orbicularis*). Hypothesis *(ii)* is supported. Nematodes are very slender and soft-bodied being able to enter very small soil-pore spaces unavailable to the mostly broader and less flexible mites (Walter and Proctor 2013—exceptions here could be the deep dwelling rhodacarids with their particular idiosomal architecture?). Given that nematodes need at least a film of water to move around (if not water-filled pores that the air-pore requiring mites cannot access), an extensive reach would be a very useful predatory adaptation for a small mite to access nematode refugia. This also fits in with the claim of Willis and Axtell (1967) that the long chelicerae in the small cryptognathic uropod *Fuscuropoda vegetans* is an adaptation to penetrate into prey. By withdrawing such long chelicerae deep back into their idiosoma, such mites are then showing similar adaptations to parasitoid flies (Gilbert and Jervis (1998)) with their “concealed...extraction apparatus”. Alternatively, this latter subset of uropodines which have a much smaller gape for a similar reach to other mesostigmatids, may specialise in feeding on food morsels at a fragment of their overall scale. They would then be micro-food browsers or grazers like vertebrate ungulates eating grass. Hypothesis *(iii)* is supported.

Illustrations and figures for moveable digit length and height in Athias-Binche (1977) suggest that *Cilliba cassidea* and *Polyaspis patavinus* are likely to have similar velocity ratios around 0.38 and 0.36, respectively (despite their differences in approximate aspect ratios of 5.0 and 6.9, respectively, to the uropodids studied herein). *Cilliba cassidea* was the only edaphic uropod that was phytophagous in the beech forest feeding upon unicellular green algae principally Chlorococcales (Athias-Binche 1977). It would be worth studying a larger number of other uropodoids (especially small body size species) to look for distinctly different cheliceral forms marking them out to have a different basal shape than non-uropodoids i.e., their trophic design differences might be of taxonomic origin rather than of the same functional class as other gamasiform mesostigmatids. Some uropodiforms do appear to have a (third) baso-basal cheliceral segment (not used in this study) appearing thus 4-segmented (see Van der Hammen 1970a for the mapping to traditional arthropod segment names). Could this extra segment be contributing to an even larger crunch force needed to crack intractable material, or indicate a different modality of cheliceral use? Despite this type of uropodine possibly skewing the apparent form of worm-like prey feeding designs at small sizes in the data (Fig. 11*Lower Left*), where the polyphagous mites plot in general is still consilient with Buryn and Brandl (1992)’s conclusions (Fig. 11*Lower Right*), so for this study the overall bias at moderate to large body sizes is low.

Excessive gape has different corollaries.

Firstly one would expect a concentration of moveable digit mass in the condylar area so as to keep its moment of inertia low and thus the initial force to start it moving low (Alexander 1968). In that way large mites with elongate digits should have a propensity to be ‘cutters’ and ‘slicers’ not ‘crushers’. Their digits should get more and more slender distally (consider the tip of a kitchen knife for instance).

Secondly there is the opportunity for a series of slicing bites over a long parts of the prey’s body. A lengthening of the blade allows a longer cut for a given bite. Moreover in situations facing vertebrates carnivores (Van Valkenburgh 2007) where rapid ingestion is favoured (such as between litter-mates or adults feeding together on a kill), selection should favour the evolution of a longer blade and greater bite force. Free-living mesostigmatids ingest very rapidly (Bowman 2019) so a pressure for this change probably exists similarly. Note that active consumption rates increases with sociality in carnivorous vertebrates (Wilmers and Stahler 2002). Walter and Proctor (2013) on page 112 illustrates social feeding of *Macrocheles superbus* on an oligochaete worm. De Gasperin and Kilner (2015) illustrates two *Poecilochirus carabi* mites feeding upon the same first instar burying beetle larva *Nicrophorus vespilloides*. The uropodid *Fuscuropoda vegetans* is a gregarious feeder of 1st instar house-fly larvae (Willis and Axtell 1968). How much of all of this is by group hunting (Usher and Davis 1983) versus just group feeding (Blaszak et al. 1990; Seeman and Walter 1997; De Gasperin and Kilner 2015) remains to be seen.

Thirdly, as in canids, a mite could hang onto a large fraction of the prey and shake it (through idiosomal movement) or even bodily flip it over. The thrashing of large nematodes is known to dislodge and fling off attacking mites (Walter and Ikonen 1989). *Veigaia* species are a taxonomic group showing particular large gape values (Table 3). *Veigaia* spp. are deliberate in their searching and often pause to wait for prey (Walter and Proctor 2013). Watching veigaids in the act of feeding could be very illuminating. Large gape mites need to quickly snap their chelal jaw shut if they are to successfully catch and feed on vagile prey, and then keep their chelae shut given that the active prey may be wriggling to escape. How are these two issues handled? Does the inertia of a large idiosoma prevent these mites being ’thrown around’?

**Table 7:** Large gape mesostigmatids from Acarology Laboratory, Ohio State University museum slide collection

Species	IL (μm)	MDL (μm)	Tips cross	Catch or ’pocket’	Levator tendon
*Arctacarus rostratus* $\female$	779.1	158.6	+	−	Pale
*Arctacarus rostratus* $\female$	832.1	156.0	+	−	Pale
*Arctacarus rostratus* $\female$	1028.2	197.6	+	−	Pale
*Gamasolaelaps* sp. $\female$	477.7	48.1	(+)	−	Pale/strong
*Gamasolaelaps* sp. $\female$	424.0	45.5	(+)	−	Pale/strong
*Veigaia pusilla* $\female$	376.3**	109.2	+	+	Pale
*Veigaia mitis* $\female$	466.4	58.5	(+)?	(+)	Strong/chitinised
*Veigaia exigua* $\female$	466.4	101.4	+	+	Pale/strong
*Veigaia alba* $\female$	503.5	114.4*	+	−	Pale
*Veigaia tranisalae* $\female$	630.7	92.3	(+)	(+)	Pale/strong
*Veigaia* n.sp. nr. *sibirnica* $\female$	651.9	148.2	+	(+)	Chitinised
*Veigaia planicola* $\female$	662.5	140.4	+	(+)	Chitinised
*Veigaia nemorensis* $\female$^a^	678.4	152.4	+	+	Chitinised
*Veigaia partitus* $\female$	699.6	109.2	+	+	Strong/chitinised
*Veigaia cerva* DN	704.9	163.8	+	+	Pale/strong/chitinised
*Veigaia cerva* $\female$	879.8	179.4	+	+	Pale/strong/chitinised
*Veigaia nodosa* $\female$	916.9***	174.2	+	-	Chitinised

IL measured as in this study. L2 and L1 not possible to measure unequivocally. Multiple categorisations for levator tendon indicate changes along its length to allow local bending/flexibility. NB. Only short chitinised sections are attached to moveable digit at top of L1. Sorted by size (IL) within genus. Note mild correlation of levator tendon strengthening with body scale over *Gamasolaelaps* and other veigaids (prevention of stretching on high-speed chelal closing?). Note also mild correlation of ‘catch’ or pocket in tips of moveable and fixed digit so as to fit together (Hurlbutt 1968), and propensity for tips of both to cross-over when closed (see Fig. 13) with increasing MDL (gape) over *Gamasolaelaps* and veigaids

^a^Mean from the extra *Veigaia nemorensis* mites used in this study (the tendons in all five mites examined were chitinised). *MDL in Hurlbutt (1965) = 110–115 μm. **Body size in Hurlbutt (1968) = 380 μm. ***Body size in Hurlbutt (1968) = 840 μm

**Fig. 12:**
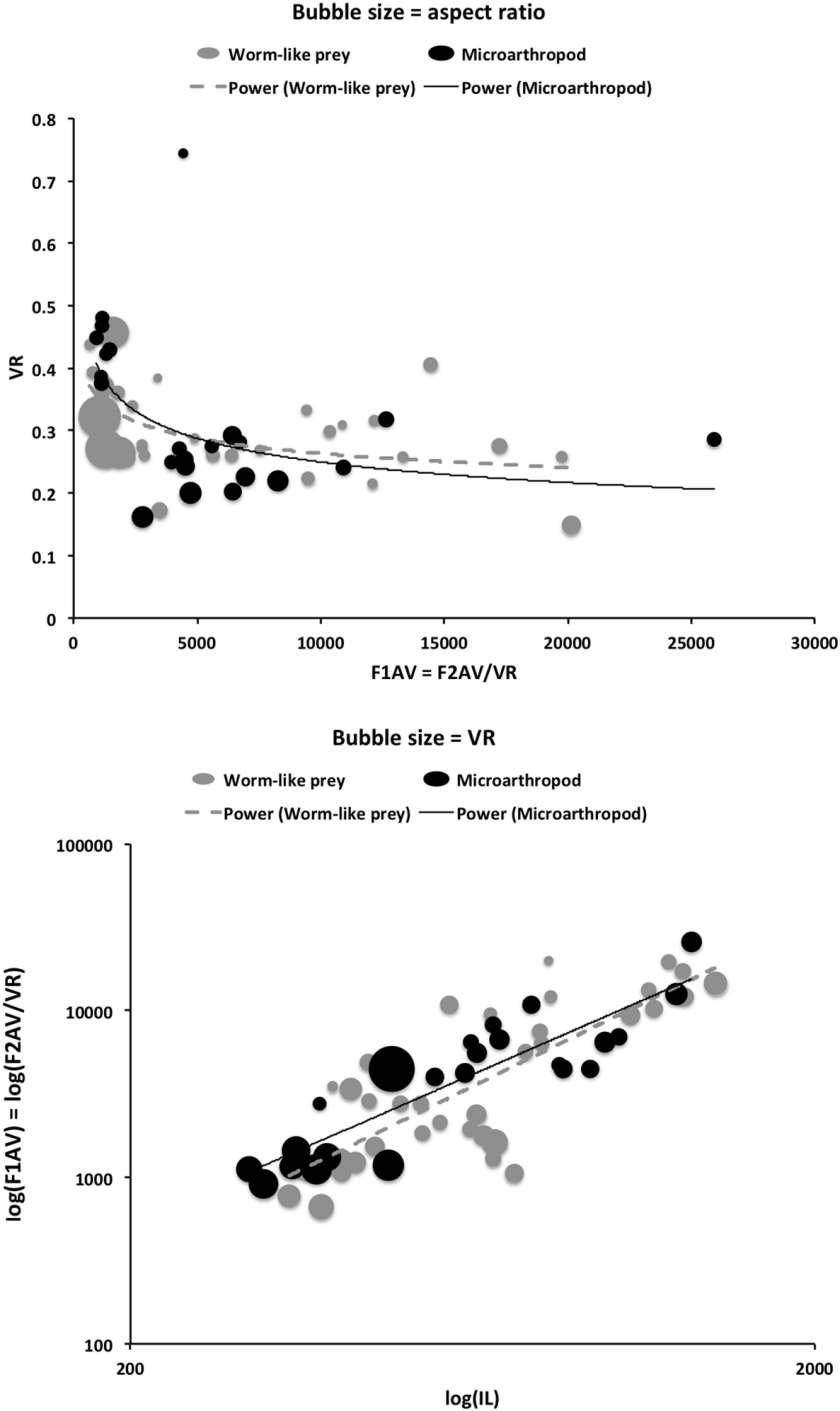
Focusing on small mites is insightful. Relationships of primary adductive force along chelal levator tendon (F1AV=F2AV/VR). Black circles and black solid regression line = predicted microarthropod feeder. Grey circles and grey dotted line predicted worm-like prey feeders. Note separate regressions lines are essentially the same. *Upper* With chelal velocity ratio (VR) and bubble size as cheliceral aspect ratio (CL/average(HBS,HDS)). Only markedly large aspect ratios are found in mites with small F1 and often high VR (cf. uropodines with elongate chelicerae). *Lower* With size (IL) on log log scale and bubble size = velocity ratio. Larger mites have allometrically larger F1 force on levator tendon (as in brachyuran crab chelae—Schenk and Wainwright 2001). However, mites at any one size which feed upon different prey have different chelal designs not different primary levator forces (F1)

Table 7 shows that larger body size mites (*Gamasolaelaps* and veigaids), and therefore those with a larger expected F1 force on the chelal levator tendon (Fig. 12*Lower*), have progressively strengthened tendons. Evans (1972) claims some of the Parasitidae have this too. From a basal state of being thin and pale in cleared and permanently mounted specimens at small sizes the chelal levator tendons are strongly thickened and eventually chitinised as the mite species get bigger. This is to be expected to prevent elasticity or viscoelasticity causing the tendon to elongate under large or fast accelerating loads from the cheliceral muscles on chelal closure—requiring hyper-shortening of muscle fibres; or, the tendon unwontedly storing kinetic energy (birds ossify their digital flexor muscle tendons similarly to prevent this—Alexander et al. 1979). This is a particular issue for any large cheliceral length species, as a given adductive force will store more strain energy in a long tendon than a short one. A given moment around the chelal condyle will require a larger force on the tendon if the moment arm of the tendon (i.e., L1) is short rather than if it is long, so since strain energy is proportional to the square of the force—a short input lever moment arm (L1, as in low velocity ratio species) implies a large strain energy. However, a thick tendon will stretch less than a slender one and so store less strain energy for a given force. So in comparison to other mesostigmatids, the much greater thickness/strengthening of the levator tendons in most veigaids compensates for their greater moveable digit tendon length (inferred by assuming larger CL for larger IL in Table 7) and for a smaller moment arm around the condyle (i.e., low VR; Tables 3, 8). Cheliceral closure driven by a strengthened tendon also does not suffer from any lag compared to the time of muscle fibre contraction so any chelal ‘snap’ is effectively instant.

**Table 8:** Prey type for mites predicted from *glm* model $logit(p(wormlike\ feeding))=f(log(sin^{-1}(\sqrt{Velocity\ ratio})))$ on test data set including estimated probability of classification (${\hat{p}}$) as a worm-like prey feeder

Species	Velocity ratio	Predicted* feeding type	${\hat{p}}$
*Alliphis halleri*^a^	0.357	Worm-like prey ✓	0.569
*Amblyseius okanagensis*	0.385	Worm-like prey	0.597
*Ameroseius* sp.	0.438	Worm-like prey	0.644
*Androlaelaps casalis*	0.361	Worm-like prey	0.574
*Arctoseius brevicheles*	0.478	Worm-like prey	0.675
*Arctoseius minutus*	0.394	Worm-like prey	0.606
*Arctoseius venustulus*	0.336	Worm-like prey	0.547
*Eugamasus berlesei*	0.317	Worm-like prey	0.526
*Eugamasus cavernicola*	0.299	Worm-like prey	0.504
*Glyphtholaspis confusa*^a^	0.406	Worm-like prey ✓	0.616
*Iphidozercon gibbus*	0.371	Worm-like prey	0.584
*Leioseius bicolor*	0.351	Worm-like prey	0.564
*Pachylaelaps furcifer*	0.243	Microarthropod prey	0.431
*Pachylaelaps leauchlii*	0.254	Microarthropod prey	0.447
*Parasitus beta*	0.340	Worm-like prey	0.552
*Parasitus coleoptratorum*^a^	0.276	Worm-like prey ✓	0.476
*Parasitus fucorum* ^a^	0.334	Worm-like prey ✛	0.545
*Parasitus lunaris*^a^	0.202	Microarthropod prey	0.369
*Pergamasus cornutus*	0.272	Microarthropod prey	0.471
*Pergamasus mirabilis*	0.241	Microarthropod prey	0.429
*Pergamasus oxygynelloides*	0.249	Microarthropod prey	0.439
*Pergamasus* sp.	0.275	Microarthropod prey	0.474
*Polyaspis* n.sp.^a^	0.199	Microarthropod prey ✗	0.365
*Porrhostaspis lunulata*	0.227	Microarthropod prey	0.407
*Rhodacarellus epigynalis*	0.385	Worm-like prey	0.597
*Rhodacarus agrestis*	0.288	Worm-like prey	0.491
*Rhodacarus strenzkei*	0.310	Worm-like prey	0.518
*Typhlodromus setubali*	0.450	Worm-like prey	0.645
*Urodiaspis tecta*	0.322	Worm-like prey	0.532
*Uropoda orbicularis*^a^	0.458	Worm-like prey ✓	0.660
*Veigaia decurtata*	0.161	Microarthropod prey	0.301
*Veigaia nemorensis* (new)^a^	0.220	Microarthropod prey ✓	0.397
*Zercon peliatus*	0.337	Worm-like prey	0.548

*Velocity ratio (VR) threshold = 0.276. Uses species of unknown food type from Buryn and Brandl (1992) plus

^a^ = extra species added in this study (these then marked ✓ if agrees with literature, ✗ if not agree with literature, ✛ ambiguous literature). See text for discussion of *Polyaspis*

Critical mechanisms to an animal’s life are likely to have larger safety factors against failures, with large load structures usually disproportionately strengthened (Taylor et al. 2000). So a study of cheliceral length (and thus likely cheliceral attack radius) versus tendon strengthening for different body size veigaid species which coexist as prey “snap-trappers” (*sensu *Walter and Proctor 2013) in the same habitat would be useful. Is this similar in other mites with large moveable digit L2 values like: the oligophagous (Evans 1979) predator *Parholaspella spatulata* (with a possibly strong levator tendon—Krantz 1971), or *Parholaspulus lobatus*? Which parasitids is it that Evans (1979) states have strongly sclerotised moveable digit levator tendons? Does *Artacarus rosatratus* have a short cheliceral shaft and low F1 closing force obviating needing any tendon reinforcement? What is the situation in *Geholaspis (Longicheles) mandibularis* with its very large moveable digit length (Fig. 27-7 in Krantz 1978)? One would expect passive searching species to differ (when ordered in increasing size) by more than a morphological ratio of > 1.4 (Eckhardt 1979). Is this true or not of these large gape, large reach species? More morphological work is needed.

**Fig. 13:**
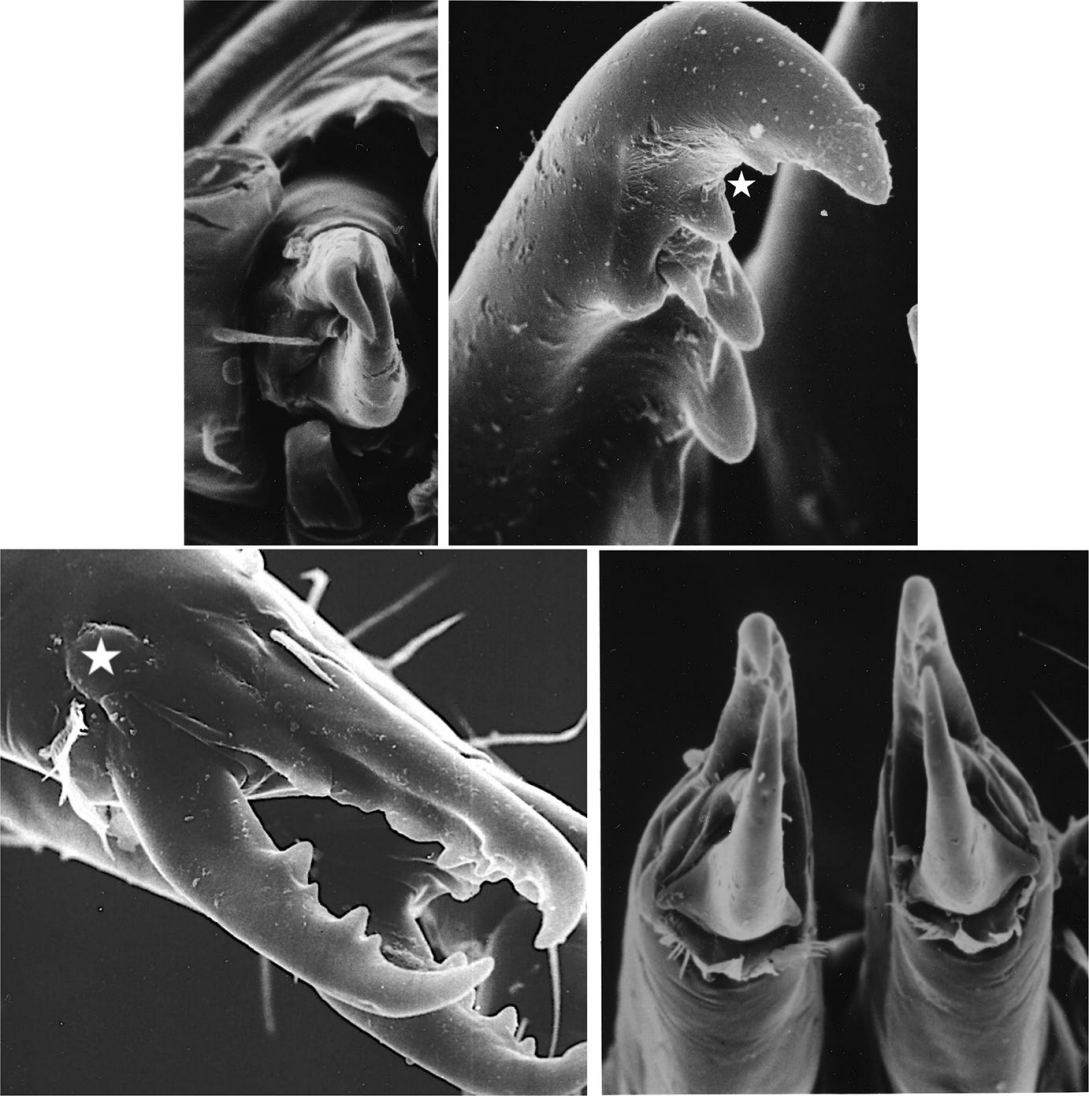
Scanning Electron Microscope pictures of different parasitid mite mouthparts showing various mechanical features. *Upper* *Left* Cheliceral chela end-on with overlapping ‘locking’ digit tips (like in pseudoscorpion chelicerae and pseudoscorpion pedipalp chelae). Parasitid palp truncated for clarity. Note peaked gnathotectum, ventrally a corniculus that slides into face of moveable digit basally and a salivary stylet running along digit teeth area. Excess prey fluids would form a cylinder of liquid between the palps that the chelicerae pass through—Bowman (1984). *Right* ‘Pocket’ in tip of fixed digit into which tip of moveable digit locks (shot from below looking upwards dorsally). The moveable digit tip swings through the location indicated by the small white star and ‘locks’ where the digit chitinous surface is wrinkled between the small teeth in a ‘gate catch’-like assembly. *Lower* *Left* Exterior of condyle marked by large white star. Note swelling and associated lyrifissure. *Right* Offset moveable digit condyles—not strictly orthogonal to the cheliceral axis. Yaw and roll produces rotational moments on prey tissue as moveable digit closes

Gape certainly has its challenges for mites. In seemingly all veigaids, the tips of the moveable digit and fixed digit cross-over when closed—as in other large mites (Fig. 13) sometimes by virtue of offset condyles (for sure in *Parasitus coleoptratorum*). Given that mesostigmatid chelae only have one set of levator muscles (and not equivalents of both the temporalis and masseter muscles found in vertebrates; Turnbull 1970) moveable digit disarticulation/dislocation must be a risk (see discussion in Smith 1981). A small ‘gate catch’-like assembly formed by an ‘open pocket’ and ‘mini-tooth’ on the fixed digit that ’captures’ the moveable digit tip is seen on the digits (Fig. 13) so as to lock them together on chelal closure as the length of the moveable digit MDL increases in this large-gape species series (see Table 7). All of this increases stability when the chela is closed and under pressure to be prised open by cause of prey strugglings. The orientation of this mechanism, axial or abaxial, varies with species. Why is that? One possibility is that a different choice has been made in the muscle packaging arrangement within the cheliceral shaft. If the condyles are offset then there is the opportunity when the moveable digit opens or the chela closes that the levator and depressor muscles can be so arranged not to interfere with each other as they act. Crossed-over tips when the digits were closed would also effectively widen the wound in any prey on cheliceral retraction (consider the beak action of crossbill birds).

Body size in itself may also be important in further work looking at say more species with reduced fixed digits, since any prey punching/piercing by using body weight leverage alone obviates the need for great musculature in chelicerae especially if the latter are small and of low volume. So stylet feeding should be a clear mesostigmatid subgroup in any trophic design ordination based upon size, reach, gape and estimated chelal crunch force. Intuitively stylet feeding ought to be found in small to moderate (but not large) sized predatory mesostigmatids and should facilitate possible pollen, spore or very small egg feeding. The results from Adar et al. (2012) and Liu et al. (2017) using their DPFD/VPMD measure suggests at least facultative plant tissue stabbing in phytoseiids. In fact, Flechtmann and McMurtry (1996) discuss that phytoseiids may be more like host-piercing and fluid-extracting parasites than true carnivores. How such designs might lead onto specialised fluid feeding in properly parasitic mesostigmatids that stab tissues (Treat 1955; Troitskii 1973) needs further work. Nevertheless reach and gape is important (as indirectly already indicated by Buryn and Brandl 1992).

### Is the initial force on the levator tendon (F1AV) important?

There appears to be no relationship of primary adductive force upon the levator tendon ($\text {F1AV}=\text {F2AV/VR}$) with feeding style across mesostigmatids in general. Only at small sizes is there gross differentiation into “hard crushers” versus (relatively) “speedy closers”. Figure 12 shows that a common power relationship would fit both predicted microarthropod feeder habit as well as the worm-like prey feeder habit. This is unlike the distinction between primary decomposer and secondary decomposer oribatids in Perdomo et al. (2012) on the basis of their estimated levator cross-sectional area. Predatory mesostigmatids appear to adjust their crunch force to different prey needs rather by changing the mechanical advantage of their chelal jaw lever system (i.e., as in the *glm* model) and not by the differential design of the cheliceral shaft for more or fewer muscles. Size just moves the mites along into a different scale class. Only small size allows any obvious further differentiation in gape (at values < 150 μm) and reach (at values < 350 μm). Small mites are illuminating!

### Is the cheliceral aspect ratio important?

Regarding cheliceral aspect ratio—recall that herein the third principle component (PC3—interpreted possibly as an ‘aspect ratio’ contrast of MDL versus HDS) was found to be useful in the analysis of the augmented data set above. Low aspect ratios indicate a cursorial life style at least in predatory solifugids; Meijden et al. (2012b). The top 10 most extreme aspect ratios (in order of increasing value) were: *Veigaia nemorensis* (new)—6.95; *Veigaia decurtata*; *Cheiroseius borealis*; *Polyaspis* n.sp.; *Veigaia cerva*; *Iphidozercon gibbus*; *Uropoda orbicularis*; *Eviphis ostrinus*; *Trachytes aegrota*; *Urodiaspis tecta*—17.46 (Table 3). This covers five out of the seven uropodines studied. No relationship of cheliceral aspect ratio (= CL/Average(HBS,HDS)) with predicted prey feeding habit (microarthropod versus worm-like prey) was found (*result not shown*). *Veigaia nemorensis*, *Veigaia cerva* and *Veigaia decurtata* look like specialist ambush ‘sit-and-wait’ predators of ‘hard-to-catch’ soft-body collembola (i.e., ‘passive searchers’ like desert lizards; Pianka 1971). However, whether in the act of grabbing the moveable digit swings vertically (assumed likely for an arachnid), or behaviourally the whole gnathosoma is tilted and the moveable digit swings horizontally like an open bear-trap, needs *in vivo* observations in these species. The two ascids *Cheiroseius borealis* and *Iphidozercon gibbus* plus the known predator *Polyaspis* n.sp. may be prey pursuers foraging over a large space (Eckhardt 1979). Are other ascids (like *Maxinia*, Lindquist and Makarova 2012),  designed similarly?

In cryptostigmatids, high aspect ratio values engendered by a pelopsiform style to the chelicera (e.g., drawing 5 in Bayartogtokh et al. 2018) is associated with fungus feeding. *Eupelops* and *Peloptulus* have this form but not *Propelops* (Seniczaka et al. 2015). Is there a high aspect ratio in phoretic *Hoploseius* spp. (Blattisociidae) who apparently use their chelicerae as scrapers to remove sporophores (Lindquist 1963)? The four uropodines have particularly markedly high cheliceral aspect ratios (> 12) for their size. Gorirossi-Bourdeau (1997) illustrates elongate chelicerae for the common *Uropoda berlesiana*. This might indicate a possible burrowing habit, although the ability of such mites to form or modify pore structures is sporadic over species (see references in Walter and Proctor 2013). Rodents are known to show concerted changes in oral morphology for a fossorial habit (Álvarez et al. 2011), with skull forms related to the hardness of the substrate (Courant et al. 1997). Are mesostigmatids like this? Perhaps some uropodines construct tunnels into the substrate accessing fungi or their prey (Willis and Axtell 1967)—using their chelicerae and chelae like a bulldozer or long-armed construction-site mechanical digger with a small distal ‘bucket’? Within such borings do mites with an elongate gnathotectum use it in turn to ‘hoe’ or like a garden rake, to disturb, drag and sort material before the chelicerae pick stuff up? It seems unlikely (but possible—see Konstchán and Starý 2012, Fig. 10 of *Cyllibula ovalis* n.sp.) that any very elongate gnathotectum (with the hypostome) help support a narrow extended liquid cylinder arising from chelal squashing of grasped material and through which the chelicera pass during feeding. Rather, such gnatotecta may simply be an adaptation to clean along the inner surfaces of long cheliceral segments as they are retracted back into the idiosoma.

### How might uropodine chelicerae function?

**Fig. 14:**
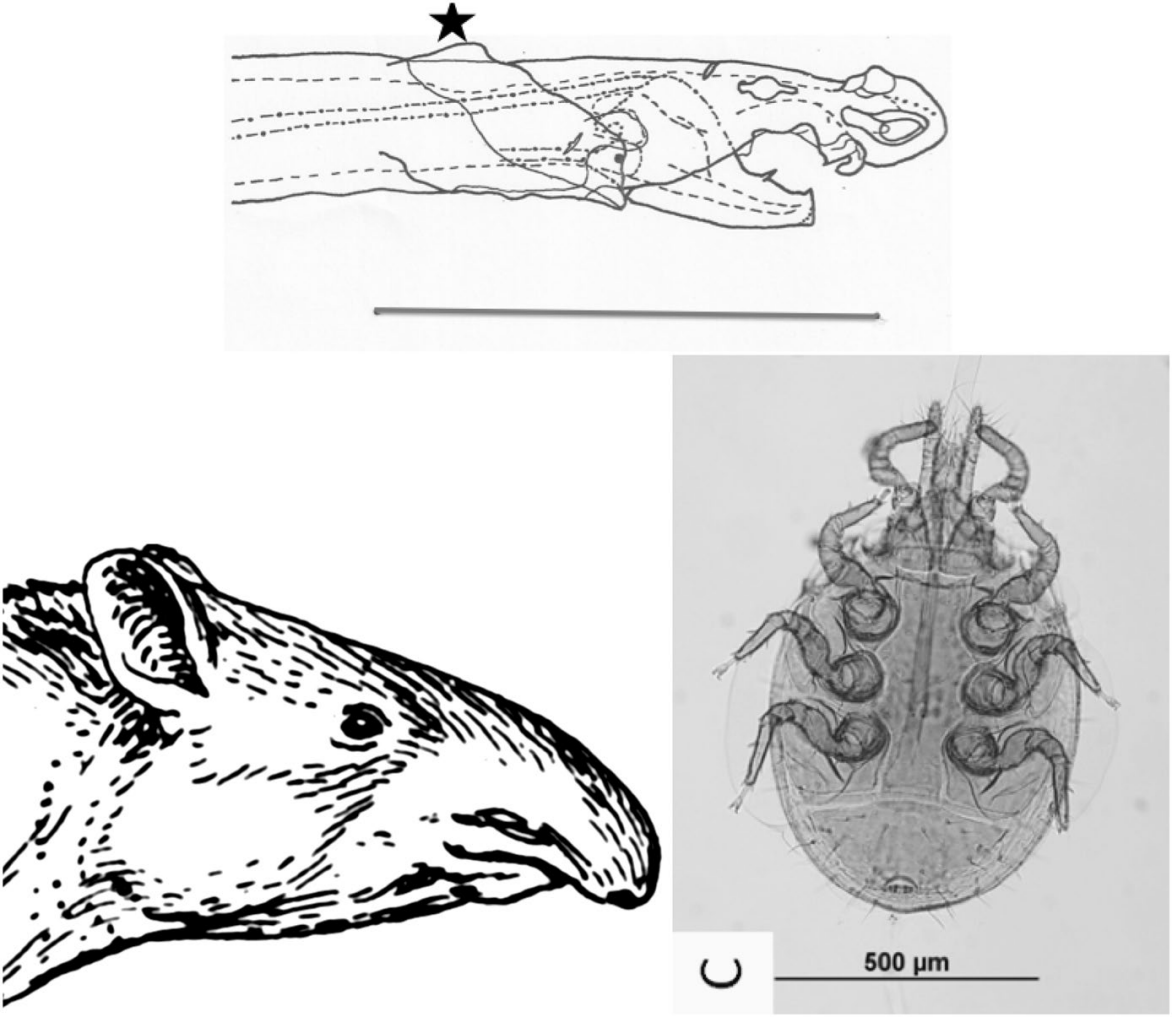
Uropodids with a flexure in cheliceral shaft and sensory apparatus distally on the fixed digit may ‘root around’ in the substrate for food like some ungulate mammals with pliable snouts (e.g., pigs, tapirids etc). *Upper* *Uropoda orbicularis* DN cheliceral chela. Grey bar is $50 \,\mu \text {m}$. Levator and depressor tendons marked as dot and dashes. Dot as condyle position. Note loose cheliceral shaft cuticle (marked with star) behind the fixed digit/moveable digit assembly with its complicated distal setae. *Lower Left* Head of tapir. Note flexible snout ahead of lower jaw. From line art representation of Tapir by Pearson Scott Foresman under Creative Commons License, see: https://upload.wikimedia.org/wikipedia/commons/e/e8/Tapir_%28PSF%29.png*Lower Right* *Uropoda orbicularis* Fig. C from Al Deeb et al. (2011) © Mohammad Ali Al-Deeb (2011) with permission. Note one very elongate chelicera withdrawn right back into body and the other extruded so far it extends off the picture. For similar in *Uroobovella marginata* see Porcelli et al. (2009)

The chelicerae of uropodines exhibit features which are rarely apparent or even absent in other mesostigmatids (Evans 1972):

1. Many have a streamlined shape like a spear with a sub-ovoid head suitable for easy insertion into food. Some have developed a distal process on the fixed digit.
2. Some have a weakening of the shaft sclerotisation between the fixed digit and its limb (Fig. 14, so as to allow extra limited movement of the chelal head?).
3. Some uropodoid species in the Dinychini, Trichouropodini, Trachyuropodini and Uroactinini tribes have a ‘Rollplatte’ (Hirschmann 1971), a nodular reinforcement on the moveable digit closing tendon (see Fig. 6d in Evans 1979). One assumes that this is non-deformable against local strong shearing forces like the lignification of wood at high stress points.
4. In some species there is an extension of the fixed digit beyond the level of the tip of the moveable digit.
5. There is also tendency in some species towards the development of inflated setae distally on the fixed digit (perhaps contact chemoreceptors?).

Taking each of these five points in turn.

**Fig. 15:**
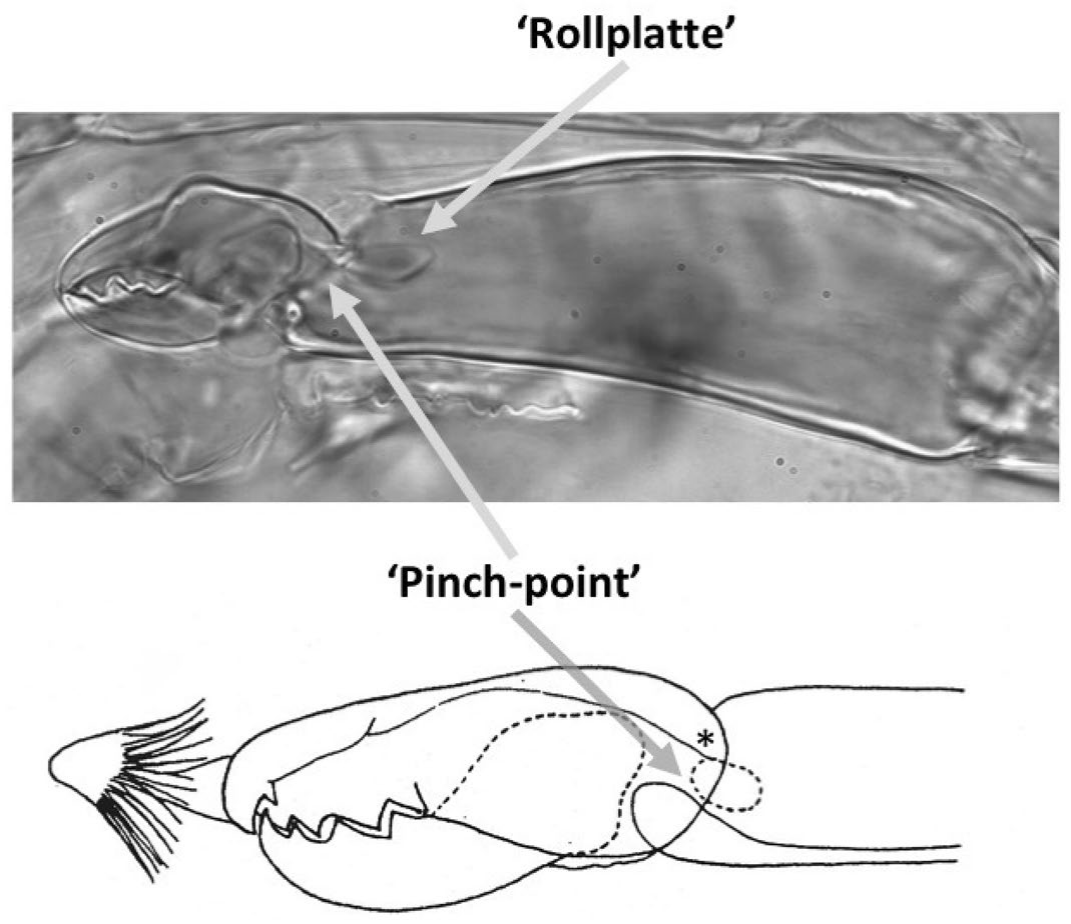
The ‘Rollplatte’ is at a “pinch-point” and may “click” in and out within the uropodoid mesostigmatid cheliceral shaft. *Upper* *Trematura nr. bassasi* DN with ‘Rollplatte’ attached to moveable digit levator tendon (pale fibre) and the “Pinch-point” inside the cheliceral shaft at chelal head—annotated from a colour photograph by Pavel Klimov, Bee Mite ID (idtools.org/id/mites/beemites) with permission. *Lower* *Uropoda brasiliensis* from Gorirossi-Bourdeau (1997) © Institut royal des Sciences naturelles de Belgique, with permission. Note distal process on fixed digit and how ‘Rollplatte’ sits at the “Pinch-point” between the ventral strengthening of cheliceral shaft and the dorsal strengthening of the fixed digit. The ventral flange of dorsal strengthening of fixed digit (*) will rub against any moving ‘Rollplatte’ (see also diagrammatic Fig. 5.12(g) in Evans 1992, and Fig. 56(c) of female *Trachyuropda coccinea* in Evans 1972). This rubbing would be particularly so if the chelal head flexes upwards

Friction against material will be higher the deeper chelicerae plunge into food. Reducing the surface area of any contact by narrowing the cheliceral shaft and streamlining structures reduces this and the necessary intra-idiosomal hydrostatic pressure for their mobilisation.The development of distal processes (1) may facilitate cleaning of the gnathosomal area on cheliceral retraction and re-protrusion during feeding—essential if they are to be repackaged back into the idiosoma (Fig. 15*Lower*). Uropodines with a brush-like Pinselpilus are good examples. As such this would match the postulated function of arthrodial brushes (Evans 1992) in some gamasines.

**Fig. 16:**
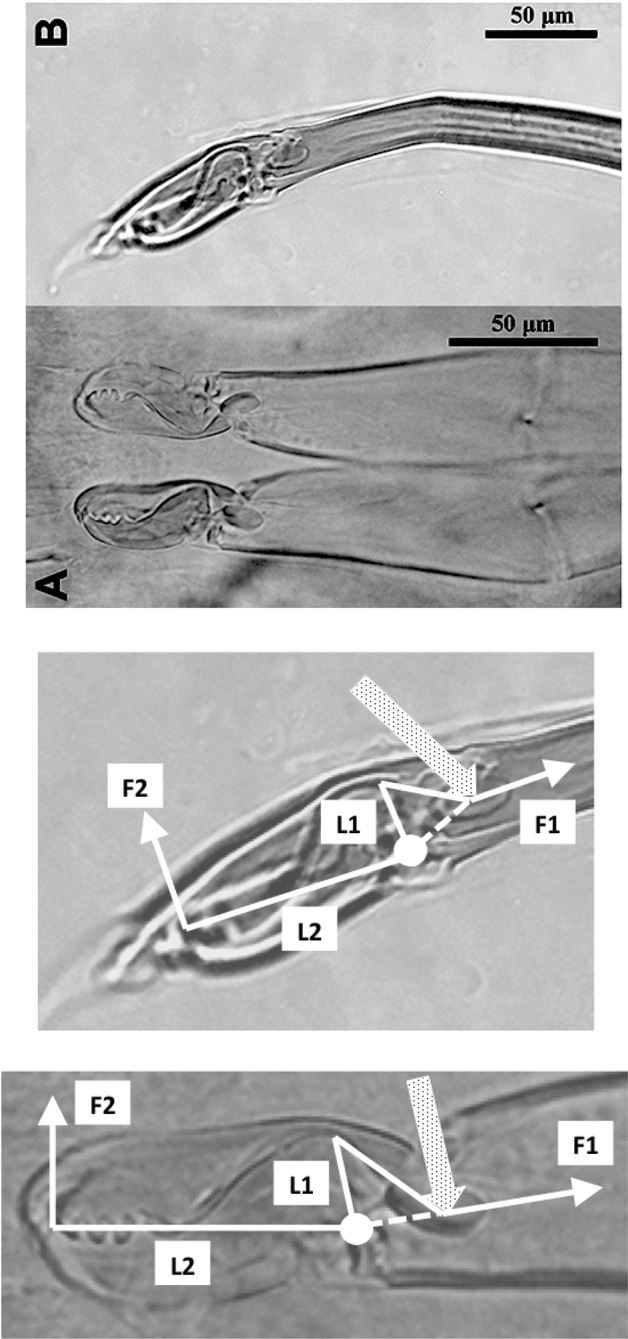
The ‘Rollplatte’ marks a potential tendon strengthening point within the cheliceral shaft of Kolbenpilus uropodids. *Upper* A = *Centrouropoda almerodai* (Uropodidae) B = *Uroobovella marginata* (Dinychidae) from Farahani et al. (2016). Contrast enhanced Fig. 5 of chelicerae © V.R. Farmahiny Farahani et al. 2016; Licensee PAGEPress, Italy; reproduced under Creative Commons Attribution Noncommercial License (by-nc 4.0) which permits any noncommercial use, distribution, and reproduction in any medium, provided the original author(s) and source are credited. *Middle and Lower* Photomicrographs annotated with lever arms and notional forces around condylar articulation (white circle). Large white arrow is downwards force against ‘Rollplatte’ caused by any chelal head flexure upwards—note it is coincident with chela shaft flexure point

Having a flexure (2) in the cheliceral shaft cuticle that buckles just behind the condyle (Fig. 14) means two things. Firstly that the whole chelal head might passively wiggle about (up and down, left and right) as the cheliceral shaft is pushed out and pulled back through any food substrate due to friction and collisions with harder chunks of material. Secondly, that on sustained pulling by the closing tendon once the chela is closed, it will ensure that the whole moveable/fixed digit assembly could flex upwards—just like a mechanical digger ‘bucket’ can move full with its contents to extricate itself from a substrate (Fig. 16). Woodring and Galbraith (1976) claimed this as one of the cheliceral movements in *Fuscuropoda agitans*. This bucket ‘wiggle’ up action is different than the origin of Evans (1979) and Evans (1992)’s unexplained statement when using *Uroactinia* as the example: “...The fixed digit in certain Uropodina is capable of limited movement that allows for the wider gape of the digits...” (see below).

If the ‘Rollplatte’ (3) is indeed associated with the adductive tendon (and not seemingly as a condylar articulation which Lopes et al. (2015) appears to illustrate in their Fig. 3A for *Oplitis*), then it would provide robustness against any stretching/rubbing by the apex of the moveable digit near its tendon attachment/insertion point whilst transiting the ‘pinch-point’ (Fig. 15) within the cheliceral shaft (for instance as the chela opens or head flexes up). In that way the underside of the cheliceral shaft above the condyle then slides over the top of the ‘Rollplatte’. Here the nodule would reduce friction on any tendon, be stabilising of the condylar complex, and redirect joint forces very much like the fabella in vertebrate knees (Dalip et al. 2018). Even if only protection from the tendon being squashed at this pinch-point by the passive movement of the chela head, this flexure protection would be advantageous (illustrations of its location in the closed chela of *Nenteria bastanii* sp.n. show it to be in exactly the right place for this; Kazemi and Abolghasemi 2016). Positioned as a point of stability and strength within the weakened sclerotisation of the base of the fixed digit it would be particularly useful when the moveable digit was maximally depressed open (Evans 1972). Could it help prevent moveable digit dislocation on prey strugglings?

Given that modern day taxonomic practice (Krantz 1978) places the ‘Rollplatte’ positive species across all the “higher uropodids” (with fovae pedales), one wonders if possession of a ‘Rollplatte’ is a safety factor (like discussed in *Veigaia* spp. above) and somehow related to cheliceral width, cheliceral length or initial adductive force and thus diet? Having a shear-resistant nodule would be an advantage then if the cheliceral tendon was pulled at a different angle to the chela head axis—such a change of direction could be the case if an elongate bendable cheliceral shaft was inserted deep into prey/food yet the chela head needing to move around independently—an advantage for a ‘benthic’ feeder.

Could it be possible that the ‘Rollplatte’ clicks in and out of the distal shaft assembly as the moveable digit closes and opens offering a ‘friction lock’ system preventing easy movement through the pinch-point (Fig. 15). This would prevent the over-extension of the levator tendon on any forced hyper-extension as the chelicerae/idiosoma moves forward with the chela jaw fully wide open digging into the food material (consider an open mechanical digger bucket being forced into the ground on digger-excavator machine movement forward). The ‘Rollplatte’ not passing through this allowing a possible cheliceral bulldozing action on any movement forwards by the mite or chelal head. Alternatively if the ‘Rollplatte’ passes beyond the pinch-point in the base of the fixed digit during maximum depression of the moveable digit, it may resist digit closing movement until any build up of continual muscular pulling on the levator tendon causes a sudden release of the ‘lock’ and an instant delivery of the increased adductive force in one nut-cracker-like ‘snap’ to chop fungal hyphae or puncture fungal spores and pollen (as Evans 1972 suggests). Such a snap would require a high tension in the levator tendon to build up which must be resisted—yet such tendons do not appear to be strongly sclerotised throughout to avoid hyperextension. To pull the ‘Rollplatte’ through such a ‘friction lock’ outwards would either require large moveable digit depressor muscles (insufficient at least in *Fuscuropoda agitans,* Woodring and Galbraith 1976) or the ability of the mite to jab the moveable digit into the prey/substrate and through cheliceral shaft or idiosomal movements prise the moveable digit open (see below).

Once the chela is closed, on even stronger pulling on the levator tendon to deliver the greatest static adductive force say when holding tough material, the ‘Rollplatte’ being pulled upwards itself might then facilitate forcing the fixed digit tip downwards (‘bucket wiggle’ down) as long as the shaft only flexes a little (see sabre-tooth action suggestion below). If the cheliceral shaft actually buckles, the tip of fixed digit rises as the chelal head rotates upwards—Fig. 16, producing a ‘bucket-wiggle’ up (as explained above for *Uropoda orbicularis* in Fig. 14). Note how the lower face of upper cheliceral shaft sclerotisation would then slide over top surface of the rising ‘Rollplatte’. On relaxation of adductive force, the natural elasticity of the cheliceral shaft chitinous integument restores the chela to a resting default position and intra-idiosomal hydrostatics or cheliceral abductors open the moveable digit again.

**Fig. 17:**
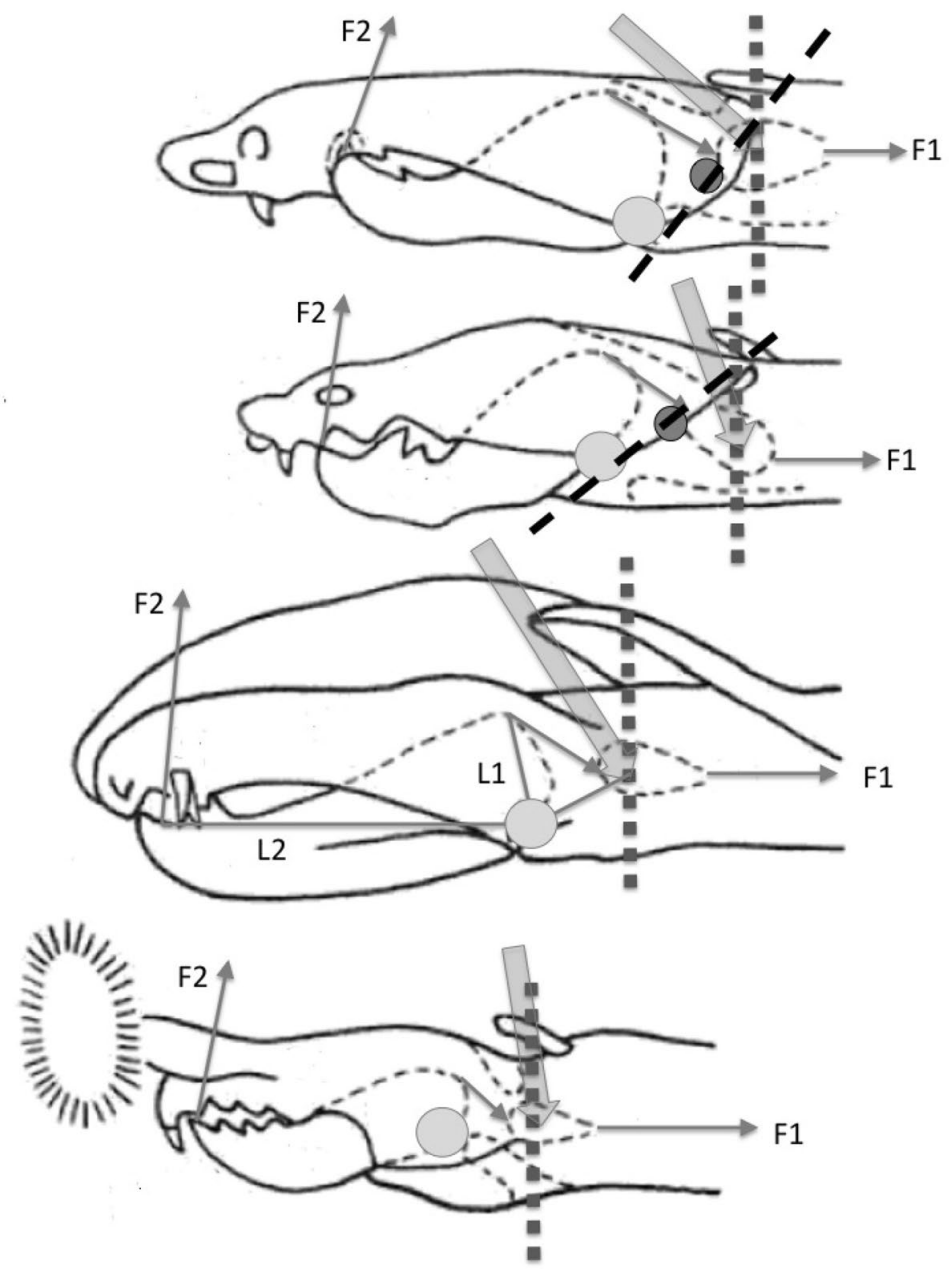
Decomposing the uropodid chelal design—statics. Examples of action of forces on a closed chela. Here uropodine chelae with ‘Rollplatte’—annotated figure based upon parts of Fig. 3 from Hirschmann (1971)). Reprinted by permission from Springer Nature ex Hirschmann W. “Gangsystematik” of the Parasitiformes and the Family Uropodidae Berlese. In: Daniel M., Rosický B. (eds) Proceedings of the 3rd International Congress of Acarology 1971. Springer, Dordrecht. © 1973. *Legend* Grey circle = approximate moveable digit condylar position. ‘Rollplatte’ = sub-ovoid dotted strengthening medial to cheliceral shaft posterior of condyle. Thin grey lines with arrows = adductive forces on chela tip (F2) and on chelal tendon through strengthened ‘Rollplatte’ section (F1). Heavy dark grey dashed line perpendicular to cheliceral shaft at maximum ‘Rollplatte’ depth = vertical section through proposed chelal head flexure point. Grey semi-transparent ‘block arrows’ = resolved force on chelal head behind condyle on continual F2 when moveable digit (locked) shut. Note the two rotation points may coincide. *Top to Bottom* First two examples = Uropodinae with Kolbenpilus (‘knobbed seta’) on ‘swollen nose’ of fixed digit. Effective rotation point for chelal head as dark circle along black dashed terminus of cheliceral shaft added for clarity. Third example down (= Oplitinae with ventral bifurcate doubled-seta Doppelpilus on fixed digit distally)—moment arms L1 and L2 labelled for clarity. Last fourth example at bottom = Uroactininae with ornate brush-like Pinselpilus on fixed digit tip

**Fig. 18:**
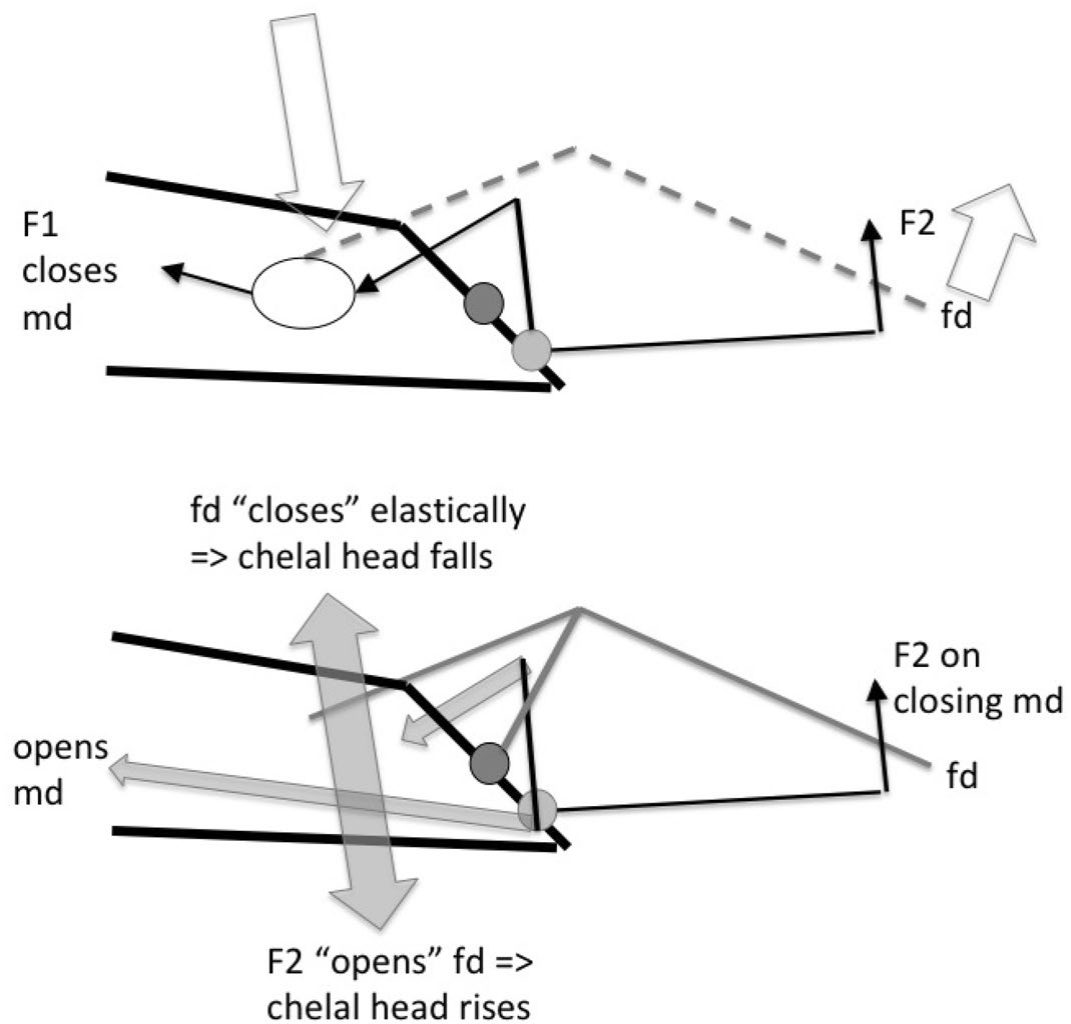
Decomposing the uropodid chelal design—statics. Mechanical digger grab-bucket analogy of *Trichuropoda columbiensis* n.sp. DN chela (taken from Fig. 17 above). Rollplatte either side of pinch-point effectively stabilises the open and closed chelal states. *Upper* Cheliceral chela. Fixed digit is the stationary toothed ‘upper bucket’. The moveable digit is the rotating toothed lower bucket. Note pale compression force at ‘Rollplatte’ induced by fixed digit tip (fd) rising on the F2 force from the moveable digit (md) and effectively rotating the chelal head ($\equiv$ large white arrows around point shown by dark circle) in order to tear off food morsels or ‘root around’ in any loose substrate. Elastic fall of chelal head could occur while moveable digit is still closed simply by the lessening of adductive force F1. Teleologically consilient with the angle of the cheliceral shaft terminus and the relative positions of rotation points. *Lower* Equivalent mechanical digger diagram. Grey thin arrows = actions of hydraulic piston equivalents. Double-headed grey arrow indicates flexure of chelal head. Note the two rotation points may coincide

Putting these suggestions all together then into an interim synthesis:

On chelal opening, the nodular reinforcement on the moveable digit closing tendon would provide the robustness against stretching or rubbing by the apex of the moveable digit near its attachment/insertion point whilst it transits the ‘pinch-point’ within the cheliceral shaft when the chela opens possibly forcing the fixed digit tip downwards. The ‘Rollplatte’ effectively clicks in and out of the distal shaft assembly as the moveable digit closes and opens offering a stabilising ‘friction lock’ system and the prevention of over-extension of the tendon on any forced hyper-extension as the chelicerae/idiosoma moves forward with the chela jaw open. If one considers that the position of the maximum depth of the ‘Rollplatte’ strengthening should be where the maximum potential stretch on the levator tendon would be, then its typical ovoid shape observed is almost exactly under where any chela head flexure should be (i.e., just in front of the sensory seta—Fig. 17). Further, one would indeed expect the biggest cross-sectional area in such an ossicle-like structure at the highest shearing stress point where it is squashed or crimped, and this is what is observed across various uropodoids. The seta just posterior of it would then be the feedback mechanism for detecting the chela head flexure upwards. This digger analogy is shown in Fig. 18. Note the two rotation points may coincide. Moveable digit closure clicks the ‘Rollplatte’ back into the distal shaft and the chelal head returns to its normal position.

Now consider, if the chelicera is embedded into the prey or substrate with the chela closed, perhaps uropodids discover desired food material using the same “zirkeln” behaviour as in corvids (see Goodwin 1976). Such birds forcibly open their inserted bill accompanied if necessary by upward and forward heaves. This “open-billed probing” might be the reason for any significant moveable digit depressor musculature in uropodids, and any dorsal strengthening of a streamlined fixed digit?

In the first instance, it is not at clear by what mechanism Evans (1992 p. 151) claims that some uropodids (*Uroactinia*) provide a wider gape of the chela by a certain amount of movement in the fixed digit. In Evans (1972) the sclerotised ‘Rollplatte’ node is stated to impinge upon the basal region of the fixed digit which results in the fixed digit (on its own) being pushed upwards so increasing the gape of the fixed digits. It is not clear how this presumed ‘elevator-like’ action could occur without say a weak cheliceral shaft becoming flattened in width and the shaft flexing to a bigger height. If this actually occurs, this would internally squeeze the ‘Rollplatte’ laterally and hold it more firmly. It is not clear to what advantage this is. Similarly, if the moveable digit depressor tendon was pulled strongly beyond when gape would be at a maximum, the head chelal head would be forced down not up. Unfortunately Gwilym Evans cannot now be asked what he meant exactly.

**Fig. 19:**
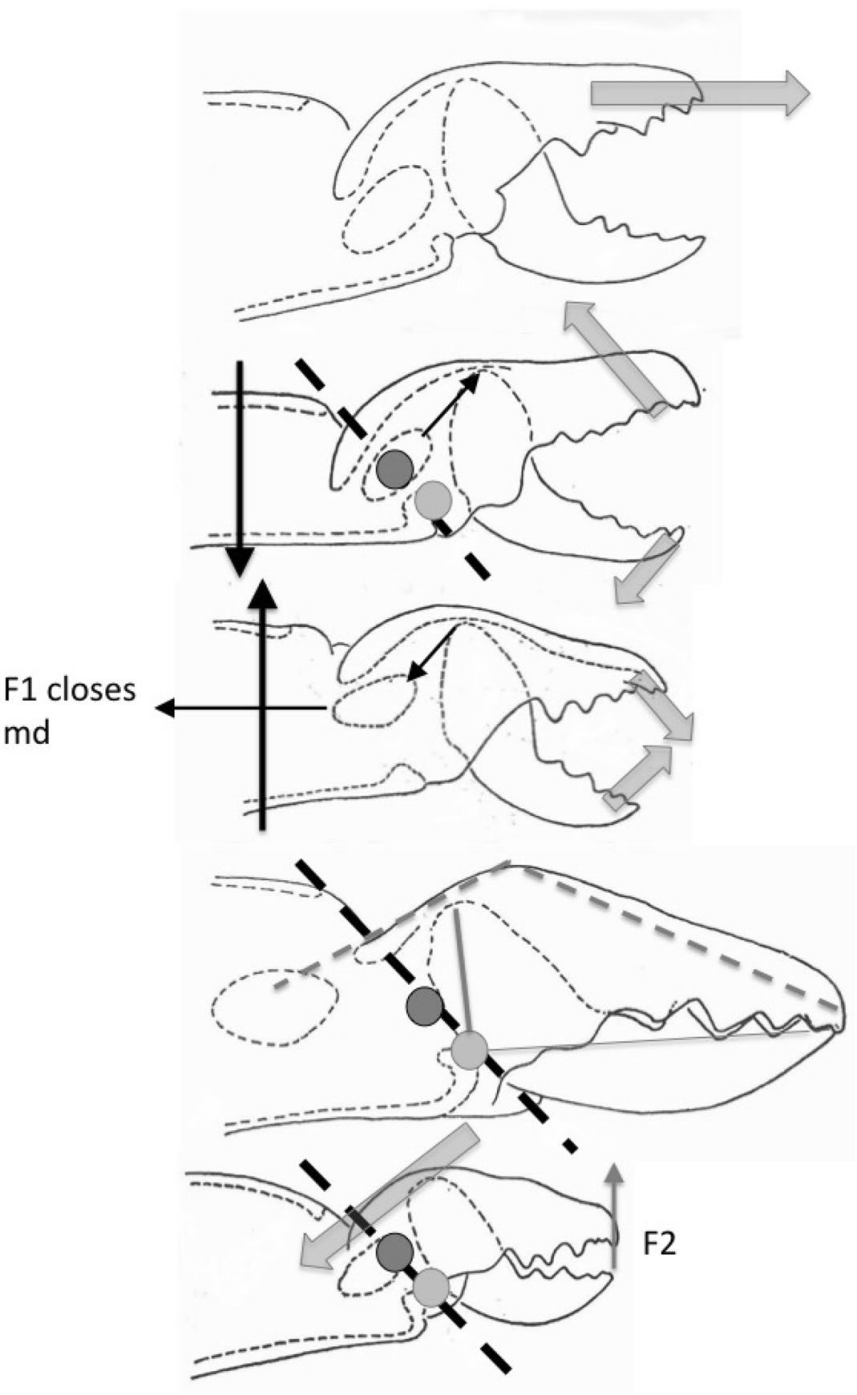
Decomposing the uropodid chelal design assuming the fixed digit tip stabbed ‘open-mouthed’ into food material first. Uropodine chelae with ‘Rollplatte’—annotated composite figure based upon Wišniewski and Hirschmann (1991) © Acta Musei Nationalis Prage with permission. Kolbenpilus omitted. Note depression dorsally behind fixed digit ‘head’. The two rotation points may coincide *From top to bottom* Fixed digit in *Trichuropoda jelineki* n.sp. DN stabbed ‘open-mouthed’ into foodstuffs (grey arrow); Cheliceral shaft lowered (black arrow) in *Trichuropoda proteroamoceri* n.sp. DN prises open fixed digit at flexure point (dashed line) around effective rotation point for chelal head (dark circle) as moveable digit opens and Rollplatte pulled through ‘pinch-point’ of terminus of cheliceral shaft to yield wider gape. Pale grey circle = condyle; Force on the levator tendon closes the moveable digit and pulls the Rollplatte back through pinch-point at the same time as the cheliceral shaft is raised and the fixed digit flexibly falls and returns to default position (grey arrows) in *Trichuropoda saopauli* n.sp. DN; Default closed ‘resting’ static force state for chelal with pale grey circle = condyle in front of and below effective chelal head rotation point (dark circle) and grey dashed outline shape of fixed digit posteriorly lined up with ‘Rollplatte’ in *Trichuropoda tchadensis* n.sp. DN; Resolved effective force on chelal head as grey arrow in *Trichuropoda columbiensis* n.sp. DN arising for continual application of F2 once moveable digit closed (see Figs. 17, 18)

Rather, perhaps the fixed digit tip is first stabbed into food material and movement of the cheliceral shaft within the gnathosoma being drawn down is done in such a way that the chela is effectively prised open before the moveable digit is closed and the chela ‘bites’ (the fixed digit slicing downward at the same time as the moveable digit moves up). This ‘open-mouthed’ scenario is shown in Fig 19. Note Evans (1972) possibly illustrates this in Fig. 56(c) for a female *Trachyuropoda coccinea*. This would be like the action of the upper front teeth of sabre-tooth cats slicing down (Van Valkenburgh 2007) as the jaw closes when the lower jaw is anchored (open-mouthed) in the prey of these animals. A rearward pull by the mesostigmatid under either scenario would then remove a substantial quantity of tissue and create a serious wound. If targeted well this could be quickly lethal for any small prey. This then should be correlated with a fixed digit extension (article 4) being tooth-like or sclerotised. Are there examples of this to be found in mites? One wonders if this degree of ’overhang’ could then be correlated itself with the chelal velocity ratio and so indicate such carnivorous action? Predators with stabbing front canines usually have a fairly short output lever moment arm L2 to avoid prey-induced wobble and dislocation. Is this the case for mites using this fixed digit suggested action? However, any mechanism of chelal head downward flexure needed appears not congruent with the angle of terminus of shaft in the uropodid mesostigmatids, as the ventral strengthening around the condyle would be compromised and the depression dorsally behind the chelal head flattened out by any strong downward flexure. Further any “bounce-back” of the chelal head rising again would effectively require moveable digit opening (or at least no sustained pull on the levator tendon). It is also not clear how this would work for potentially ’soft-nosed’ Kolbenpilus uropodines or any mites with complicated sensory setae terminally (Fig. 14). More investigations of the chelal geometry and feeding of specific uropodids is needed. In particular as to how much independent directional movement is possible for the chelal head versus the cheliceral shaft elements.

**Fig. 20:**
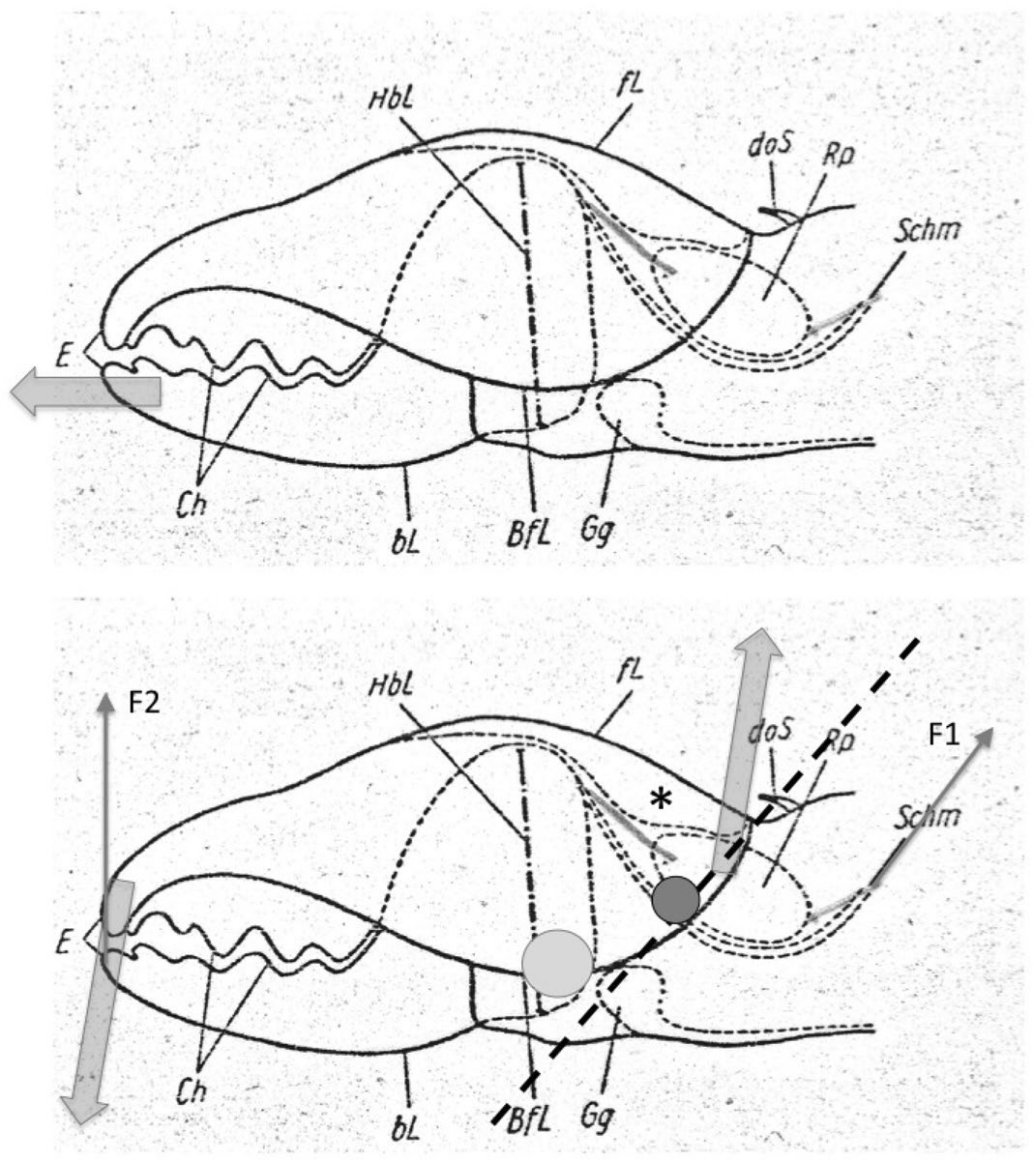
Decomposing the uropodid chelal design assuming the moveable digit tip originally stabbed ‘open-mouthed’ into food material first (like general action of Fig 17*Top*). Scheme for ‘Rollplatte’ strengthened nodule as a pulley for the adductor tendon. *Upper* Fig. 7 in Hirschmann (1956). Note original author also includes separate tendon attachment to the pulley. From: W. Hirschmann: Mikrokosmos © 1956, Franckh-Kosmos Verlags-GmbH & Co. KG, Stuttgart, with permission. Mite illustrated is *Trichouropoda ovalis* (C L Koch 1839) in Hirschmann and Zirngiebl-Nicol (1961) mid-way through process. Grey arrow action of cheliceral shaft or idiosoma pushing moveable digit into foodstuff (initially occurs when chela is open). *Lower* Here pulling on levator tendon induces the rise of ‘Rollplatte’ and a resultant force against the underside of fixed digit dorsal strengthening (*) causing it to rise. Rotation around notional dark grey circle infers that the chelal head (fixed digit tip) flexes downwards (grey arrow) at the same time as the moveable digit exerts closing force F2 through the pulley action at the rear tips head up (grey arrow) making the fixed digit tip act like a sabre-tooth cat upper mandible strike. Note the two rotation points may coincide

Matters are different if the adductive levator tendon actually goes around the ‘Rollplatte’ (yet is still connected to it so as to hold it in position within the cheliceral shaft—Fig. 20 and Hirschmann and Zirngiebl-Nicol 1961—much as the fabella is an anchor point for ligaments in human knees; Hauser et al. 2015). Evans (1972) states unequivocally that this is erroneous (insisting that the ‘Rollplatte’ is developed on the tendon and pulled forward when the moveable digit is depressed). Mašán (2003) however, does illustrate it so in Fig. 1 for *Trachytes* spp. The Rollplatte sits free-floating just underneath the apparent breakpoint in the dorsal chitinisation of the cheliceral shaft just in front of the dorsal sensilla. As such, it would as Hirschmann (1956) says: “Wie die Rolle eines Flaschenzuges vergrößert ein eiförmiges Chitingebilde, die Rollplatte die Zugkraft des Schließmuskels”. This provision of extra leverage (to crack tough material, spores?) being against the upper thickened shaft edge in the narrowing behind the condyle. This thus effectively makes a formal rotation point to flex the fixed digit part of the ‘chela head’ first up (as Evans 1972 suggested?) and then down on the continual muscular pull from within the cheliceral shaft (that could be pointing in a different direction within the prey/food). To illustrate this, consider moving your hand upwards under a partially-sawn-through breakpoint in a piece of wood, if your hand is just ahead of the breakpoint the distal piece of wood will move up. As you move your hand slightly backwards but still move it upwards (equivalent action to the adductor muscle pulling more on the tendon), then your hand becomes directly under the breakpoint and the whole assembly ‘cracks’ at the flexible point and moves up in a V-shape (thus causing the distal and proximal tips to move down). So in this way the chelal head would ‘wiggle’ vertically. While this is happening, the interlocking distal teeth illustrated by Hirschmann (1971) would work like the tines on the edge of a mechanical digger bucket locking it shut. Alternatively, if the moveable digit is stabbed into the prey (as Hirschmann 1956 proposed moveable digit pointed tips are) open-mouthed first then this pulley action would again effectively produce a sabre-tooth strike of the fixed digit tip down into the food. For the strike to be effective against relatively tough material, the fixed digit would need sclerotised strengthening even when tiny in size. Of course, the cheliceral shaft proximal to the idiosoma would be somewhat compromised too by this action (cf. the need for particular neck musculature in sabre-tooth vertebrates). Micro-tomographic investigation is needed to be sure of the order in which things actually happen.

As sabre-tooths in vertebrates generally were large animals, and no large mesostigmatids appear to clearly have structures like a ‘Rollplatte’, an excavation-bucket/‘zirkeln’ analogy of chelal function in uropodids is more strongly indicated. Furthermore, Evans (1972) might be right anyway, as it is not clear how the tendon going around any ‘free’ node would avoid the tension and compression strains in going round a pulley without damage (see Frost 1971, p. 105). It would certainly have to be bendable but hardly linearly elastic.

Having a complicated sensory apparatus on the tip of the fixed digit (5) as in: *Uropoda orbicularis* (Fig. 14), *Cilliba cassidea* (Athias-Binche 1977), *Dinychus* sp. (including *Dinychus appendiculatus*) and *Phaulodinychus mitis* (Krantz 1978), makes sense as a distal ‘sniffing nose’ when the ‘bucket on the excavator-digger arm’ explores in the food material (cf. like the external nostrils at the tip of the beak of the New Zealand Kiwi bird). A complicated seta would be inconsistent with any ‘sabre-tooth’ action of the fixed digit tip (Fig. 20). However, not all uropodoids with a ‘Rollplatte’ have complicated distal setae—*Urodiscella alophora* and *Uropoda leonardiana* does not (Gorirossi-Bourdeau 1997)—the latter has hooked digit tips and a “pocket” like much bigger gamasines Fig. 13. Similarly some like *Phaulocylliba amplior* and *Metadinychus argasiformis* with this sensory ‘snout’ appear not to have a ‘Rollplatte’ (Gorirossi-Bourdeau 1997). Hirschmann (1971) points out that the ratio of the length of the moveable digit (aka L2) to the length of the extension of the fixed digit beyond the tip of the moveable digit is diagnostic of species in nine genera. One wonders if this degree of ‘overhang’ can be correlated with the chelal velocity ratio—much as tapirs (Fig. 14) who have a flexible nose to search out and feed on certain foodstuffs? Do different mites have different ’tools’ on the end of these ‘snouts’ much like the tools on the hoses of domestic vacuum cleaners?

Indeed, perhaps the ‘Rollplatte’ is used *differently* in different species? As said by Richard Phillips Feynman (US educator and Nobel Physics prize winning physicist 1918–1988): “You can know the name of a bird in all the languages of the world, but when you’re finished, you’ll know absolutely nothing whatever about the bird. So let’s look at the bird and see what it’s doing—that’s what counts”. It is *how* the mesostigmatid chelicera is used that matters. Note that Evans (1972) states a distinct sclerotised node is never developed in gamasines. So what is it about uropodoids? Uropodoid diversity increases in the Tropics so a systematic trophic study observing and comparing the feeding of similar size uropodoid mites in a common warmer-clime biological setting with and without a ‘Rollplatte’ and particular fixed digit features is needed.

## Discussion

Flowing from the results above, a series of further assertions can be examined.

### Can a predictive model be built from simple morphology and physics?

**Table 9:** Best *glm* logit linear model and estimates from fitting training data set for ecologist use in the field

Model	Estimate	SE (estimate)
$$logit(p(wormlike\ feeder))= Intercept+Slope\cdot log(sin^{-1}(\sqrt{(}VR)))$$		
Intercept	1.426	1.124
Slope	2.570	1.993

Estimated probability (*p*) $< 0.475$ (worm-like prey feeder) or Velocity ratio (VR) $< 0.276$ infers microarthropod prey feeding habit

**Fig. 21:**
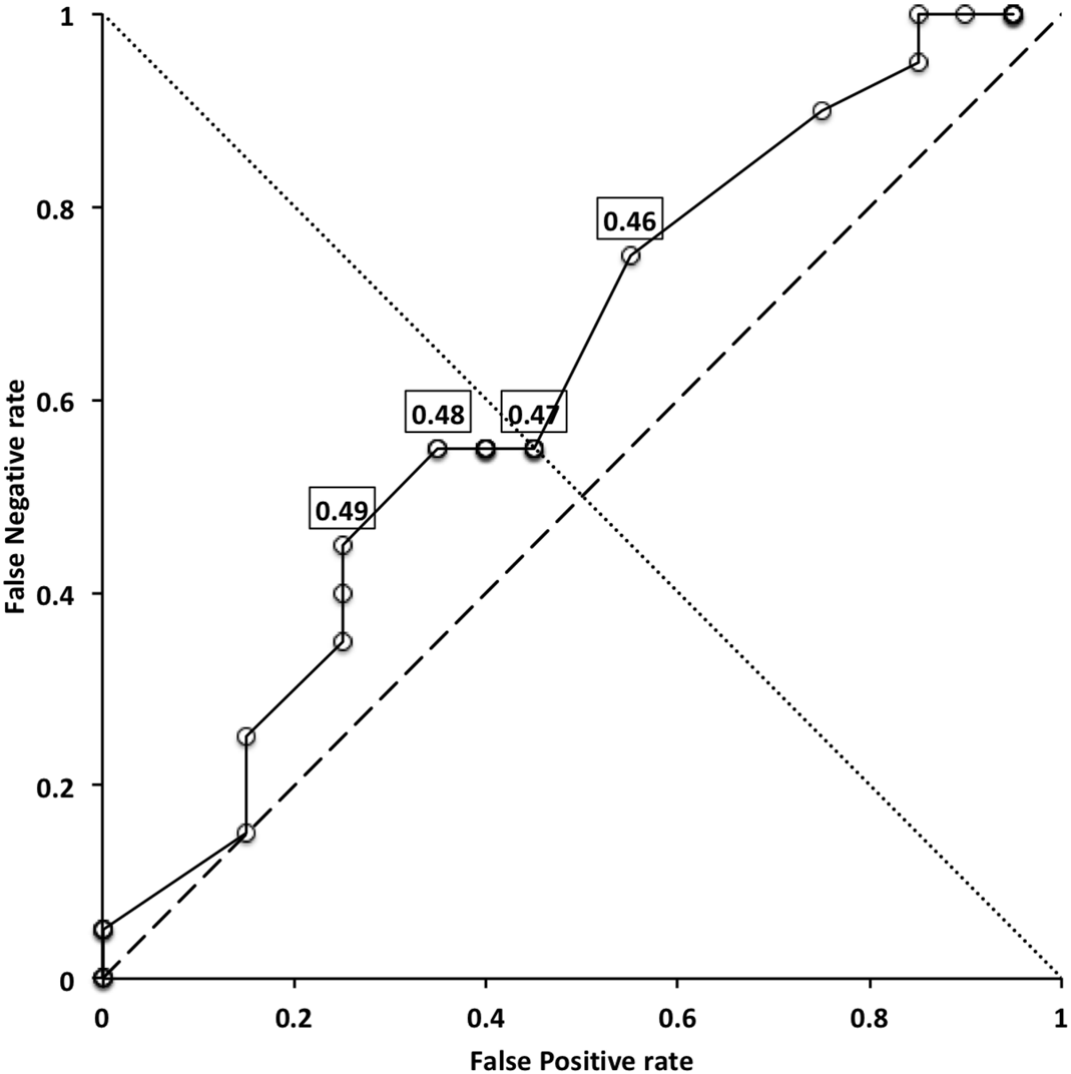
Receiver Operating Characteristic (ROC) curve for training data set—best *glm* logit log model. Field-use curve may be more variable. True positive rate (*Sensitivity* or *p*(*detection*)) versus False positive rate ($1-Specificity$ or $p(false\ alarm)$) for probability value threshold levels of (0...1) in order to claim that the species has a ‘worm-like feeder’ habit. Dashed line is unit slope through the origin for reference ( $\equiv$ a random guess or 50:50 ‘line of no discrimination’). The best possible classifier is a point at maximum orthogonal distance above the reference line ( $\equiv$ estimated maximum positive likelihood ratio) here midway through the range. Illustrative threshold values in boxes. This $p=0.475 \equiv VR=0.276$ which is where (within 3 s.f.) the values of $Sensitivity = Specificity$ cross the observed curve is shown by the dotted line. $AUC(ROC)=0.6225$ giving a ‘2:1 on’ bet

Ignoring any taxonomic niceties for now, the answer is: Yes. All the above results suggests that the mite feeding habit i.e., here as indicated by $p(wormlike\ feeder\ |\ f[data])$, should mainly depend upon the estimated chelal crunch force and chelal velocity ratio (in that order with possibly also chelal gape, cheliceral reach and overall body size in that order of importance afterwards). Mites of small gape (MDL < 150 μm), mites with short reach (CL < 350 μm), and mites with large bodies (IL > 500 μm) should appear particularly distinct. Due to the low sample size, statistical modelling does not clearly detect this. Rather, the best final model is using velocity ratio only and the parameter values for the training data set are shown in Table 9, together with the receiver operating curve of this classifier shown in Fig. 21. Extra main effect or interaction terms for gape, reach, or size did not improve the velocity ratio-based model (*results not shown*). This is all consilient with Fig. 2. The effect of log transform of the symmetrised velocity ratio (Table 4) did not infer any untoward radical allometry with scale (*results not shown*). The $AUC(ROC)=0.6225$ indicates that this velocity ratio-based classifier will rank a randomly chosen positive instance higher than a randomly chosen negative one 63% of the time (a poor classifier would be a random toss of a coin—such a classifier would assign a higher score to a randomly chosen positive example than to a randomly chosen negative example 50% of the time). In other words this classifier is a “63:37” rule or approximately a “2 to 1 on” bet. Whilst not the most impressive classifier, this performance for field ecologists is clearly much better than no knowledge/guesswork (i.e., a 50:50 bet) and crucially yields an estimated probability value (last column ${\hat{p}}$), that other methods do not. The Gini coefficient for the model is 0.245. Despite omnivory being common in animals (see references in Walter and Proctor 2013) and opportunistic predators potentially preying on multiple trophic levels in complicated ecosystem food-webs, the model looks promising.

**Fig. 22:**
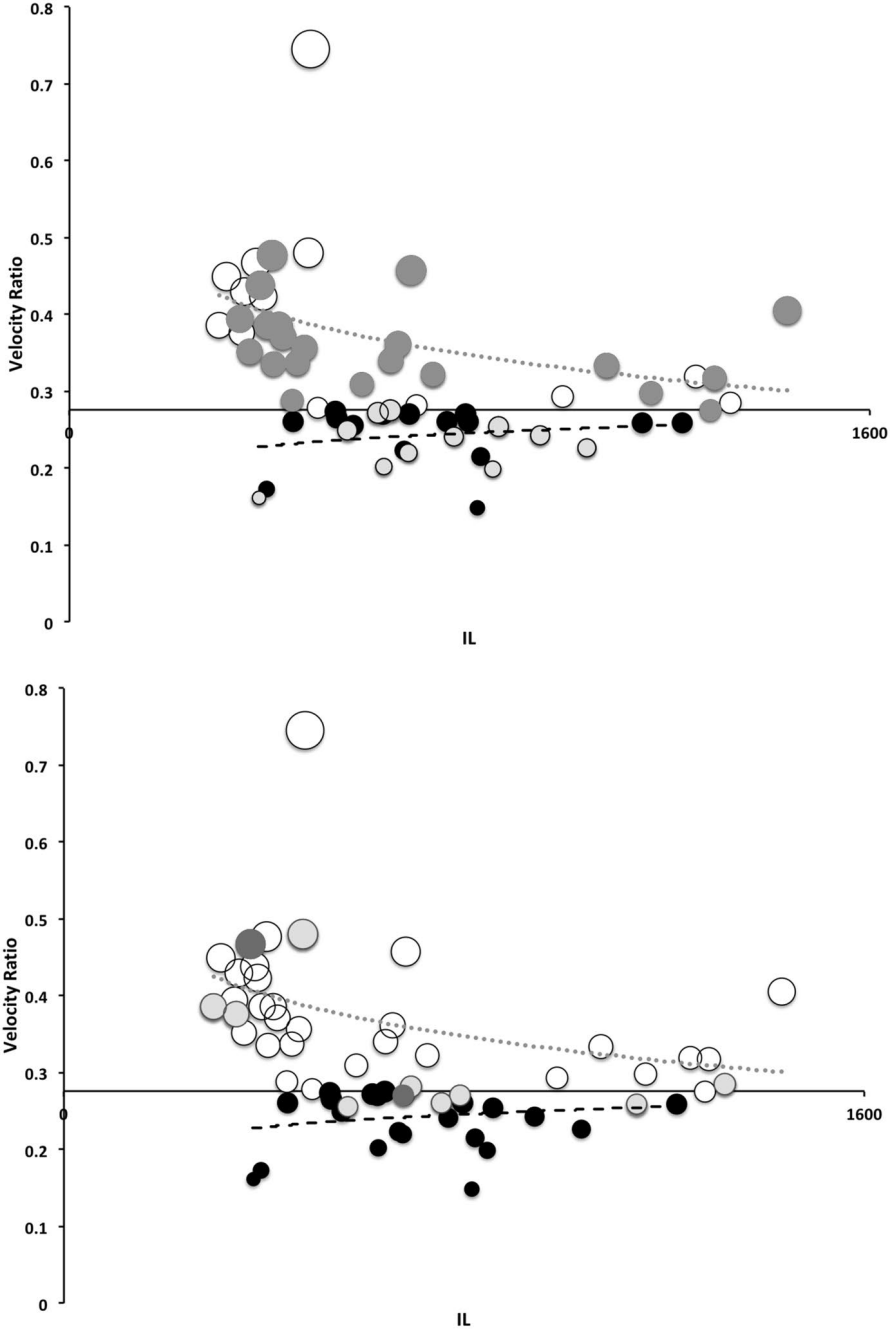
Predicted feeding types for training and test data sets. Bubble size $=p(wormlike\ prey\ feeder)$. Based upon *glm* model in Table 9 (threshold $p=0.475\equiv 0.276$ cut in chelal velocity ratio VR). Two displays for clarity of exposition (see text). *Upper* Clear open circle training set (with grey dotted log regression line *for illustration*) = worm-like feeders. Black filled circle training set (with black dashed log regression line *for illustration*) = microarthropod feeders. Dark grey filled circles are those unknown diet species predicted as worm-like feeders. Pale grey filled circles are those unknown diet species predicted as microarthropod feeders. Note good agreement. *Lower* Clear open circle training set (with grey dotted log regression line for illustration) = worm-like prey feeders. Black circle training set (with black dashed log regression line for illustration) = microarthropod feeders. Pale grey filled circles = training set polyphagous mites. Dark grey filled circles = training set omnivore mites. Note good agreement

The best threshold for this velocity ratio model is equivalent to a cut at $VR<0.276$ which is between the velocity ratio values of *Pergamasus misellus* and *Pergamasus digitulus*. It is at the strength versus speed trade-off point. This is only slightly higher than the mechanical advantage of the cutting tip of the chela in predatory solifugids (0.24–0.26; Meijden et al. 2012b) indicating a possible wider relevance to arachnids. It also agrees with the mechanical advantage of 0.248 for the long sharp-toothed strong chela of the active predatory swimming crab *Macropipus depurator*; Warner and Jones (1976). This species shows intermediate speed muscle fibres compared to the slow “tonic” speed muscles in the heavy blunt-toothed “crushing claw” of the crab *Cancer pagurus* which as a nocturnal predator of molluscs and crustaceans has a mechanical advantage of 0.329 (Warner and Jones 1976). This 0.276 cutoff when applied to the test data set yields predictions in Table 8 and Fig. 22. Of the extra mites added to this study with known feeding types, five out of six (*Alliphis halleri*, *Parasitus coleoptratorum*, *Uropoda orbicularis* and *Veigaia nemorensis* (new)) are predicted perfectly. In particular the large fitted probability of being a worm-like feeding type for *Alliphis halleri* agrees with Sadar and Murphy (1987)’s clear experimental feeding result and Karg (1983)’s view that all European species of *Alliphis* are exclusively nematophages. Its velocity ratio at 0.357 is almost exactly that of the major crushing chela of the shore crab *Carcinus maenas* (Warner et al. 1982). The positive control of *P. coleoptratorum* has also worked. *Parasitus fucorum* has an almost 50:50 bet on its prey type consilient with the ambiguous feeding records in the literature. Only the prediction for *Polyaspis* n.sp. is divergent (however Walter and Proctor 1998 do state that *Polyaspis* is an aggressive predator of small invertebrates ingesting fluids only). Note that the velocity ratio of the chela of *Veigaia exigua* (Table 3) almost exactly matches the 0.177 mechanical advantage design of the fast chela of the *Macropurgus depurator* known to have more fast “phasic” muscle fibres proportionately (Warner and Jones 1976). This is consilient with the interpretation that *Veigaia* spp. are ‘fast-snappers” of vagile prey. Of the polyphagous species from Buryn and Brandl (1992): *Rhodacarellus sileciacus*, *Arctoseius certratus*, *Blattisocius keegani*, *Pergamasus runcatellus*, and *Pergamasus septentrionalis* are predicted as more like the worm-like prey specialists; *Cheiroseius borealis*, *Hypoaspis aculeifer*, *Pergamasus runciger* and *Pergamasus crassipes* are predicted as more like microarthropod specialists. Three out of four of the *Parasitus* spp. (Table 8) are predicted to be worm-like prey feeders much as Weis-Fogh (1948) observed as although larger soil-dwelling parasitids would feed upon collembola and acarid mites—they had a distinct preference for nematode worms. The ‘unknown’ food type pergamasids are predicted as expected as micro-arthropod feeders (Krantz 1978) despite Witaliński (1971) rearing them on enchytraeids. The omnivore *Proctolaelaps pygmaeus* is predicted to fall amongst the worm-like feeding specialists as expected. The omnivore *Trachytes aegrota* is predicted to fall amongst the microarthropod feeding specialists. *Urodiaspis tecta* shows adaptations (see also Table 3) consilient with its known habitats (Bal and Özkan 2007). Specialist feeding phytoseiids are predicted to be more like worm-like feeding predators (‘crushers’) than that of the generalist feeding phytoseiids suggesting that they do favour semi-immobile small soft immature tetranychids as food. Whether this distinction matches other phytoseiid life-style traits (e.g., Crofti et al. 2004) awaits confirmation, as does the examination of a wider set of phytoseiid genera: e.g., *Amblydromella* and *Anthoseius*; see Denmark and Welbourn (2002); *Euseius*; see Basha et al. (2002). The strongly eudaphic rhodacarids which are characteristic of the deeper layers of mineral soils and interstitial spaces down to saturation with ground water (Hughes 1959; Walter and Ikonen 1989) are correctly predicted as (small-pore) nematophages (Krantz 1978).

**Fig. 23:**
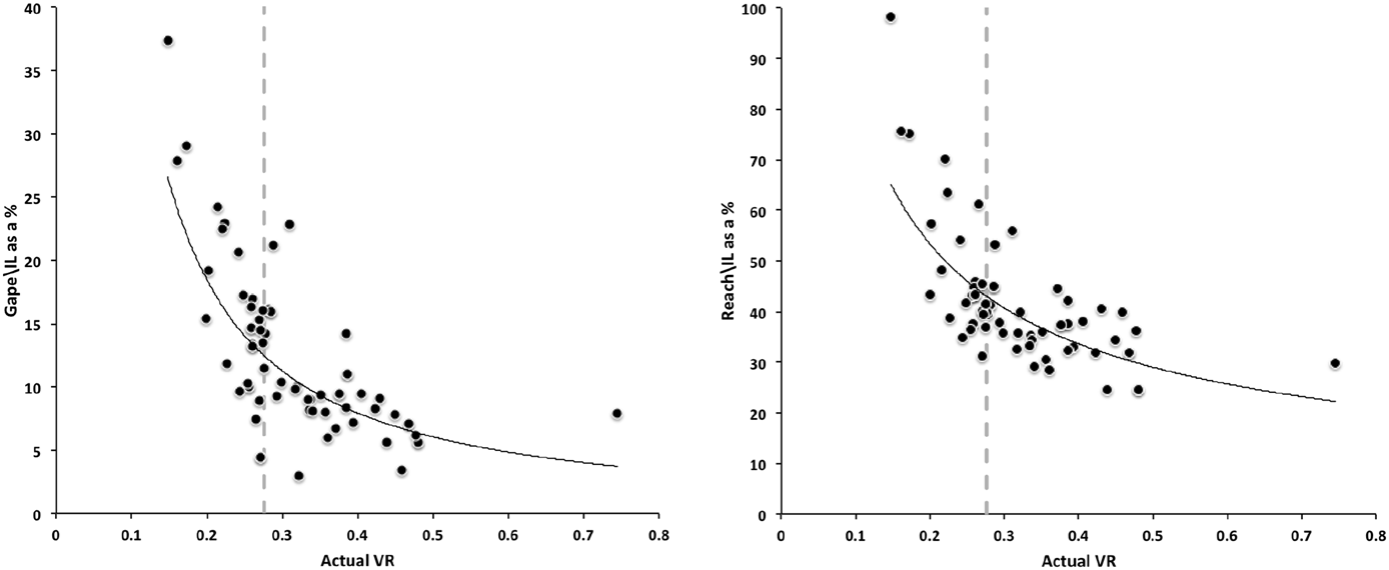
Large and small mites have different constraints. The *glm* model in Table 9, threshold $p=0.475\equiv 0.276$ cut in velocity ratio (VR) matches Buryn and Brandl (1992)’s morphometric conclusion. Black circles = training and test data sets. Grey dashed vertical line is threshold. Thin black trend line as power function simply to illustrate effective ‘break-point’ regression at threshold, between a scaling for one trophism and a scaling for for the other. Relative measures on the y-axis do not essentially vary much for worm-like prey feeders but show concerted changes for micro-arthropod prey feeders. *Left* Relative gape versus actual velocity ratio (predicted micro-arthropod feeders to the left have excessive gape for body size; worm-like prey feeders to the right do not). *Right* Relative reach versus actual velocity ratio (predicted micro-arthropod feeders to the left have excessive reach for body size; worm-like prey feeders to the right do not)

Colloquially the threshold in the model is where a chelal crushing adaptation switches to a cutting adaptation (and vice versa). It is in the 0.248–0.329 range which marks that change in brachyuran crab chelae (Schenk and Wainwright 2001). It is consilient with Buryn and Brandl (1992)’s conclusion (Fig. 23) explaining their log/log residuals empirical result. It is in accordance with their PC2 loadings on the (relative) length of the teeth row on the chelal digits, as they become ‘blades’ at low velocity ratio (VR) values. In effect, the threshold is the point when morphology to cope with vagile prey must dominate trophic considerations. One suggests that this is the switch point from a chelal muscular mechanism capable of slow strong movements to that of one with quicker weaker ones—as one cannot have both (Manton 1958a). Histochemical confirmation of muscle types between mesostigmatid crusher style chelae and cutter style chelae (as in lobsters; Lang et al. 1980) would be useful. The velocity ratio (VR) model is commended for use.

### Deploying and testing the classifier

**Fig. 24:**
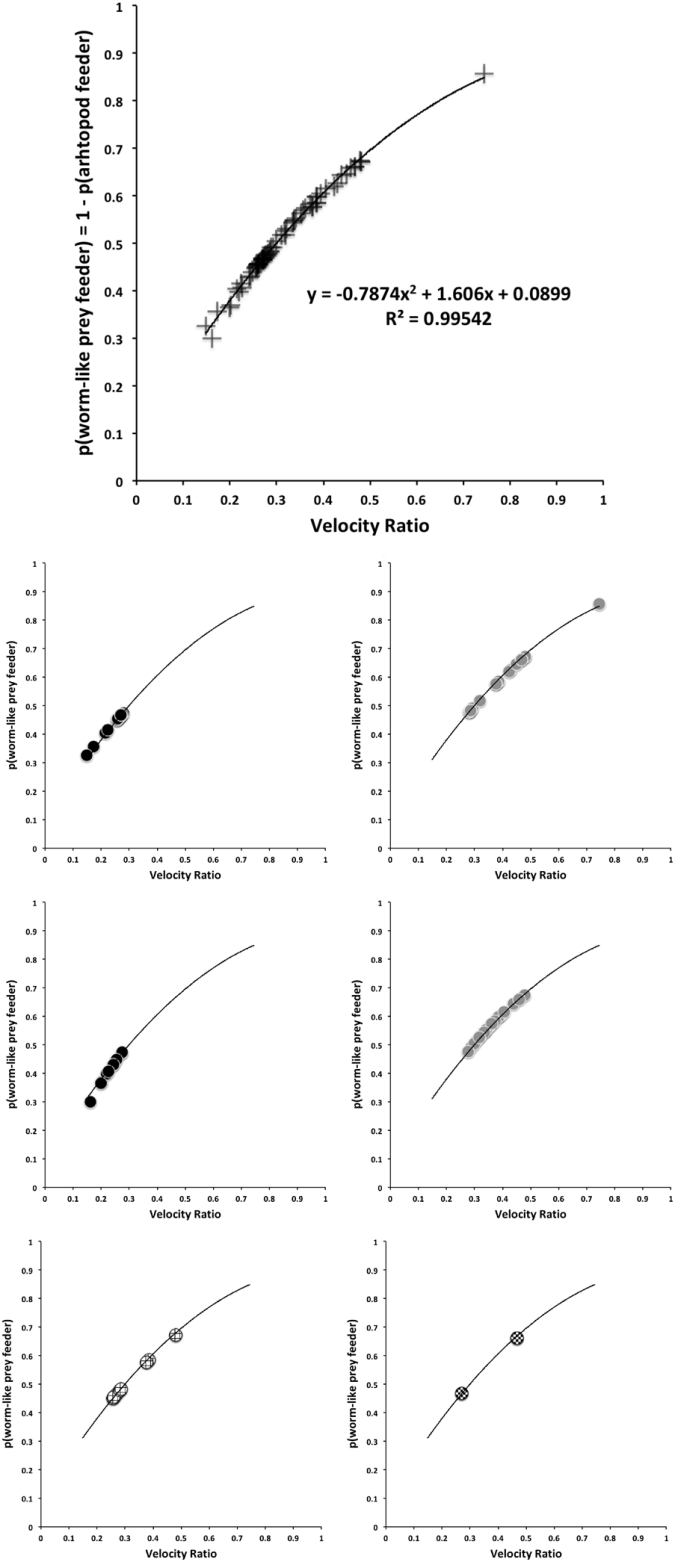
Simple Excel nomogram based upon *glm* model in Table 9, threshold $p=0.475\equiv 0.276$ cut in VR. *Top Row* Overall equation. Crosses are all data points (training and test data sets). *Second Row* Actual micro-arthropod feeders on *Left*, Actual worm-like prey feeders on *Right* ex Buryn and Brandl (1992). *Third Row* Predicted micro-arthropod feeder who were ‘unknowns’ before on *Left*, Predicted micro-arthropod species who were ‘unknowns’ before on *Right*. *Bottom Row*: Polyphagous mites on *Left*, Omnivores on *Right* ex Buryn and Brandl (1992)

Indeed, the field ecologist does not even need to do complicated calculations. Fig. 24 shows a simple quadratic line that can be used instead—suitable for calculation in Excel of the expected $p(wormlike\ prey\ feeder)$ from the observed free-living mesostigmatid mite’s chelal velocity ratio. It yields a probability value to instil appropriate confidence. As in lobsters (Warner and Jones 1976), the “crusher” chelal design (cf ‘worm-like prey’ feeding) has a higher velocity ratio (presumed mechanical advantage too) than the “cutter” chelal design (cf ‘micro-arthropod like prey feeding’). This straightforward easy-to-implement classifier, encompassing the design trade-off of chelal sustained strength versus agile closing speed, now needs field testing by ecologists and further validation by laboratory acarologists targeting *specific* mesostigmatid species with structured feeding experiments (like Zipser and Vermeij 1978), plus detailed observations and biochemical/radioisotope confirmatory work of prey ingestion in the wild (see Perdomo et al. 2012 for instance). Possible good places to start of interest practically for applied acarology could be: the genus *Laelapsis* (Kazemi et al. 2016); *Lasioseius scapulatus* (Imbriani and Mankau 1983), or *Hypoaspis (Geolaelaps) milesi* or *Stratiolaelaps* used as predatory biological control agents of sciarid larvae and thrips in greenhouse crops (is the closely related 108 species genus *Cosmolaelaps,moreira* and Moreira et al. (2015) also designed similarly?), or *Hypoaspis aculeifer* used in bulb mite control in Europe, or *Scamaphis* new genus (Eviphididae) assumed to be a soil nematode feeder; Karg 1971, or *Phytoseius hawaiiensis* which attacks lychee erinose mite *Eriophyes litchi*, or the omnivorous pest of *Drosophila* cultures *Proctolaelaps regalis*, Houck 1993, or *Proctolaelaps bickleyi* and other mites investigated for biological control of pests (Duarte et al. 2018), or other *Proctolaelaps* spp. known in southern and western corn root-worm cultures feeding on *Tyrophagus putrescentiae* and histiostomids, etc, etc.

It could be very illuminating to deploy this classifier on halophilous littoral and estuarine mesostigmatids (like *Thinoseius* spp.; Evans 1954a) across a transect from the lichen splash zone down to the *Fucus serrata* zone (Halbert 1920 and Appendix B in Evans et al. 1961; Krantz 1978). Pugh and King (1985) found *Hydrogamasus salinus* to be a general predator, and while *Eugamasus immanis* feed voraciously on small oligochaete worms in tidal drift (King 1913) little is known of the feeding habits of *Halolaelaps marinus* or *Cyrthydrolaelaps hirtus* restricted to the lower zone. The latter does feed upon anurid springtails (Pugh and King 1985)—what are the chelal velocity ratios of these mites? Are the latter species designed to feed upon Polyzoa, Hydroids, minute annelids and halacarids? Do the chelicerae of *Hydrogamasus giardi* best match them feeding upon nematodes by crushing or feeding upon springtails by slicing (like *Anura marina*—Pugh and King 1985)? Another possibility is to express the co-occurence of gamasines in coastal meadows (see Table 1 of Salmane 1999) in terms of their cheliceral design. Does this explain their community structure?

As a veigaiid family member, does *Cyrthydrolaelaps* have strengthened chelal levator tendons or not (cf. Table 7)? What is the complement of velocity ratio values for gamasines and uropodids in the stored food ecosystem (Krantz 1978) or in the nidicolous biome (where there is little certainty of food habits; Evans et al. 1961)? What does the design of the chelicerae in nest-dwelling *Phaulodinychus euris*, *Euryparasitus emarginatus*, or *Proctolaelaps hypudaei* look like? Trachyuropodoids (Krantz 1978) would be interesting to examine too. Would velocity ratio (VR) better explain co-existence and resource partitioning amongst myrmecophilous mesostigmatids (Donisthorpe 1927; Campbell et al. 2013)? Would chelal velocity ratios even explain how the parasitic spelaeoryhnchids are adapted to hang on tenaciously to their bat hosts (Fig. 32-2 in Krantz 1971)? Many other suggestions could be made.

Polyphagy and omnivory in small mesostigmatids is indicated once the velocity ratio approximates 0.4 (Fig. 22*Lower*). This value is close to the mechanical advantage of the main crushing tooth in solifugid chelae (0.44–0.47 see Meijden et al. 2012b) again suggesting a possible wider importance in arachnids as a whole. Apart from stabbing plants and perhaps lapping up exudates/honeydew/nectar (Krantz 1978; Adar et al. 2012; Liu et al. 2017), herbivory/saprophagy requires velocity ratio values like astigmatids and cryptostigmatids. Would examining the velocity ratio complement of species help explain the co-existence of multiple free-living phytoseiids on plants (Collyer 1956)? What is the pattern of velocity ratio values for those predatory mites that coexist in mushrooms (Binns 1973) or, for dung living congeners or, for those mesostigmatids in the bark beetle gallery community web (Lindquist 1969; Chaires-Grijalva et al. 2016)? Is there any correlation between velocity ratio values and being a known “cruise predator” (Walter and Proctor 2013)? Do generalist predators who show significant intra-guild predation (Walter and Proctor 2013) have chelae with particular velocity ratios? Is there any evidence of Hutchinson size ratios (Walter and Proctor 2013) in cheliceral measures (like the velocity ratio or its components L1, L2)? Could there be cheliceral character displacement in *Lasioseius subterraneus* nematode-eating populations with and without the presence of *Geolaelaps* (or the cunaxid *Coleoscirus simplex* and other predators—see references to the Floridian potted plant glasshouse system by Walter, Kaplan, Lindquist and others in Walter and Proctor 2013). There is much that a field ecologist could do.

### Does pollen feeding have a special design?

Adaptations for pollen feeding in phytoseiids are discussed in Flechtmann and McMurtry (1992). Pollen grains are $15\text {-}200\,\mu \text {m}$ in diameter, so it is unlikely that phytoseiids will have evolved special oral structures to hold them before piercing/crushing them like in the acariform Nematalycidae (Bolton et al. 2015) which feed upon very small single-celled eukaryotes and prokaryotes ($<10\,\mu \text {m}$). Indeed, all the mesostigmatids listed in Table 3 (including the uropodoids) have an estimated gape value greater than the smallest pollen grain—some could even tackle the largest pollen grains. Phytoseiid mesostigmatids can survive and reproduce on fungal spores, pollen and plant tissues (Evans 1979) as well as eating tetranychoid mites. Do they have velocity ratios indicating polyphagy?

All three phytoseiid groups from Liu et al. (2017) show:

- a typical (non-uropodine) relationship of cheliceral reach with chelal gape;
- a close fit of chelal crunch force (F2AV) versus cheliceral reach to that of the mites from Buryn and Brandl (1992) and not to that of solifugids;
- a close fit of F2AV versus chelal gape to that of the mites from Buryn and Brandl (1992) (*Figures not shown*).

Phytoseiids are thus typical gamasines. However, Fig. 8*Upper* shows that, compared to more specialist and generalist carnivorous species, pollen-feeding phytoseiids are typified by high DPFD/VPFD values. This is true for species from both Adar et al. (2012) and Liu et al. (2017). Their summary index, suggested as a plant feeding adaptation by Adar et al. (2012), perhaps indicates a similar ‘stabbing’ and ‘lapping up fluid’ mode for feeding on pollen rather than breaking them open? However, these mites also have moderate chelal velocity ratio values which agree with Buryn and Brandl (1992)’s two phytoseiids studied—see Table 6. Their velocity ratios are predictive of an overall worm-like crushing/mashing feeding habit much like the uropodines and the omnivorous/polyphagous mesostigmatids in Buryn and Brandl (1992). The phytoseiid gape measures are also very small suggesting microphagy (Fig. 8*Lower Left*)—perhaps thus they feed upon the smaller stages or eggs of *Tetranychus*? It is worth pointing out that some small spiders with no teeth on their chelicerae do not macerate or reduce their prey to a pulp—Bristowe (1954)—rather, with usually small fangs they suck dry their prey through tiny punctures so it still retains its original appearance. Perhaps phytoseiids act similarly—the apparent crushing nature of the design of their chelae just being used for one chela to simply hold or manoeuvre the non-motile foodstuff while the other one pierces it, and vice versa? Is the phytoseiid design thus a multi-functional compromise?

With the exception of the anomalous position of *Euseius utilis* derived from Liu et al. (2017), the pollen feeders are best summarised by a small-gape, (soft?) worm-like feeding habit regression from Fig. 10. Would this design be also confirmed for the known pollen feeding *Rhinoseius* spp. (Ascidae) or *Neocypholaelaps* spp. (Ameroseiidae) (Krantz 1971, 1978) or is the velocity ratio of *Neocypholaelaps* like other ameroseiids or even phoretic parasitid DNs? The description on p. 175 of Evans (1992) does describe a distinctive chelicera. Is this small-gape (?soft) worm-like feeding habit design also true of *Neoseiulella* spp. with broadly comparable moveable digit lengths (Kanouh et al. 2010)? Overall, there was no clear agreement of the individual generalist and specialist phytoseiid species to a particular regression line with gape for the results from Adar et al. (2012) (*not shown*), but the position of Liu et al. (2017)’s non-pollen feeding groupings suggest that any carnivory is designed like micro-arthropod feeding in general with respect to gape. As a group, phytoseiids have modest cheliceral reach, and Fig. 8*Lower Right* indicates also that at least the generalist carnivores may be designed more like micro-arthropod feeders (i.e., a slicing/cutting feeding behaviour). Specialists have low reach and high velocity ratio values suggestive of egg feeders (i.e., mashing/chewing behaviour). Do they preferentially deploy their pedipalps rather than leg 1 in feeding? However, surely other physiological reasons must also determine the exact prey specificity amongst the carnivore phytoseiids? Is a competency for the physiological handling of plant secondary metabolites (indirectly ingested with herbivorous prey) a more important factor? More research work is needed.

### Is mesostigmatid ‘cephalisation’ important?

In other animal classes investment into various features of the head (‘cephalon’) are important in determining the mode of feeding (e.g., Aguilar-Medrano et al. 2011). In primates, kyphosis (the angular or retraction of the face relative to the neurocranium and/or cranial base) is important. Snub broad headed fish tend to have mouths looking straight on or downwards to browse food very nearby the jaws (or benthically). In contrast, pointed head damselfishes have mouths that tend to look up or straight on. Mouth height and width are positively correlated with prey size but negatively correlated with jaw dexterity and suction force in fish (refs in Carlson and Wainwright 2010). Does considering these facts inform mesostigmatid designs? Given that in free-living mesostigmatids the chelicerae are enclosed under a gnathotectum, and that the height of the distal cheliceral segment (HDS) is strongly correlated with WDS (Table 1, $R^2=0.925$), a reasonable estimate of the narrowness of the ‘feeding head’ in such a mite is twice the width of the distal cheliceral segment (WDS). Clearly larger mites should all other matters being equal have larger feeding heads, so Table 10 shows a categorisation based upon residuals from regressing twice the width of the distal cheliceral segment against the idiosomal index over all mites in this study. This excessive gnathosomal width relative to body size is used to denote mesostigmatids as micro-, meso- or mega-cephalic *for that body size* (Lindeman 2000) to see if this concept has any merit.

**Table 10:** ‘Feeding head’ categorisation of full data set (training + test)—see text, sorted alphabetically within classification. Micro-cephalic = gnathosomal width in bottom third range of residual values relative to body size. Mega-cephalic = gnathosomal width in top third range of residual values relative to body size. All other species deemed Meso-cephalic. $R^2$ for WDS versus WBS = 0.996

Cephalic status	Species
Micro-	*Androlaelaps casalis*
	*Eviphis ostrinus*
	*Geholaspis longispinosus*
	*Glyphtholaspis confusa*
	*Pachylaelaps furcifer*
	*Pachylaelaps leauchlii*
	*Porrhostaspis lunulata*
	*Trachytes aegrota*
	*Urodiaspis tecta*
	*Uropoda orbicularis*
Mega-	*Alliphis siculus*
	*Geholaspis* sp.
	*Parasitus lunaris*
	*Pergamasus mirabilis*
	*Pergamasus quisquillarum*
	*Pergamasus runcatellus*
	*Pergamasus septentrionalis*
	*Pergamasus* sp.
	*Rhodacarellus epigynalis*
	*Rhodacarus agrestis*
	*Rhodacarus strenzkei*
	*Veigaia cerva*
	*Veigaia nemorensis* (new)
	*Veigaia nemorensis* (old)

Several points can be made.

Firstly, as twice the width of the distal cheliceral segment is essentially a diameter, a power scaling relationship over species against mass (here estimated as $IL^3$) of 0.33 is expected (Alexander et al. 1979). In fact a power fit of the width of the distal cheliceral segment (WDS) yields $exponent=0.297, R^{2}=0.471$ suggesting gnathosomal width scales slightly less than expected over species—it remains relatively narrower rapier-like at greater size. Gnathosomal width under-scaling could be indicative of a greater investment in the idiosoma perhaps, especially amongst larger mites to increase its moment of inertia and give stability when dealing with struggling prey. Alternatively this trend towards microcephaly, given the discovered strong elongation axis across these species with size (see above) and Buryn and Brandl (1992)’s original morphometric result, would point to a possible partitioning of resources within the gnathosoma between length and width in forming trophic specialisations over evolutionary time. Given this, any species in this study deemed mega-cephalic from its residual is definitely ‘swimming against the tide’ (i.e., going against the trend). This is particularly true for some of the larger mites studied like *Pergamasus quisquillarum* and *Pergamasus septentrionalis*. They have much more gnathosomal broadening than expected. Could some be Tyrannosaur-like? *Pergamasus septentrionalis* for sure can tackle heavily armoured oribatids (Peschel et al. 2006).

**Fig. 25:**
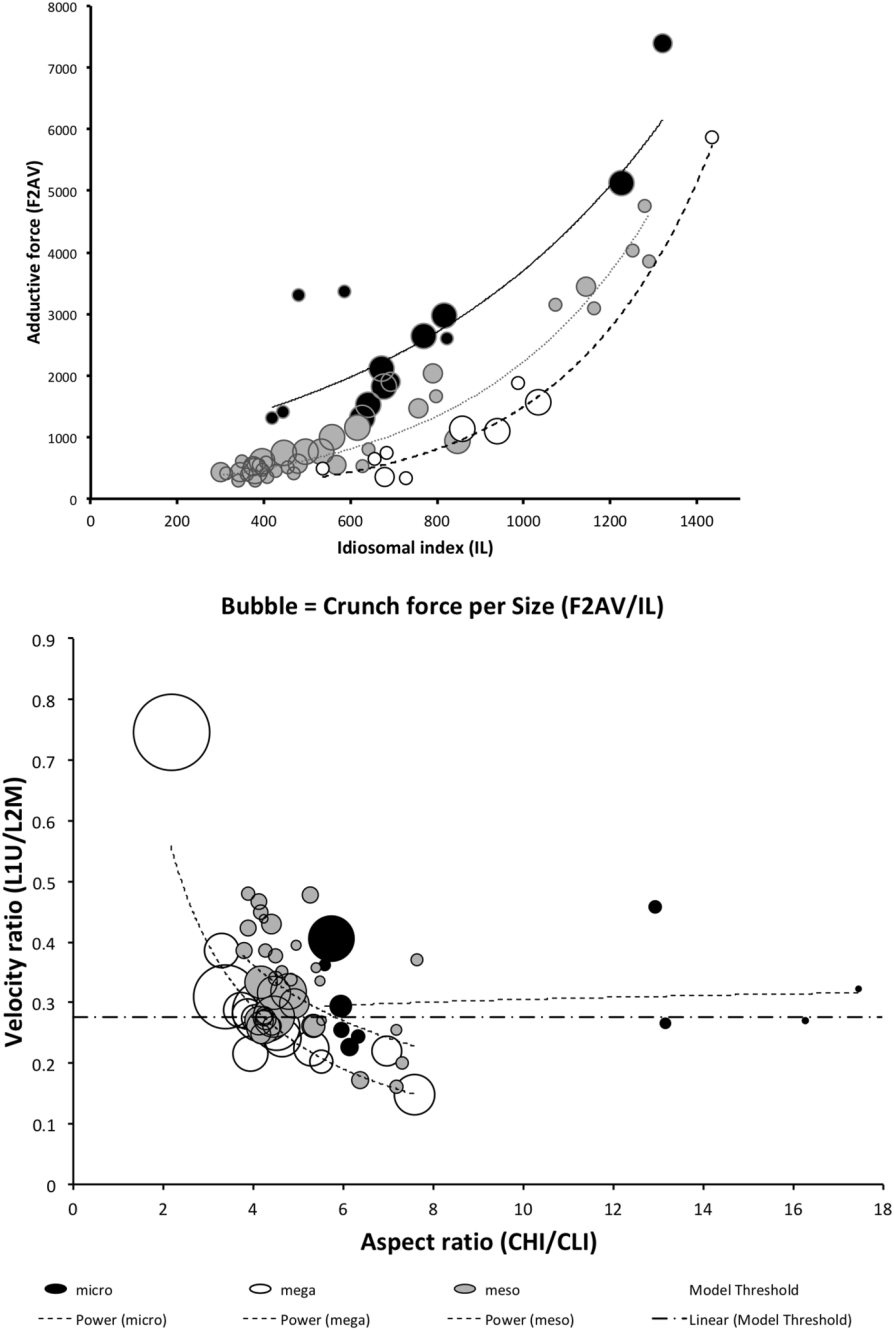
Similar scaling relationships fractionated by degree of cephalisation. *Upper* Chelal adductive (‘crunch’) force (F2AV) with body size (IL) over free-living mesostigmatids for full data set (training + test). Bubble size is recorded/predicted feeding habit (small = worm-like prey, large = micro-arthropod prey). Exponential trend lines for illustration only. Black circles and solid trend line = ‘mega-cephalic’ species (see text). Grey circles and faint grey trend line = meso-cephalic species. Open circles and dashed trend line = micro-cephalic species. The two small size outlier mega-cephalic species are *Alliphis siculus* and *Rhodacarus strenzkei*. *Lower* Velocity ratio versus cheliceral aspect ratio showing crunch force (F2AV) per unit body size (IL) for full data set (training + test) of free-living mesostigmatids. Diagnostic velocity ratio between crushing-style kill design and cutting-style kill design indicated by broken dashed horizontal line. Note including certain microcephalic uropodoids to the right (*Uropoda orbicularis*, *Eviphis ostrinus*, *Trachytes aegrota*, *Urodiaspis tecta*—see Table 3) forces power regression line flat

Secondly, despite the earlier conclusion that increased velocity ratio is usually driven by increasing lever arm L1 not by decreasing L2 in arthropods (and thus an expectation that gnathosomal width would increase accordingly), the form of the relationship of adductive force (F2AV) with body size (IL) is similar (Fig. 25*Upper panel*) regardless of gnathosomal width. Unlike in female lizards (Vanhooydonck et al. 2010) there is no clear correlation of cephalisation with feeding type in these mites overall. Note that mega-cephalics have a noticeable bigger reach (i.e., larger CL—*results not shown*). However, the picture is actually more nuanced because at any one body size, bite force *is* correlated with (relative) head size. Within the mega-cephalic species, for any one chelal adductive force range, a switch from a worm-like (mashing/crushing habit) feeding to a slicing/cutting habit of micro-arthropod predation occurs on increased body size (with a switch around $\text {IL} = 585\text {-}615 \mu \text {m}$—recall the ‘generalist’ IL 450-649 range from before). Of the 14 mega-cephalics, those six mega-cephalics with aspect ratio $<4.00$ are *all* worm-like prey feeders (including *Pergamasus runcatellus* deemed to be a generalist feeder by Buryn and Brandl 1992). They comprises four of the highest velocity ratios in the 14. Those with an aspect ratio $> 4.00$ are *all* microarthropod feeders (including *Pergamasus septentrionalis* deemed to be a generalist according to Buryn and Brandl 1992). A clear trophic correlate in broad headed predators *is* thus detected. This suggests that *Alliphis siculus*; *Rhodacarellus epigynalis*, *Rhodacarus strenzkei*, *Rhodacarus agrestis*, *Pergamasus runcatellus*, and *Geholaspis* sp. are mashing/crunchers of relatively nearby modest size prey. Rhodacarids can be reared on astigmatid mites (Barbosa and de Moraes 2016) but the preferred prey of *Rhodacarus*, *Rhodacarellus* and *Gamasellodes* are nematodes Evans (1992). The relative reach (CL/IL) for *Alliphis siculus* is particularly low (0.21) suggesting its food is not tackled unless very close. Is the gnathosomal orientation of rhodacarids therefore also somewhat downwards ($\equiv$ klinorhynchy with respect to their body axis)? Drawings in Schweizer (1961) suggest perhaps not. Do these elongate mites therefore have to wriggle through the eudaphon until feeding upon their prey almost directly in front of them to attack? Is this why they do not apparently impale their prey on their ancillary gnathosomal structures (Lee 1974) like the airorhynchid pergamasids (see Zukowski 1964)? So is salivary stylet positioning a key indicator here (see Evans 1992, p. 166)? More biological observations are needed.

Amongst those parasitid genera with multiple species assayed (i.e., *Parasitus* and *Pergamasus*), about a third are mega-cephalic. However, there is no clear correlation with reach (CL) or crunch force (F2AV) in them, although perhaps there is a hint that mega-cephalic parasitids might have a bigger gape, a higher aspect ratio and a lower velocity ratio. For sure, the mega-cephalic status of the two largest veigaids studied (*V. cerva* and *V. nemorensis*) well matches their excessive reach, excessive gape and large crunch force. It would appear that these two particular species may illustrate a “runaway morphological process” of trophic specialisation for direct attack of prey in front of (or above) them as their relative reach is $>0.65$. They are thus crocodile-like. One could consider them as ’hypercarnivores’. So, do they pull-back and laterally shake their prey to death when it is held; Tseng and Stynder (2011)? Would the inertia of any large prey holding it effectively still, magnify the slicing and lacerating action of blade-like digits (much as occurs with the faceted teeth of sharks; Springer 1961)? The bottom 10 species in decreasing values of aspect ratio were: *Pergamasus* sp. − 4.02, *Geholaspis* sp., *Dendrolaelaps foveolatus*, *Blattisocius keegani*, *Pergamasus runcatellus*, *Rhodacarellus sileciacus*, *Rhodacarus agrestis*, *Rhodacarus strenzkei*, *Rhodacarellus epigynalis*, and *Alliphis siculus* − 2.17. Seven of these are categorised as mega-cephalic (the remainder as meso-cephalic) again suggesting concerted trophic evolution in mesostigmatids. Is this at all related to how such mites might use their palps, first and second pairs of legs in prey capture like *Antennoseius janus* (Walter and Proctor 2013)?

Thirdly, all 10 micro-cephalic mesostigmatids were encompassed in the top 17 species with the largest cheliceral aspect ratios ($>5.6$), suggesting concerted evolutionary adaptations (Table 3). *Androlaelaps casalis* is known to be a regular fluid drinker and feeder of other mites and their eggs (whereby the contents of the prey is sucked completely out McKinley 1963). It would be tempting to conclude that its prey is first ruptured by a crushing action (high chelal velocity ratio), then an elongate cheliceral apparatus is inserted (fairly high cheliceral aspect ratio) which differentially with respect to its size forms a microcephalic ‘drinking straw’ (https://en.wikipedia.org/wiki/Drinking_straw). This tubular assembly formed of gnathotectum, palps, hypostome etc encloses an elongate tube of prey fluids through which the mite drinks. Surface tension is such that a tube like this would form, maintain itself, and be contiguous with the tritosternal/deuterosternal groove recycling of spilled liquids (Wernz and Krantz 1976) and coxal fluids (Bowman 2017a, b). Do other haemogamasids feed like this (Keegan 1956)? Is this how the slightly klinorhynchid powerful insect egg crushing *Glyphtholaspis confusa* also feeds? Do parasites like *Bdellonyssus bacoti* or *Steptolaelaps heteromydis* or *Varroa destructor* have a similar tubular assembly like this through which prey fluids are sucked (Gorirossi 1950; Furman 1955; Li et al. 2019, respectively)? Acariform mites have repeatedly evolved styliform chelicerae with added morphological adaptations to facilitate fluid feeding (Bolton et al. 2018).

**Fig. 26:**
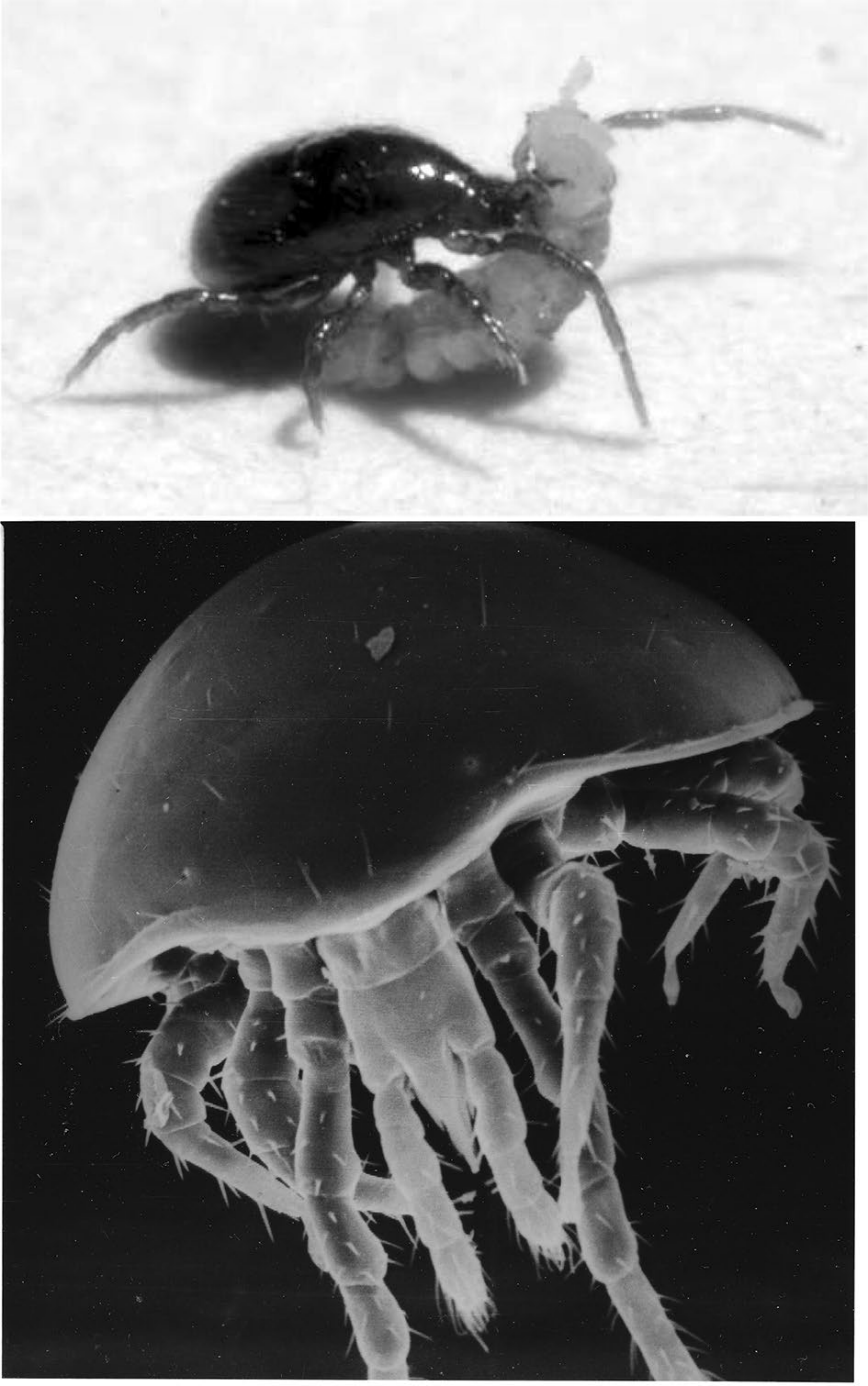
The gnathosoma of mesostigmatids can be airorhynchid or klinorhynchid in design with respect to general body orientation. *Upper* Mega-cephalic pergamasid feeding on collembola from a colour photograph © Philippe Legros, Villiers sur Marne, 94,350 France, November 26, 2011 with permission. Note typical gnathosoma orientation to attack prey forwards in front of the gamasine mesostigmatid and partial lifting of prey up off of substrate. *Lower* Scanning Electron microscope picture of typical micro-cephalic eviphidid mite. Note gnathosoma typically tucked under uropodoid idiosomal ‘carapace’ like a turtle or tortoise, pointing ventrally towards the substrate

Note that four out of the six uropodoids are also deemed micro-cephalic. The elongate chelicera and narrow gnathosoma in *Eviphis ostrinus*, *Trachytes aegrota*, *Urodiaspis tecta* and *Uropoda orbicularis* appear well suited for reaching into narrow crevices in search of prey items like happens in percine fish. As palps and leg sense organs are then of little use, the setal assembly on the tip of the fixed digit (Fig. 14) would be invaluable. It would be interesting to see if this is correlated with a difference in ‘mouth orientation’—i.e., if all their gnathosomas point down towards the substrate relative to the idiosoma versus straight ahead (the latter is $\equiv$ airorhynchy with respect to body axis) as in typical gamasines (Fig. 26) and if their chelal dentition has an extensive grasping surfaces to extract seized prey. More work on musculature following that of Alberti and Coons (1999) is needed to understand comparative gnathosomal flexure.

Finally note that no clear pattern could be seen amongst the meso-cephalics. These appear to be generally designed predators—their feeding being just morphologically categorised by their velocity ratio. Do other more subtle changes differentiate these species? A simple SEM study of appropriately preserved species as to whether their gnathosomal socket to idiosoma is: strictly antero-terminal (i.e., to allow picking at prey ‘dead-ahead’), strictly ventral (to allow picking prey below off of surfaces), or somewhere midway between the two (i.e., both styles inefficiently) would allow comparison to known feeding designs in perciforms (Carlson and Wainwright 2010). Of course for all mesostigmatids, the orientation of a gnathosoma in the idiosoma could also be adjusted by any flexibility around its socket. Gnathosomatic flexure muscles inserted on the ventral edge of the gnathosoma in anactinotrichids give the capability of the gnathosoma to be strongly deflected down and projected posteriorly (Fig. 5.16 in Evans 1979). For details of such musculature see Alberti and Coons (1999). So one would expect from the logic above, that veigaids would have gnathosomatic extensor muscles capable of raising their gnathosoma above the horizontal (like the predatory fish *Chromis atrilobata*), and that uropodoids could use their flexors to tuck their gnathosoma even further under the idiosoma than the default klinorhynchid position (much as the carnivorous benthic feeding fish *Zalembius rosaceus* which protrudes its jaws downwards (Aguilar-Medrano et al. 2011). This needs testing by further detailed observations and the comparative anatomy of gnathosomal musculatures.

At the same time as the gnathosoma flexing down, could there even be concerted flexure downwards of the middle cheliceral article with respect to the basal cheliceral article in *Heterozercon* on cheliceral retraction too (as the article flexure muscles unusually arise within the cheliceral retractor muscle group on the dorsum of the idiosoma in that species, Evans 1992)? Could everything gnathosomal be thus dragging material down and towards such a feeding mite? Do the fused basal and distal cheliceral articles in ameroseiids (Evans 1979), e.g., *Ameroseius*, *Brontispalaelaps*, work differently? What about the so-called ‘primitive’ holothyrids and opilioacarids? Uropodid klinorhynchy (see Fig. 2A in Athias-Binche 1977) suggests the mechanical digger action explanation for any ‘Rollplatte’ function rather than the above sabre-tooth action explanation. To what extent is mesostigmatid gnathosomal orientation correlated with their general idiosomal shape (like mouth orientation is with body shape in estuarine fish—Ruehl et al. 2011)?

Many specific questions remain. However, putting aside any uropodoid with extremely elongate chelicerae (cf. Fig. 25 Lower panel), using this cephalisation concept nicely partitions the general predatory mesostigmatid design into three broadly parallel curved classes.

Given that the heart in arachnids produces insufficient internal hydrostatic pressure to extend appendages (Manton 1958b), local idiosomal musculature must produce the compressive force onto the haemocoel to extend mesostigmatid chelicerae. Mites within an idiosomal size class possessing overly larger chelicerae (i.e., large CL for body size) should thus have muscular adaptations to suit. Larger muscles require stronger insertion points on the propodosma and a stiffer cuticle (at least anteriorly). This would suggest that amongst the ‘cutting/slicing’ mega-cephalics, the *Veigaia* spp. should be examined by acarine morphologists versus similar size parasitids and pergamasids for any such differential evidence. Are ventral sternal and dorsal propodosomal shields specially evolved for this?

### Can these results be related to Karg (1983)’s views on cheliceral dentition specialisms (as used by Walter and Ikonen 1989)?

Focusing on one attribute (velocity ratio, VR) raises the possibility of oversimplifying the interaction between mesostigmatid attributes and trophic evolution (Milne 2008). Dentition must play a part just as in vertebrates (see Hillaby 1980; Van Valkenburgh 2007). Disappointingly, there is little overlap between the species used by Buryn and Brandl (1992) and previous soil workers. Unfortunately, Walter and Ikonen (1989) do not appear to give a table of their 30 nematophagous species examined by SEM nor of the 10 looked at with phase-contrast microscopy, but by inference from their text and diagrams they appear to conclude the following assignations for some species:

- Type 1: with holding and crushing teeth: *Asca nesoica*, *Zygoseius furciger*, Eviphididae, Macrochelidae, Pachylaelapidae
- Type 2: with few large offset teeth opposed to a long saw-like sharp edge: *Lasioseius berlesei*, *Cheiroseius* nr. *mutilis*
- Type 3: with tweezer-like distal teeth opposed to small saw-like area: *Cheiroseius* sp.
- Type 4: with slender chelicerae with alternating rows of large and small teeth: *Rhodacarellus silesaicus*, *Gamasellodes vermivorax*, *Protogamasellus hibernicus*, *Protogamasellus mica*, *Dendrolaelaps zwoelferi*, *Dendrolaelaps strenskei*, *Dendrolaelaps procornutus*

How this fits with the observation that large wide teeth are found on wide jaws in other animals is not clear. Moreover, how this scheme fits with the five exemplars in Evans (1992) Fig. 6.5 on p.171 is not clear (although Fig. 6.5A Evans 1992—*Scarabaspis inexpectatus* matches Type 1). Which ‘Type’ *Paragamasus* sp. is (Fig. 6.5B p.171 Evans 1992) with its long slender digits and backwards facing teeth, or which ‘Type’ *Hypoaspis aculeifer* is with its strong digits and a row of small closely set teeth on the fixed digit (Fig. 6.5C p.171 Evans 1992) is not clear. Moreover, Karg (1983) illustrates two other distinct forms (Fig. 6.5D and 6.5E in Evans 1992). These are typified by the nematophagous *Lasioseius berlesei* (Type D, with a few large teeth on the moveable digit opposing a closely set row of relatively smaller teeth on the fixed digit allocated to Type 2 above) and the nematophagous *Cheiroseiulus reniformis* (Type E, with distal teeth on the moveable digit opposed to a restricted area of saw-like teeth on the fixed digit—is this Type 2 as well?). Clarity of typology is needed.

In the above four types, confusingly the known arthropod predator and Collembola specialist feeder *Veigaia pusilla* (Hurlbutt 1968; Walter et al. 1988) is placed into Type 3 nematophagy even though it will only eat nematodes if starved. Type 4 is deemed to be the general nematode-arthropod predatory type that feeds upon both. The results in this review for *Rhodacarellus sileciacus*—the only taxon in common—points to its chelal design being that of a crushing/mangling worm-like prey feeder. Thus it is not clear why Type 4 dentition is of importance to it—lots of small teeth facilitate gripping slippery prey (cf. jaws of shrews, crocodiles, etc.). Is it that rhodacarids focus on say nematodes with an excessive slipperiness? Mucus is known as a defence against nematodes (Yu et al. 2019) as well as itself providing a bacterial growth medium (Moens et al. 2005). The calculated velocity ratio for *Macrocheles montanus* in Table 3 suggests it is a crusher of food offering some consilience to Karg (1983)’s scheme. However, species in Type 2 do feed on nematodes and also feed upon small arthropods, with a number of them feeding upon fungi as well (see refs in Walter and Ikonen 1989). Accordingly, the sensitivity (the proportion of actual positives that are correctly identified as such) and the specificity (the proportion of actual negatives that are correctly identified as such) of Karg (1983)’s approach is called into question. Better to first look at overall chelal design (VR) before concluding functional feeding type based upon detailed dentition. In that way Buryn and Brandl (1992) were indirectly on the right track.

A standard nomenclature like pluridentate, fissidentate and unidentate (as used for argiopid spider chelae—Comstock 1948) and an agreed ontology is needed. This needs to be posed in terms of how teeth occlude and function (see Freeman 1992) in prey ‘sawing’, mastication and trituration as used in other animals.

### Is taxonomic position a confounding factor?

Soil ecologists have posited that invertebrate function in systems can be predicted at a low level of taxonomic resolution (Walter and Ikonen 1989). Yet, evolution can be capricious, as Van Valkenburgh (2007) says “This tendency towards the iteration of similar forms results in numerous homoplasies that frustrate systematists but entrance functional morphologists”. For sure, the observations above suggest that uropodines might just scale differently to gamasine mesostigmatids? Could other taxonomic groups show taxonomically distinct series too? After all long ago (Manton 1958a), it was shown that the apparently non-adaptive features used in diagnostic classification do have a functional significance in arthropods. Moreover there is definitely a different acarine taxonomic correlation across ephemeral versus stable soil-litter habitats (Walter and Proctor 2013). To that end, there has been widespread success in summarising mite habitat habits using family assignation (e.g., Krantz 1971). Do such functional simplifications apply to mite genera as much as they do to birds (Grant 1986)? At what level of classification are there clear patterns in cheliceral design?

**Fig. 27:**
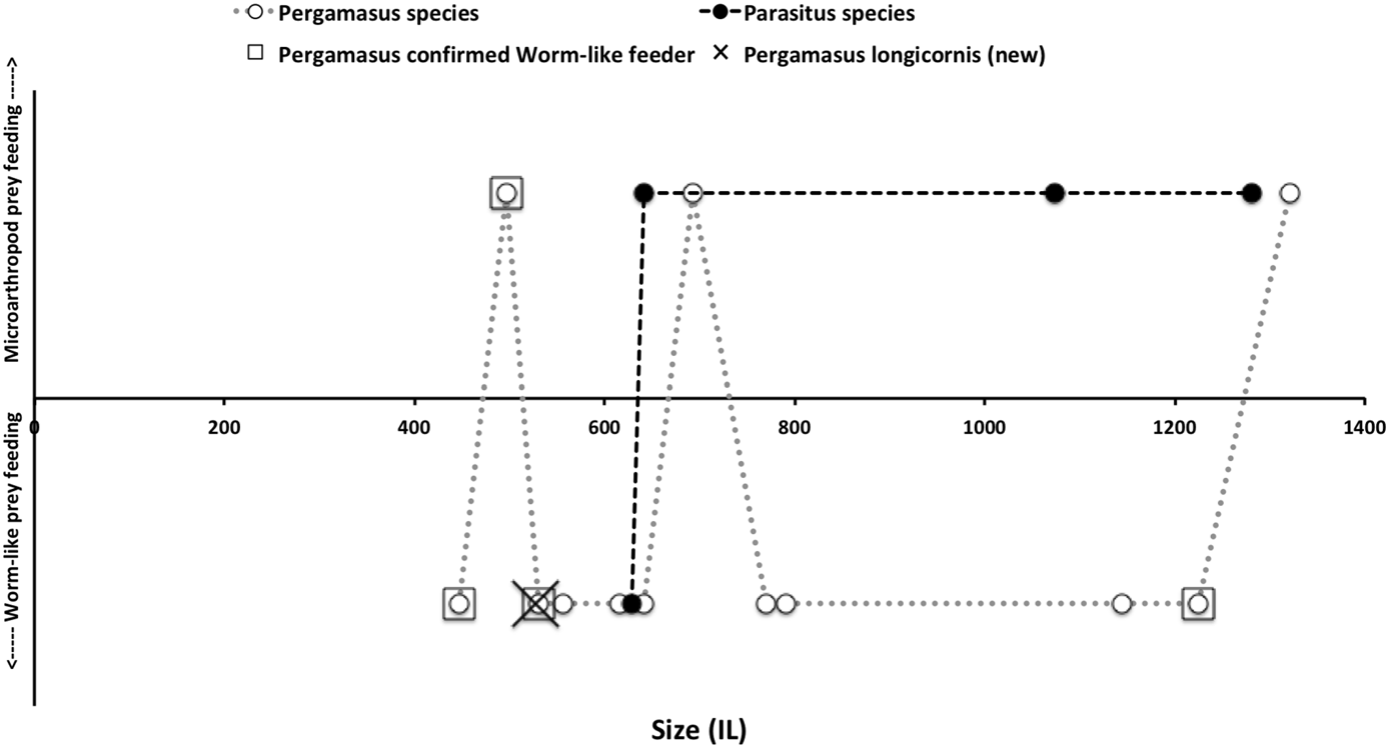
Trajectories for the model predictions for the four *Parasitus* species (black circles plus black dashed line) and twelve *Pergamasus* species (white open circles plus grey dotted line) as body size (IL) increases, based upon *glm* model in Table 9, threshold $p=0.475\equiv 0.276$ cut in Velocity ratio VR). Actual feeding type (open squares)—note broad agreement in general and almost perfect fit to a common fate for species of a genus above general size class (450–469 μm body size). Extra sample male *Pergamasus longicornis* (Meathop Woods, Cumbria, UK) marked as X (observed $\text {lL}= 527.2$ μm, velocity ratio $= 0.193 \equiv$ predicted worm-like prey feeding habit). Note good agreement with adult *Parasitus* spp. kill-style habits in Table 11 from Hyatt (1980)

Plotting the trajectory of predicted prey type (arising from the *glm* model fitted chelal velocity ratio) against body size (IL) for species from the genus *Parasitus* (four species available), and separately for species from the genus *Pergamasus* (12 species available), shows evidence of different fates (Fig. 27).

Traditionally, pergamasines (along with the Veigaiidae and Rhodacaridae) form the major predators of forest and grassland soils in the Palaearctic (Evans 1992). Their deuteronymphs are never phoretic (i.e., their environment is almost certainly not temporary). Therefore it would be tempting to conclude that these should have (at least) a micro-arthropod feeding habit. However, from a body size increasing from around the 500 $\mu m$ mark (recall the generalist 450–649 $\mu m$ IL bin above), *Pergamasus* spp. essentially maintain a design suitable for feeding on and crushing worm-like prey. This backs-up the observations of high nematode feeding rates and the ease of maintaining *Pergamasus* sp. (and *Paragamasus* sp.) by Walter and Ikonen (1989). The argument in Walter and Ikonen (1989) that the chelicerae in the genus *Pergamasus*—claimed as collembola-mite feeding specialists by Karg (1983)—should (also) be efficient structures for attacking nematodes is supported. It does not support Sadar and Murphy (1987)’s assignation of *Pergamasus* spp. as micro-arthropod predators. It better matches Pugh and King (1985) who found that *Pergamasus longicornis* fed upon wounded or carrion acarines. Luxton (1966) found it to be the only mite on salt-marshes that could attack oribatids. Could it be Tyrannosaur-like? For sure pergamasids have some of the largest F2AV crunch force values in Table 3 (three out of the top seven). Notonectids which feed upon large prey have capture mechanisms with high leverage coefficients capable of generating slow powerful movement against struggles (Goodwin 1976). Another way is to have a large adductive force (Barghusen 1972). *Pergamasus (P.) diversus* examined by Sadar and Murphy (1987) is not included in Buryn and Brandl (1992)’s species—but its quoted total size of about 820 *mu*m long suggests (Fig. 27) that trophic design-wise it may be more like the slightly smaller *Pergamasus runcatellus*. Even *Pergamasus runciger* with a chelal velocity ratio of 0.270 (Table 3) equivalent to a probability of being a worm-like feeder of 0.477 points just to the equivocation in the centre of the ROC curve (Fig. 21). Here other subtle changes in trophic features may determine prey handling efficiency. Of course, a high-speed slicing chela may be suitable for prey mashing if there is a high enough static adductive force F1. A check of the likely feeding prediction from their velocity ratio for other *Paragamasus* species in persistent and temporary habitats here would help clarify this area in future work and improve the classifier whose statistical leverage is determined by species at the edges of the morphospace.

**Table 11:** Parasitine validation

Species	Stage	MD ratio	VR	VR ex Tables 3,8	Proposed feeding from validation cohort
*Cornigamasus lunaris* $\dag$	DN	0.349	*0.242*	0.202	Micro-arthropod prey
	$\male$	0.314	*0.218*		Micro-arthropod prey
	$\female$	0.413	0.203		Micro-arthropod prey
*Eugamasus berlesei*	DN	0.318	0.216		Micro-arthropod prey
	$\male$	0.322	*0.223*		Micro-arthropod prey
	$\female$	0.349	0.214	0.317	Micro-arthropod prey*
*Eugamasus cavernicola*	$\female$	0.440	0.264	0.299	Micro-arthropod prey*
*Eugamasus crassitarsis*	$\male$	0.356	0.185		Micro-arthropod prey
	$\female$	0.394	0.368		Worm-like prey
*Eugamasus magnus*	$\male$	0.398	*0.276*		Worm-like prey
*Gamasodes bispinosus*	DN	0.413	*0.287*		Worm-like prey
	$\male$	0.377	*0.261*		Micro-arthropod prey
	$\female$	0.364	*0.252*		Micro-arthropod prey
*Gamasodes fimbriatus*	$\male$	0.319	*0.221*		Micro-arthropod prey
	$\female$	0.395	0.289		Worm-like prey
*Gamasodes spiniger*	DN	0.482	*0.335*		Worm-like prey
	$\male$	0.318	*0.221*		Micro-arthropod prey
	$\female$	0.392	0.215		Micro-arthropod prey
*Parasitellus crinitus*	DN	0.403	0.233		Micro-arthropod prey
	$\male$	0.425	*0.295*		Worm-like prey
	$\female$	0.359	0.225		Micro-arthropod prey
*Parasitellus fucorum* $\ddag$	DN	0.459	*0.318*	0.334	Worm-like prey
	$\male$	0.299	*0.207*		Micro-arthropod prey
	$\female$	0.277	0.230		Micro-arthropod prey
*Parasitellus ignotus*	DN	0.436	0.271		Micro-arthropod prey
	$\male$	0.371	0.351		Worm-like prey
	$\female$	0.356	0.336		Worm-like prey
*Parasitellus talparum*	DN	0.456	0.283		Worm-like prey
	$\male$	0.333	0.381		Worm-like prey
	$\female$	0.344	0.297		Worm-like prey
*Parasitus beta*	DN	0.407	*0.282*		Worm-like prey
	$\male$	0.154	*0.107*		Micro-arthropod prey
	$\female$	0.392	0.295	0.340	Worm-like prey
*Parasitus coleoptratorum*	DN	0.393	0.265	0.276	Micro-arthropod prey
	$\male$	0.367	*0.255*		Micro-arthropod prey
	$\female$	0.351	0.259		Micro-arthropod prey
*Parasitus consanguineous*	DN	0.388	0.312		Worm-like prey
	$\male$	0.293	*0.203*		Micro-arthropod prey
	$\female$	0.366	0.283		Worm-like prey
*Parasitus copridis*	DN	0.336	0.306		Worm-like prey
	$\male$	0.365	*0.253*		Micro-arthropod prey
	$\female$	0.307	0.258		Micro-arthropod prey
*Parasitus evertsi*	$\male$	0.330	*0.229*		Micro-arthropod prey
	$\female$	0.308	0.287		Worm-like prey
*Parasitus fimetorum*	Larva	0.466	*0.323*		Worm-like prey
	PN	0.476	*0.330*		Worm-like prey
	DN	0.369	*0.256*		Micro-arthropod prey
	$\male$	0.163	*0.113*		Micro-arthropod prey
	$\female$	0.348	*0.241*		Micro-arthropod prey
*Parasitus hyalinus*	$\female$	0.403	*0.280*		Worm-like prey
*Parasitus insignis*	DN	0.326	0.260		Micro-arthropod prey
	$\male$	0.296	*0.205*		Micro-arthropod prey
	$\female$	0.330	0.237		Micro-arthropod prey
*Parasitus kempersi*	DN	0.357	0.279		Worm-like prey
	$\male$	0.343	*0.238*		Micro-arthropod prey
	$\female$	0.336	0.216		Micro-arthropod prey
*Parasitus loricatus*	DN	0.381	*0.264*		Micro-arthropod prey
	$\male$	0.435	*0.301*		Worm-like prey
	$\female$	0.295	0.170		Micro-arthropod prey
*Parasitus mustelarum*	DN	0.401	0.267		Micro-arthropod prey
	$\male$	0.312	*0.217*		Micro-arthropod prey
	$\female$	0.540	0.281		Worm-like prey

Measurement using ImageJ 1.51s (http://imagej.nih.gov/ij) on line drawings in Hyatt (1980). MD ratio = ratio of Moveable digit length (MDL) to Moveable digit height (not including spermatodactyl in males) and Velocity ratio ($VR=\frac{L1}{L2}$) when condyle and likely levator tendon location were estimable. Velocity ratio (VR) in italics predicted from overall regression of measured MD ratio to measured VR (slope = 0.6937). Threshold cut-off VR = 0.276 for proposed feeding habit. *Conflict in prediction. $\dag$Listed as *Parasitus lunaris* elsewhere. $\ddag$Listed as *Parasitus fucorum* elsewhere

**Table 12:** Parasitine validation (continued)

Species	Stage	MD ratio	VR	VR ex Tables 3,8	Proposed feeding from validation cohort
*Poecilochirus austroasiaticus*	DN	0.467	*0.324*		Worm-like prey
	$\male$	0.421	*0.292*		Worm-like prey
	$\female$	0.424	*0.294*		Worm-like prey
*Poecilochirus carabi*	PN	0.349	*0.242*		Micro-arthropod prey
	DN	0.355	*0.246*		Micro-arthropod prey
	$\male$	0.377	*0.262*		Micro-arthropod prey
	$\female$	0.345	0.215		Micro-arthropod prey
*Poecilochirus davydovae*	DN	0.453	*0.315*		Worm-like prey
	$\male$	0.329	*0.229*		Micro-arthropod prey
	$\female$	0.406	*0.281*		Worm-like prey
*Poecilochirus subterraneus*	DN	0.406	0.264		Micro-arthropod prey
*Porrhostaspis lunulata*	DN	0.335	0.342		Worm-like prey
	$\male$	0.270	*0.187*		Micro-arthropod prey
	$\female$	0.303	*0.210*	0.227	Micro-arthropod prey
*Trachygamasus ambulacralis*	DN	0.520	*0.361*		Worm-like prey
	$\male$	0.419	*0.290*		Worm-like prey
	$\female$	0.391	*0.272*		Micro-arthropod prey
*Vulgarogamasus burchanensis*	$\male$	0.276	*0.191*		Micro-arthropod prey
	$\female$	0.353	0.213		Micro-arthropod prey
*Vulgarogamasus immanis*	DN	0.295	0.170		Micro-arthropod prey
	$\male$	0.244	*0.169*		Micro-arthropod prey
	$\female$	0.284	*0.197*		Micro-arthropod prey
*Vulgarogamasus kraepelini*	DN	0.348	0.217		Micro-arthropod prey
	$\male$	0.401	0.207		Micro-arthropod prey
	$\female$	0.341	0.260		Micro-arthropod prey
*Vulgarogamasus oudemansi*	DN	0.390	0.263		Micro-arthropod prey
	$\male$	0.389	0.386		Worm-like prey
	$\female$	0.355	0.196		Micro-arthropod prey
*Vulgarogamasus remberti*	DN	0.412	0.189		Micro-arthropod prey
	$\male$	0.435	*0.302*		Worm-like prey
	$\female$	0.342	0.192		Micro-arthropod prey
*Vulgarogamasus trouessarti*	DN	0.372	0.206		Micro-arthropod prey
	$\male$	0.305	*0.212*		Micro-arthropod prey

Measurement using ImageJ 1.51s (http://imagej.nih.gov/ij) on line drawings in Hyatt (1980). MD ratio = ratio of Moveable digit length (MDL) to Moveable digit height (not including spermatodactyl in males) and Velocity ratio ($VR=\frac{L1}{L2}$) when condyle and likely levator tendon location were estimable. Velocity ratio (VR) in italics predicted from overall regression of measured MD ratio to measured VR (slope = 0.6937). Threshold cut-off VR = 0.276 for proposed feeding habit. *Conflict in prediction. $\dag$Listed as *Parasitus lunaris* elsewhere. $\ddag$Listed as *Parasitus fucorum* elsewhere

In contrast, *Parasitus* spp. appear to persist with a design suitable for feeding by cutting and slicing micro-arthropods at any body scaling. This fits with Pugh and King (1985) who finds *Parasitus kempersi* feeding on adult/fresh carrion diptera, dipteran larvae, other gamasids, live springtails and oligochaetes. It does not agree with Ito (1971)’s observation of nematode eating by manure-inhabiting parasitids. Parasitines occur commonly in temporary accumulations of organic debris like compost, manure, tidal debris and insect/small mammal nests (Evans 1992). Such drying-out conditions may not be equable for nematodes to prosper as a persistent food source. Validation using data abstracted from illustrations in Hyatt (1980) (Tables 11, 12), shows that only two out of seven species appear misclassified by the proposed algorithm herein. Taking the females, 21 out of 31 species are predicted to have a tissue-slicing micro-arthropod feeding habit—a large proportion! however, within species, 20 out of 31 of the parasitine taxa examined show a different prediction of feeding type across the illustrated developmental stages (four taxa only had one developmental stage offered). This suggests further subtlety in evolutionary design to perhaps avoid inter-generational trophic competition. Unfortunately Hyatt (1980), whilst taxonomically describing the species and their shields rigorously, does not provide a standard idiosomal length for each parasitine with which to see if this switch is related to overall body size (viz. Fig. 27). Nor does Hyatt (1980) detail any levator tendon strengthening. Such awaits further morphometric work, as does examining all the species of the parasitid genus *Schizosthetus* known from elsewhere in the world (Al-Atawi et al. 2002).

Trophism thus appears to generally match genealogy. Karg (1983) was partially right—genus (and perhaps sub-family but not necessarily family) level mesostigmatid taxa have become feeding specialists with those specialisms reflected in the structure of their mouthparts.

### Validating the importance of taxonomic position

**Fig. 28:**
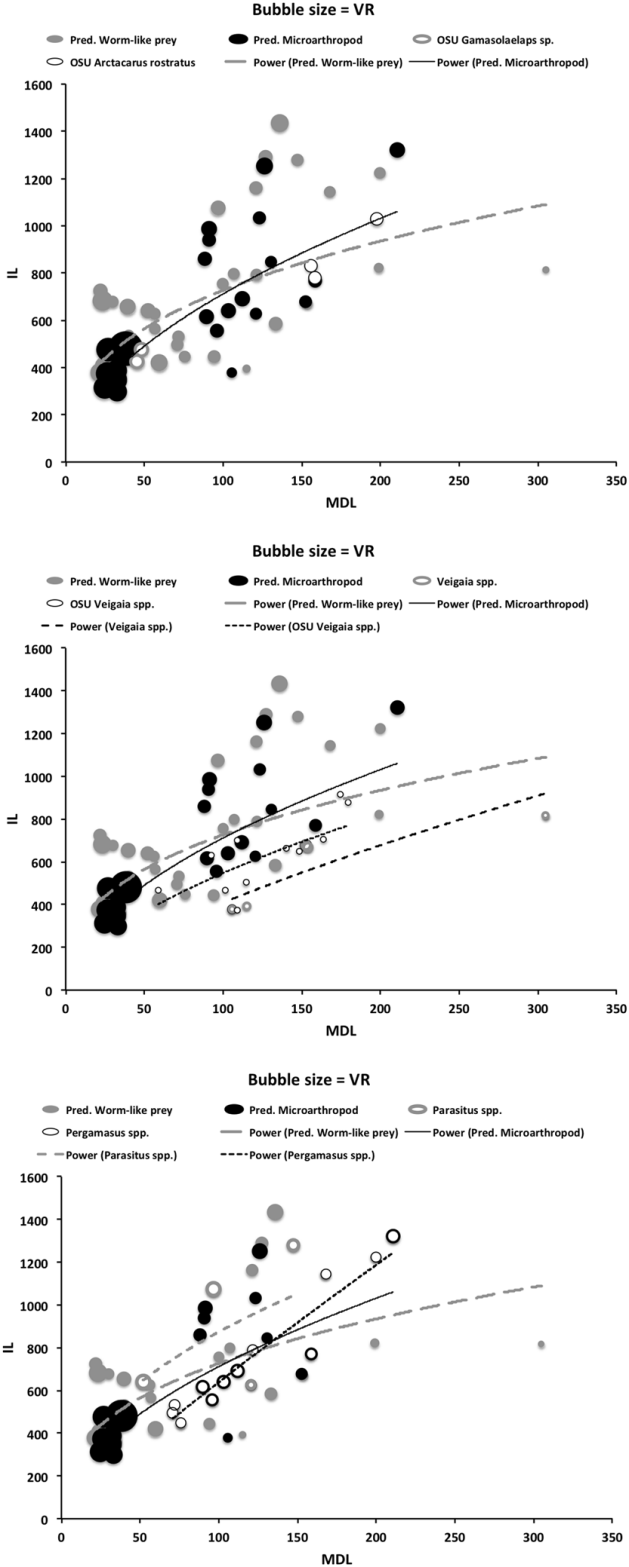
Large gape species diverge. Specimens from Acarology Laboratory, Ohio State University museum slide collection (Table 7). *Upper* Plot of gape (MDL) versus body size (IL) in $\,\mu \text {m}$. Note regression lines for both predicted microarthropod prey feeder and predicted worm-like prey feeding are similar, but diverge for predatory mites approximately $>500\,\mu \text {m}$ size. Bubble size for *Arctacarus rostratus* and *Gamasolaelaps* sp. arbitrary (L1 not measurable). *Middle* Plot of gape (MDL) versus body size (IL) in $\,\mu \text {m}$. Note added regression lines for both *Veigaia* spp in this study and the extra *Veigaia* spp. from OSU museum slides approximate the slope of the predicted micro-arthropod prey feeding species relationship showing consilience. Bubble size for OSU *Veigaia* spp. arbitrary (L1 not measurable). *Lower* Plot of gape (MDL) versus body size (IL) in $\,\mu \text {m}$ with added regression lines for *Parasitus* spp. and *Pergamasus* spp. from Table 3). Note that the slope of regression lines for both *Parasitus* spp in this study and *Pergamasus* spp. which exceed the slope of the predicted micro-arthropod prey feeding species relationship suggesting possible taxonomically specific scaling relations

The large gape *Veigaia* species are another series for possible examination—plotting the moveable digit length and idiosomal index available from a variety of large gape species from the Ohio State University Acarology Laboratory museum slide collection is shown in Fig. 28 (data in Table 7). It was not possible to unequivocally measure L1 on these permanently mounted specimens unfortunately. Just these two parameters (IL, MDL) can neither definitively predict the chelal velocity ratio (Fig. 28*Upper*), nor the chelal crunch force (*results not shown*) overall. However, it is clear that all *Veigaia* genus OSU exemplars occupy a defined zone of morphological design typified in the training and test data set as modest size, disproportionately large gape mites who exemplify a modest velocity ratio (look at bubble size—i.e., they are likely to be fast-snapping micro-arthropod slicing feeders). Examining the slope of the regression lines (Fig. 28*Middle*), suggests that at very large sizes such big gape mites are definitely more likely to be micro-arthropod predators than worm-like prey feeders (as possibly is also true for a few *Pergamasus* spp. too). Moreover, *Veigaia* species manifestly scale in a different way than say *Parasitus* spp. or *Pergamasus* spp. (Fig. 28*Lower*). This confirms again a genus-level taxonomic confounder to mesostigmatid ecomorphology. The remaining firm UK species from Evans (1955) which also encompass short and weakly developed chelicerae species: *Veigaia bouveri*, *Veigaia kochi*, *Veigaia transisalae*, *V. serrata*; as well as those in North America (beyond just those listed within Table 2 in Hurlbutt 1965) remain to be examined.

It would useful to study other within-genus series in detail in further work on other mesostigmatid families: perhaps the *Asca* species investigated in the field by Hurlbutt (1968); the large genus *Pachylaelaps* (Krantz 1978); or more eviphidids (considered nematophagous by Evans 1979, but 50% of European genera are phoretic insecticoles—Mašán and Halliday 2010), or to examine various trachytoids or protodinychoids. Amongst uropodines, *Clivosurella* and *Sumatrella* might be interesting as Konstchán (2016) and Konstchán (2015) respectively, appear to illustrate them with a Rollplatte. One possible particularly fruitful area would be the genera comprising the 400 or so species in the Macrochelidae known to predate nematodes (Ito 1971), oligochaetes, enchytraeids and arthropod eggs/early instars (Leitner 1946; Evans 1979; Rodriguez et al. 1962; Krantz 1998). Pachylaelapids, with their extraordinarily wide ecological and behavioral diversity (Mašán et al. 2016) do similar things and could be examined in parallel. Do macrochelid cheliceral design form a graded series of evermore powerful ‘cracking’ adaptations (Ferretti 2007)? Pugh and King (1985) lists *Macrocheles superbus* as a feeder of live, wounded and fresh carrion springtails including eggs. *Macrocheles subbadius* feeds upon carrion, nematodes and the eggs of other Acari and insects in salt marshes (Luxton 1966). Walter and Ikonen (1989) recounts that *Macrocheles schaferi* is an equally proficient predator of arthropods and nematodes, but Sadar and Murphy (1987) claims that *Macrocheles glaber* has cheliceral morphological features related to preying upon enchytraeid worms and fly larvae. For sure, *Macrocheles glaber* will eat mushroom fly larvae (Wen et al. 2019). Does its cheliceral design have commonalities to that of *Stratiolaelaps scimitus* known to successfully develop on fungus gnat larvae and enchytraeids (Cabrera et al. 2005)? The description of feeding by Oliver and Krantz (1963) of *Macrocheles rodriguezi* sounds very pergamasid-like. De Azevedo (2017) offers much biological insight into this diverse group. Kamaruzaman et al. (2018) recounts the ecological succession of macrochelids on rotting corpses—do the chelal velocity ratio of species present over time reveal a consilient pattern of resource use? Some macrochelids are ovovivparous (Marquardt et al. 2015), are there any cheliceral design characteristics in these species to facilitate egg handling? Could cheliceral morphology be related to possible group feeding in macrochelids (see above)? As *Hypoaspis aculeifer* is known to be eusocial (Usher and Davis 1983) could any macrochelid conclusions be confirmed by comparing them to the large number of *Hypoaspis* spp. in the Laelapidae family? How do the 120 species or so of *Gaeolaelaps* laelapids compare (Beaulieu 2009; Kazemi et al. 2014; Navarro-Campos et al. 2016; Kazemi 2020)? Are the Iphiopsididae (Uppstrom and Klompen 2005) distinct in trophic design to other laelapids? An interesting major morphological project for an acarologist beckons!

Do cheliceral characters explain competition and coexistence amongst tropical rhinoseids (Colwell 1973)? Given that the host association pattern of *Euryparasitus* species appears to fit better with ecological rather than with host specificity (Hagele et al. 2005), then examining the chelicerae of this group could be illuminating. Do the various uropodine species found in badger setts (Kurek et al. 2020) successfully coexist by having different cheliceral designs? Do myrmecophilous genera like *Sphaerolaelaps* (Evans 1979) show distinct features in their chelicerae? What can be learnt from other mesostigmatid ant symbionts (Lachaud et al. 2016; Pérez-Lachaud et al. 2019)?

The bark beetle biome is of current interest (Knee et al. 2012)—can reach, gape, velocity ratio, chelal crunch force etc., explain the co-existence of mesostigmatids in *Ips typographus* bark beetle galleries—Khaustov et al. 2018? One wonders what the situation is in: not just more fungi / saprophytic mite/pollen feeding ascids (Lindquist 1963; Evans 1979); laelapids; humus-living sejoids (Evans 1979); thinozerconoids; polyaspidoids and “tortoise mite” uropodids (Krantz 1978) which all should show high velocity ratio values; but also, non-gamasines/non-uropodine monogynapsids like *Liroaspis* spp.; other diarthropalloids; or trigynaspids like the antennophorines (e.g., *Antennophorus* spp., *Celaenopsis balinus* (see Evans 1979), *Euzercon* spp. (see Krantz 1978), *Fedrizzia* spp. (see Seeman 2007), and *Megisthanus* spp. (see Gorirossi and Wharton 1953, Butler and Hunter 1968; Seeman 2017, 2019)). Pioneer work was done by Gorirossi (1955b) on the gnathosoma of *Euzercon latus* and *Passalacarus sylvestris* but what about the paramegistids (Kim and Klompen 2002)? What about the serrate chela cercomegistines like *Cercomegistus* spp. (see Krantz 1971) or the nematophagous *Cercoleipus coelonotus* which will eat digamasellids too (Krantz 1978)? What are their chelal velocity ratios? Cercomegistines are known to be aggressive predators of small invertebrates and ingest fluids only (Walter and Proctor 1998). What about the tree stump/rotting wood inhabitant *Microsejus truncicola*—the only British microgyniid (Evans 1979)? In fact, where do other nematophagous digamasellids (Krantz 1978) than *Dendrolaelaps foveolatus* sit on the mechanical spectrum? A *Gamasellus* species is included in Perdomo et al. (2012) with a leverage (their MH/ML $\approx$ VR) of $<0.3$ is this typical? What about fungivorous *Digamasellus* spp. (Evans 1979) and other *Dendrolaelaps* spp. (Wišniewski and Hirschmann 1989)?

Oligogamasids show prey specificity (Lee 1974) to Collembola versus mites—do their chelal parameters match this? Karg (1961) (and others—see Walter and Oliver 1989) record *Geolaelaps aculeifer* as an example of a polyphagous arthropod predator. What velocity ratio and estimated chelal adductive force do *Geolaelaps* spp. in general show which are known to be aggressive predators of nematodes and arthropods attacking prey that are many times their size? Do they eventually chew through arthropod soft cuticle spots (as Walter and Oliver 1989 describe) or does their velocity ratio suggest that they in fact slice or saw their way through? What do the chelae of fungal/algal/spore/plant fragment feeding ameroseiids (*Kleemania* spp., *Epicriopsis* spp.; Evans 1979) look like? Do they all have ’crushing’ type velocity ratios as for *Ameroseius* sp. in Table 3 like astigmatids? Do pollen and nectar-feeding ameroseiids (Mašán 2017) have a different design? Can cheliceral parameters shed light on what epicriids, zerconids (Evans 1979; Ujvári 2011) and heterozerconids (Klompen et al. 2013) are likely to feed on? The prediction for *Zercon peliatus* in Table 8 suggests tough food needing a high velocity ratio, yet zerconid species are considered oligophagous predators (Evans 1992).

*Philippinozercon makilingensis* females found in millipede frass and litter, never from millipedes, have very long moveable digits (Gerdeman et al. 2018)—what is that indicating regarding their diet? Dramatic changes in cheliceral form can occur from larva to adult in *Narceoheterozercon ohioensis* (Gerdeman and Klompen 2003), what is that suggesting trophically? In this broad group, is there evidence of chelal isometry or developmental allometry as in the fiddler crab genus *Uca* (Rosenberg 2002)? Some discozerconids (Seeman and Baker 2013) also have elongate cheliceral digits—what might they be adapted to feed upon? Are certain values for cheliceral parameters accompanied by differences in the size of the anal orifice known to be enlarged in particulate feeding ichthyostomatogasterid sejines like *Asternolaelaps* spp. (Evans 1954b; Athias-Henriot 1972; Walter and Proctor 1998)? How does this compare to other sejines like *Sejus* (Lekveishvili and Klompen 2004b, 2006; Trach and Toistikov 2016) and *Uropodella* who are aggressive predators of small invertebrates and ingest fluids only (Walter and Proctor 1998)? Much straightforward simple morphological work within this mechanical model is left for acarologists to do.

### What might comparison to vertebrate carnivore mandibles suggest?

**Fig. 29:**
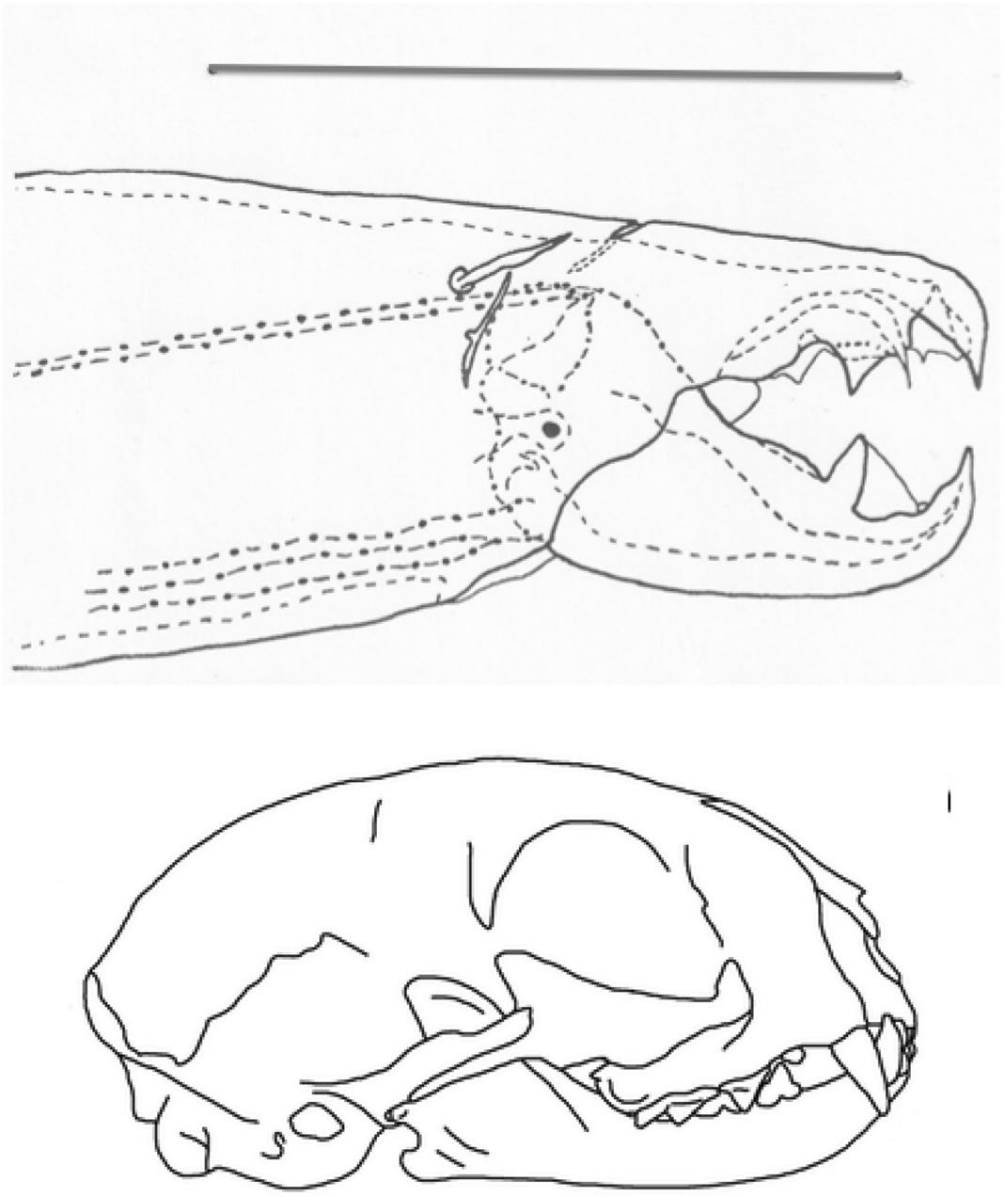
Obligate nematode feeding uropodids have robust slicing chelae. *Upper* *Alliphis halleri* $\female$. Grey bar is $50\,\mu \text {m}$. Levator and depressor tendons marked as dot and dashes. Dot as condyle position. *Lower* Cat skull from Veitschegger et al. (2018) under Creative Commons Attribution 4.0 International (CC BY 4.0). Note brachycephalic cat skull contrasts with dolichocephalic dog skull in Fig. 31. Velocity ratio (Radinsky 1981) estimated from jaw length moment arm (JL)/temporalis moment arm (MAT) in diagram: $\text {Felid} = 0.254$

**Fig. 30:**
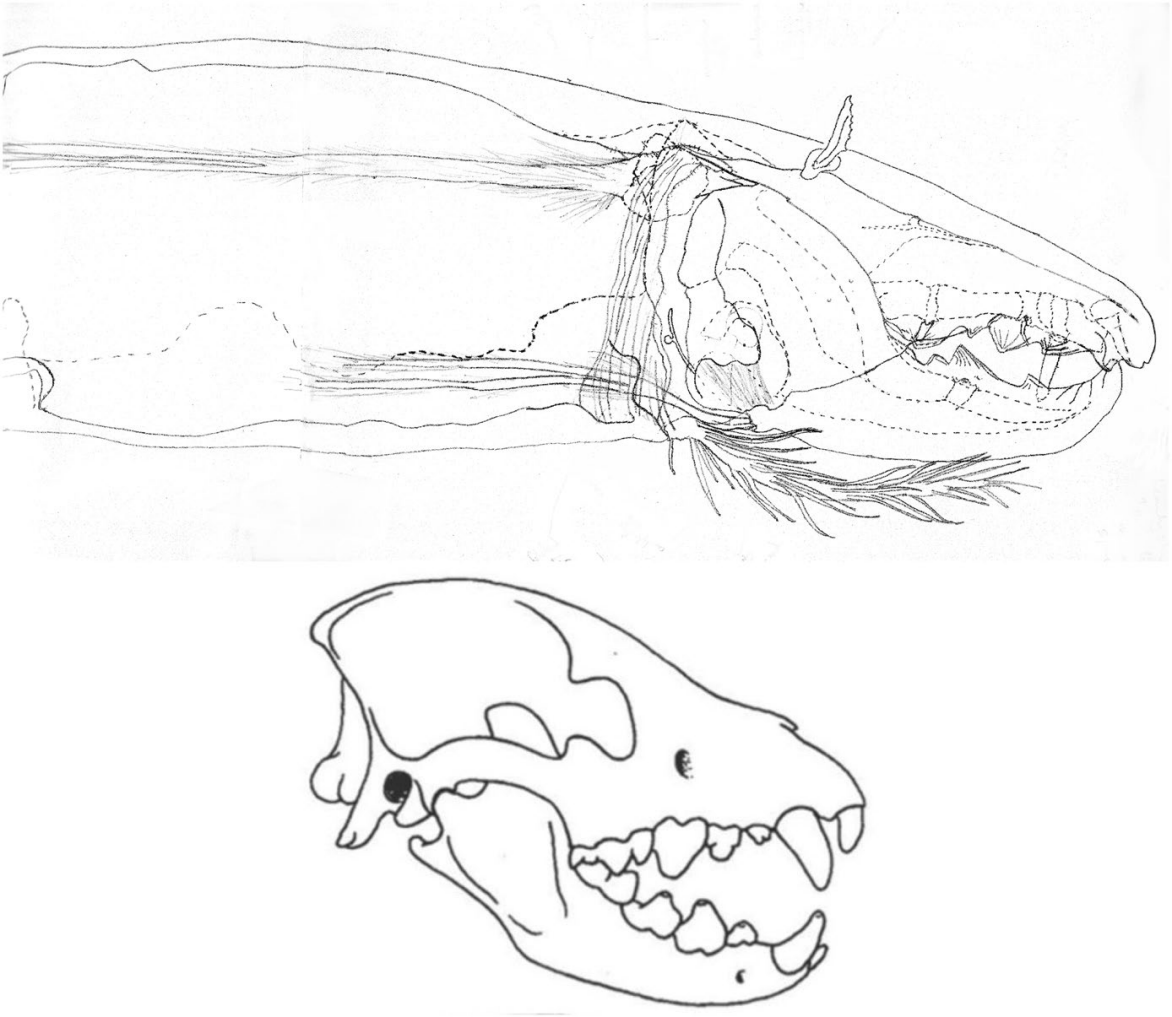
Robust chela in *Glyphtholaspis confusa* $\female$. *Upper* Levator and depressor tendons marked as dot and dashes. Dot as condyle position. Note shortened massive deep rigid digits ($\text {VR} = 0.406$ Table 3). Cheliceral shaft strengthened like sagittal crest and dome like hyena skull profile suitable to dissipate compressive stresses. *Lower* Bone-crushing Spotted Hyena skull (*Crocuta crocuta*) from Van Valkenburgh (2007) © Blaire Van Valkenburgh 2007 with permission. Velocity ratio (Radinsky 1981) estimated from jaw length moment arm (JL)/temporalis moment arm (MAT) in diagram: $\text {Hyaenid} = 0.241$. Note important masseter muscle omitted.

The vertebrate mammal order Carnivora is divided into two suborders: Feliformia (“cat-like”); versus Suborder Caniformia (“dog-like”). Despite both showing classic meat-slicing carnassial teeth approximately half way along their mandible, the species in the former are generally typified by a shorter rounder snout on a broader face, the latter are typified by a more longer snout on a more pointed head. Mandible design—though varying in trophic dentition—follows similarly. It is tempting to suggest that, amongst the extra mite species used in this study, the moveable digit of:

- *Alliphis halleri* (Fig. 29) is analogous to that of a felid cat mandible being foreshortened (and having lost its teeth equivalent to incisors/canines). This effectively differential allometry within a chela is already known in lobsters (Farmer 1974). In other words this mite is highly adapted for slicing prey tissue but with no need to pounce on and grip prey. Whether *Alliphis* as a genus shows the variety of known feline morphologies (Morales and Giannini 2010; Sicuro 2011) awaits confirmation.
- *Glyphtholaspis confusa* is analogous to that of a hyena mandible (Fig. 30). That is with distal cheliceral dentition like that of broad gripping incisors and canines, but with the beginnings of a blunt crushing ‘molar’-like dentition (as in snail-crushing lizards; Dalrymple 1979a) proximally behind the central slicing ’carnassial shaped’ cheliceral teeth. Its velocity ratio at 0.406 is close to the 0.428 of the crusher chela in *Menippe mercenaria* (Schenk and Wainwright 2001). This brachyuran crab feeds almost exclusively on hard prey including bivalves, gastropods and hermit crabs. Just as hyaenid jaws are adapted to crush bones (Van Valkenburgh 2007), *G. confusa* probably specialises on very hard arthropod body parts/eggs/pupal cases etc. This is consilient with it not eating nematodes (Ito 1971). In this study it had the second largest adductive force (F2AV) behind the even bigger ‘raptor-like’ *Pergamasus septentrionalis*—does the fixed digit strengthening (Fig. 30) dissipate masticatory stress like the frontal dome of hyaenids (Tseng and Stynder 2011)? Are macrochelids like this too? Could this family contain strong broad snout like chelicerae useful to flip over debris in order to consume newly discovered prey like in logperch fishes (Carlson and Wainwright 2010)?
- *Parasitus coleoptratorum*, *Parasitus lunaris*, *Parasitus lunaris*, *Polyaspis* n.sp. and *Veigaia nemorensis* is analogous to a general purpose hunting-dog mandible, including an elongate jaw and interlocking digit tips (see Fig. 13) equivalent to canid canines in function.

**Fig. 31:**
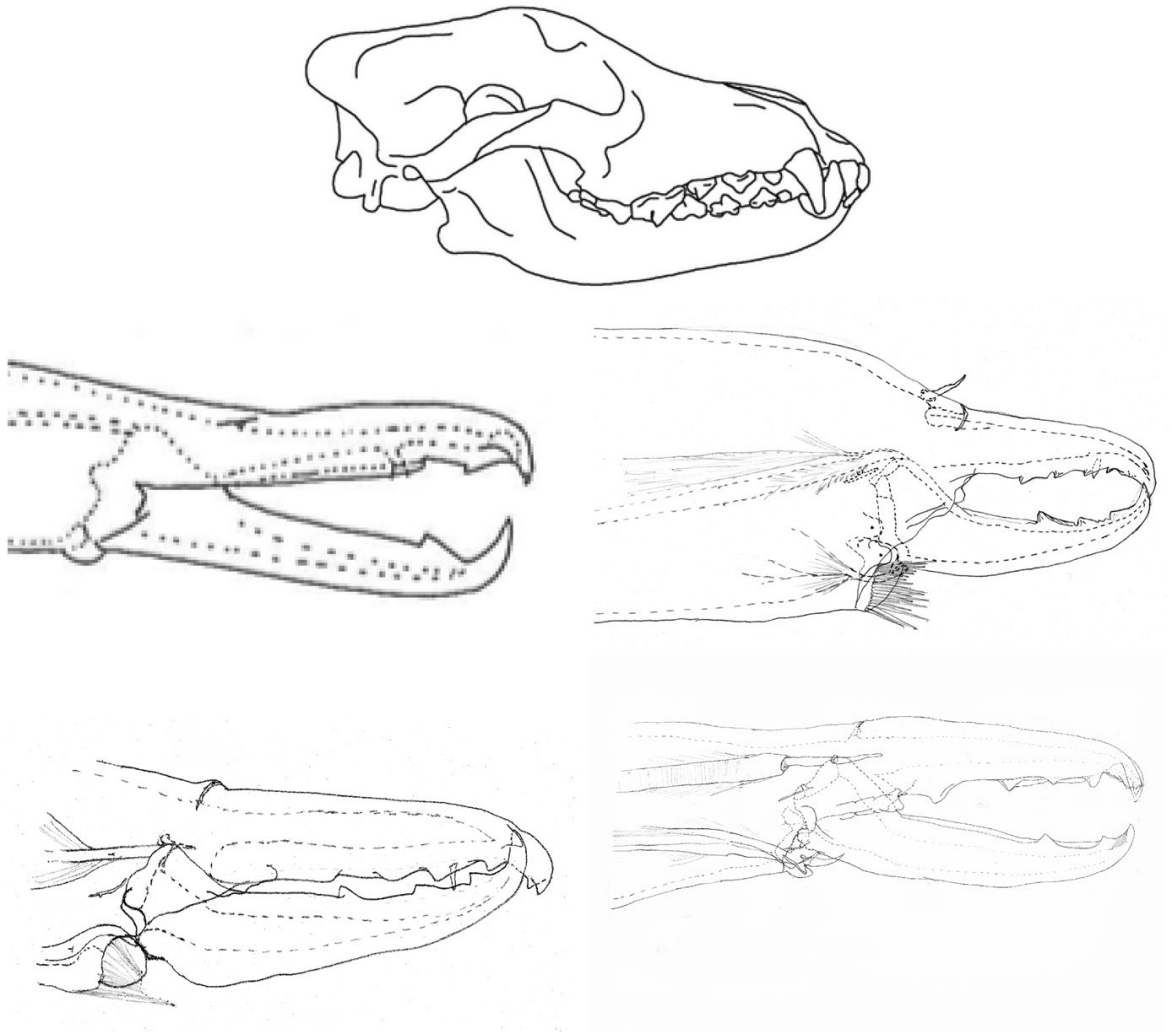
General and specialised mesostigmatid micro-arthropod feeding habit predators have gracile, dolichocephalic bladed chelae. *Upper* Dog skull from Veitschegger et al. (2018) under Creative Commons Attribution 4.0 International (CC BY 4.0). Note masticatory habit determines the morphology of the craniofacial skeleton—Roberts (1979). Velocity ratio (Radinsky 1981) estimated from jaw length moment arm (JL)/temporalis moment arm (MAT) in diagram: $\text {Canid} = 0.220$ *Middle and Lower* Parasitid chelae at various scales. Levator and depressor tendons marked. *Middle* Left to Right, at different scales: *Polyaspis (Polyaspis) flechtmanni,* DN from Hirschmann and Kemnitzer 1989 under Creative Commons-BY-NC-ND which permits unrestricted non-commercial use, distribution, and reproduction in any medium, provided the original author and source are credited; *Parasitus coleoptratorum* DN ; *Lower* Left to Right at different scales: *Parasitus lunaris* DN; *Veigaia nemorensis* female

Whilst the exact velocity ratios in these vertebrate jaws do not numerically match those of mesostigmatids (Figs. 29, 30, 31 and Table 3), the ranking that the blade-like canid design is the lowest does. Even though the vertebrate jaw is a much more complicated lever system (superficial and deep masseter moment arms as well as carnassial bite moment arms have been ignored here) a degree of consilience is clear.

Extending this, what mesostigmatid mite chelal moveable digits might match mustelid jaws (like badgers, weasels, stoats, pine martens, etc.; Radinsky 1981)? What mesostigmatid chelae might look like the mandible of bears (ursids)? What mesostigmatid chelal designs match those of civets and genets (i.e., viverrids, Ferretti 2007)? Is there any relationship of mesostigmatid chelicerae to the armaments of their prey? Could there be a mesostigmatid mite like the deep-bodied herbivorous fish *Myleus* specialised for biting pieces from plants; Alexander (1964)? Or even, could there be a mesostigmatid equivalent of the large and medium size pandas which specialise on chewing fibrous plant material (Van Valkenburgh 2007) just waiting out there to be discovered by an acarologist? Categorising mesostigmatid chelicerae/gnathosomas using other mammalian jaw/skull descriptors like robustness, gracility, dolichocephalic, brachycephalic etc (Curth et al. 2017) is left to follow-up work.

Finally, turning to comparative dentition. Which mite chelicerae have molariform chelae like the claws of xanthid crustaceans—Zipser and Vermeij (1978), with teeth adapted for grasping rather than slicing flesh? Are there instances of ‘overbite’, ‘over-jet’, malalignment etc (like vertebrates—Smith et al. 1978) in the occlusion of mesostigmatid cheliceral digit dentition? Patterns of dentition and occlusion is important in brachyuran chelae (Schenk and Wainwright 2001). Can Walter and Ikonen (1989)’s mesostigmatid dentition types 1–4 (their Fig. 2) be clearly mapped to the standard shear/compressive classification used in decapod crustaceans (Brown et al. 1979) or to those vertebrates (with velocity ratio values calculated for each tooth)? Do teeth offset between the moveable and fixed digit produce couples to rotate food (see Gans 1974)? To what extent could each chelal digit be taken as an independent yet integrated unit like in the lower versus the upper jaw of dogs (Curth et al. 2017)? Would plotting each species in a space of the vector of scored velocity ratio values it shows per tooth explain the variety of dentition designs for nematophages (Karg 1983; Walter and Ikonen 1989)? Are there special linear regions of similar crushing functions across species? Note that not all ‘claws’ have the potential for being ‘fast claws’ (Brown et al. 1979)—even a crocodile tosses its prey onto its back teeth to chew. Could this set of velocity ratio values be mapped to the ideal form (Evans and Sanson 2003) of the dentition for slicing versus mashing at specified locations along the chela? After all Buryn and Brandl (1992) did find that worm-like diets were associated with chelicerae characterised by a large number of teeth along the digitus fixus and digitus mobilis. Could their function in preventing translational, rotational and skew movements of the prey be determined (Brown et al. 1979)? Are diastemas effectively present?

There is scope for much more work simply morphologically matching dentition designs across animals in a mechanical context before deploying sophisticated statistical morphometrics on mites and their phylogenetic relationships as done in other groups (e.g., Nicola et al. 2003). Nature first sees teeth within levered jaws and only thereafter abstract statistical mathematics!

## Look to the future

As well as the many specific suggestions for acarological follow-up detailed above to demonstrate the adaptive significance of cheliceral features (not just to assume it—Futuyma 1979), different sorts of questions could be answered and very different analyses could be done.

### General points

‘Historical analysis’ (Lauder 1982) is concerned with the evolutionary transformation of intrinsic organisational features and not with the relationship between form and the (extrinsic) environment. A crucial element in analysing structural transformation is the phylogenetic reconstruction of nested sets of homologies which indicate the historical sequence in which new morphological features were acquired in a lineage. Other taxa such as palpigrades who also show combinations of plesiomorphic and derived morphological traits have already been shown in this way to be closely related to parasitiform Acari (and solifugids); Ballasteros (2019). A phylogenetic approach to the analysis of mesostigmatid chelal design would reveal the historical pattern by which any particular combination of structural features was constructed. This could be as illuminating just as the many investigations into the evolution of bird beaks (e.g., Bock 1970) has shown. Have mechanical adjustments been made in mites as in mammals (Crompton 1963)? Is there phylogenetic evidence in mites for a similar process of moveable digit growth over evolutionary time much as that for the coranoid process in synapsid jaws (DeMar and Barghusen 1973)? Is there anything special mechanically in how ologamasid mouthparts are designed given their early derivative status? Ologamasids are rare and low in diversity in northern areas arising from the Laurasian ‘supercontinent’ but are a dominant groups of predatory mites in southern areas arising from the Gondwanan ‘supercontinent’ (including the Antarctic Peninsula). Bar-coding and Next Generation Sequencing (Navajas and Fenton 2000; Klompen 2010; Okassa et al. 2012) offer the ability to construct unbiased trees of identity by descent on which biomechanical characteristics of species can be overlain. What might they reveal for mesostigmatids?

**Table 13:** Relative measures for full analysis data set (= training + test data sets)

Species	CL/IL $\dag$	MDL/IL $\ddag$	MDL/CL *	F2AV/IL**
*Alliphis halleri*	30.6%	8.0%	26.3%	0.88
*Alliphis siculus*	29.9%	7.9%	26.4%	6.89
*Amblyseius okanagensis*	32.4%	8.4%	26.0%	1.19
*Ameroseius* sp.	24.5%	5.7%	23.0%	0.76
*Androlaelaps casalis*	28.5%	6.0%	21.2%	0.99
*Arctoseius brevicheles*	36.4%	6.2%	17.0%	1.45
*Arctoseius certratus*	37.5%	9.5%	25.3%	1.26
*Arctoseius minutus*	33.0%	7.2%	21.9%	0.89
*Arctoseius venustulus*	35.4%	8.2%	23.1%	0.89
*Blattisocius keegani*	24.5%	5.6%	22.8%	1.19
*Cheiroseius borealis*	43.1%	10.0%	23.2%	0.97
*Dendrolaelaps foveolatus*	32.0%	8.3%	25.9%	1.43
*Eugamasus berlesei*	32.7%	9.8%	30.1%	2.99
*Eugamasus cavernicola*	35.7%	10.4%	29.1%	2.66
*Eviphis ostrinus*	61.2%	7.5%	12.2%	0.92
*Geholaspis longispinosus*	37.8%	9.3%	24.5%	1.91
*Geholaspis* sp.	48.2%	24.2%	50.3%	3.17
*Glyphtholaspis confusa*	38.1%	9.5%	24.9%	4.09
*Hypoaspis aculeifer*	43.4%	13.2%	30.5%	1.95
*Hypoaspis angustiscutata*	43.6%	13.4%	30.8%	2.09
*Iphidozercon gibbus*	44.6%	6.7%	15.1%	1.07
*Leioseius bicolor*	36.0%	9.4%	26.1%	1.08
*Macrocheles montanus*	35.8%	10.1%	28.1%	3.21
*Pachylaelaps furcifer*	34.9%	9.7%	27.7%	1.17
*Pachylaelaps leauchlii*	36.5%	10.3%	28.1%	1.32
*Pachyseius humeralis*	31.3%	9.0%	28.6%	0.84
*Parasitus beta*	29.1%	8.1%	27.9%	1.26
*Parasitus coleoptratorum* DN	40.0%	11.5%	28.8%	3.72
*Parasitus fucorum* DN	33.4%	9.0%	26.9%	2.93
*Parasitus lunaris* DN	57.3%	19.2%	33.5%	2.08
*Parazercon radiatus*	40.5%	9.1%	22.5%	1.77
*Pergamasus cornutus*	39.6%	14.5%	36.7%	1.88
*Pergamasus crassipes*	37.6%	14.7%	39.0%	3.01
*Pergamasus digitulus*	39.5%	14.3%	36.2%	1.56
*Pergamasus mirabilis*	54.2%	20.6%	38.0%	3.43
*Pergamasus misellus*	36.9%	13.5%	36.6%	1.43
*Pergamasus oxygynelloides*	41.8%	17.3%	41.3%	1.78
*Pergamasus quisquillarum*	44.7%	16.3%	36.5%	4.18
*Pergamasus runcatellus*	41.3%	16.2%	39.2%	2.74
*Pergamasus runciger*	40.1%	15.3%	38.2%	2.57
*Pergamasus septentrionalis*	45.0%	16.0%	35.5%	5.60
*Pergamasus* sp.	41.6%	16.1%	38.7%	2.38
*Pergamasus suecicus*	45.8%	17.0%	37.1%	1.67
*Polyaspis* n.sp. DN	43.3%	15.4%	35.6%	1.11
*Porrhostaspis lunulata*	38.8%	11.9%	30.6%	1.52
*Proctolaelaps pygmaeus*	31.9%	7.1%	22.1%	1.41
*Rhodacarellus epigynalis*	42.2%	14.2%	33.7%	3.11
*Rhodacarellus sileciacus*	37.6%	11.0%	29.3%	1.44
*Rhodacarus agrestis*	53.2%	21.2%	39.8%	3.17
*Rhodacarus strenzkei*	55.9%	22.8%	40.8%	5.76
*Trachytes aegrota*	45.5%	4.4%	9.7%	0.51
*Typhlodromus setubali*	34.4%	7.8%	22.8%	1.30
*Urodiaspis tecta*	39.8%	3.0%	7.6%	0.47
*Uropoda orbicularis* DN	39.9%	3.4%	8.6%	1.09
*Veigaia cerva*	98.3%	37.4%	38.1%	3.65
*Veigaia decurtata*	75.6%	27.9%	36.9%	1.19
*Veigaia exigua*	75.1%	29.1%	38.7%	1.52
*Veigaia nemorensis* (new)	70.1%	22.5%	32.1%	2.69
*Veigaia nemorensis* (old)	63.5%	23.0%	36.1%	3.17
*Zercon peliatus*	34.5%	9.0%	26.1%	1.13

All female unless otherwise stated. $\dag$Relative reach. $\ddag$Relative gape. *Degree of which gape dominates the cheliceral reach. **Relative crunch force. Averages across all species: IL $676.0\,\mu \text {m}$; CL $285.4\,\mu \text {m}$; MDL. $87.4\, \mu \text {m}$; F2AV 1601.4; CL/IL 42.3%; MDL/IL 12.7%; MDL/CL 29.3%; F2AV/IL 2.09

**Table 14:** Heuristic conclusion for full analysis data set (= training + test data sets)—‘crushing designed’ chelal worm-like prey feeding habit (velocity ratio threshold $> 0.276$)

Gamasine Family	Species	Mite is...	Prey/Food is at a...	Which is relatively ... body $\dag$	Prey or Food relatively...	Requiring a... **	Chela takes ...	In...$\ddag$	With oral area being...*	Suggested animal analogue
Ameroseiidae	*Ameroseius* sp.	Pygmy	Short range	Close to	Wiggler/Soft	Powerful grip	Small food	Little chunks	Tiny mouthfuls	Kitten, Mole, Shrew, Small pig
Ascidae	*Arctoseius brevicheles*	Pygmy	Short range	Close to	Wiggler/Soft	Powerful grip	Small food	Little chunks	Tiny mouthfuls	Kitten, Mole, Shrew, Small pig
Ascidae	*Arctoseius certratus*	Pygmy	Short range	Close to	Wiggler/Soft	Powerful grip	Small food	Little chunks	Tiny mouthfuls	Kitten, Mole, Shrew, Small pig
Ascidae	*Arctoseius minutus*	Pygmy	Short range	Close to	Wiggler/Soft	Powerful grip	Small food	Little chunks	Tiny mouthfuls	Kitten, Mole, Shrew, Small pig
Ascidae	*Arctoseius venustulus*	Pygmy	Short range	Close to	Wiggler/Soft	Powerful grip	Small food	Little chunks	Tiny mouthfuls	Kitten, Mole, Shrew, Small pig
Ascidae	*Iphidozercon gibbus*	Pygmy	Short range	Away from	Wiggler/Soft	Powerful grip	Small food	Little chunks	Tiny mouthfuls	Carrion vulture
Ascidae	*Leioseius bicolor*	Pygmy	Short range	Close to	Wiggler/Soft	Powerful grip	Small food	Little chunks	Tiny mouthfuls	Kitten, Mole, Shrew, Small pig
Ascidae	*Proctolaelaps pygmaeus*	Pygmy	Short range	Close to	Wiggler/Soft	Powerful grip	Small food	Little chunks	Tiny mouthfuls	Kitten, Mole, Shrew, Small pig
Blattisociidae	*Blattisocius keegani*	Pygmy	Short range	Close to	Wiggler/Soft	Powerful grip	Small food	Little chunks	Tiny mouthfuls	Kitten, Mole, Shrew, Small pig
Digamasellidae	*Dendrolaelaps foveolatus*	Pygmy	Short range	Close to	Wiggler/Soft	Powerful grip	Small food	Little chunks	Tiny mouthfuls	Kitten, Mole, Shrew, Small pig
Eviphididae	*Alliphis halleri*	Pygmy	Short range	Close to	Wiggler/Soft	Powerful grip	Small food	Little chunks	Tiny mouthfuls	Kitten, Mole, Shrew, Small pig
Eviphididae	*Alliphis siculus*	Pygmy	Short range	Close to	Hard/Struggles	Feeble effort	Small food	Little chunks	Tiny mouthfuls	Mouse
Laelapidae	*Androlaelaps casalis*	Pygmy	Short range	Close to	Wiggler/Soft	Powerful grip	Small food	Little chunks	Tiny mouthfuls	Kitten, Mole, Shrew, Small pig
Macrochelidae	*Geholaspis longispinosus*	Giant	Long range	Close to	Hard/Struggles	Powerful grip	Big prey	Little chunks	Tiny mouthfuls	Tyrannosaur
Macrochelidae	*Glyphtholaspis confusa*	Giant	Long range	Close to	Hard/Struggles	Feeble effort	Big prey	Little chunks	Tiny mouthfuls	Hyena, ’Alien’
Macrochelidae	*Macrocheles montanus*	Giant	Long range	Close to	Hard/Struggles	Feeble effort	Big prey	Little chunks	Tiny mouthfuls	Hyena, ’Alien’
Parasitidae	*Eugamasus berlesei*	Giant	Long range	Close to	Hard/Struggles	Feeble effort	Big prey	Little chunks	Well stuffed	Greedy Hyena, Greedy ’Alien’
Parasitidae	*Eugamasus cavernicola*	Giant	Long range	Close to	Hard/Struggles	Feeble effort	Big prey	Little chunks	Tiny mouthfuls	Hyena, ’Alien’
Parasitidae	*Parasitus beta*	Pygmy	Short range	Close to	Wiggler/Soft	Powerful grip	Small food	Little chunks	Tiny mouthfuls	Kitten, Mole, Shrew, Small pig
Parasitidae	*Parasitus coleoptratorum* DN	Giant	Long range	Close to	Hard/Struggles	Feeble effort	Big prey	Little chunks	Tiny mouthfuls	Hyena, ’Alien’
Parasitidae	*Parasitus fucorum* DN	Giant	Long range	Close to	Hard/Struggles	Feeble effort	Big prey	Little chunks	Tiny mouthfuls	Hyena, ’Alien’
Parasitidae	*Pergamasus digitulus*	Pygmy	Short range	Close to	Wiggler/Soft	Powerful grip	Small food	Major grab	Well stuffed	Big cat
Parasitidae	*Pergamasus runcatellus*	Giant	Long range	Close to	Hard/Struggles	Feeble effort	Big prey	Major grab	Well stuffed	Greedy Tyrannosaur after flesh
Parasitidae	*Pergamasus septentrionalis*	Giant	Long range	Away from	Hard/Struggles	Feeble effort	Big prey	Major grab	Well stuffed	Pig, Wild boar
Phytoseiidae	*Amblyseius okanagensis*	Pygmy	Short range	Close to	Wiggler/Soft	Powerful grip	Small food	Little chunks	Tiny mouthfuls	Kitten, Mole, Shrew, Small pig
Phytoseiidae	*Typhlodromus setubali*	Pygmy	Short range	Close to	Wiggler/Soft	Powerful grip	Small food	Little chunks	Tiny mouthfuls	Kitten, Mole, Shrew, Small pig
Rhodacaridae	*Rhodacarellus epigynalis*	Pygmy	Short range	Close to	Wiggler/Soft	Feeble effort	Small food	Major grab	Well stuffed	Rat
Rhodacaridae	*Rhodacarellus sileciacus*	Pygmy	Short range	Close to	Wiggler/Soft	Powerful grip	Small food	Little chunks	Well stuffed	Small cat
Rhodacaridae	*Rhodacarus agrestis*	Pygmy	Short range	Away from	Wiggler/Soft	Feeble effort	Big prey	Major grab	Well stuffed	Carrion vulture after flesh
Rhodacaridae	*Rhodacarus strenzkei*	Pygmy	Long range	Away from	Hard/Struggles	Feeble effort	Big prey	Major grab	Well stuffed	Pygmy boar
Zerconidae	*Parazercon radiatus*	Pygmy	Short range	Close to	Wiggler/Soft	Powerful grip	Small food	Little chunks	Tiny mouthfuls	Kitten, Mole, Shrew, Small pig
Zerconidae	*Zercon peliatus*	Pygmy	Short range	Close to	Wiggler/Soft	Powerful grip	Small food	Little chunks	Tiny mouthfuls	Kitten, Mole, Shrew, Small pig
-	*Urodiaspis tecta*	Giant	Long range	Close to	Wiggler/Soft	Powerful grip	Small food	Little chunks	Tiny mouthfuls	Dainty Tyrannosaur
-	*Uropoda orbicularis* DN	Giant	Short range	Close to	Wiggler/Soft	Powerful grip	Small food	Little chunks	Tiny mouthfuls	Tapir

All female unless otherwise stated. Sorted by family. All have gamasine body form bar the four uropodoids. Annotation as Table 13 so data order of columns is IL, CL, CL/IL $\dag$, F2AV, F2AV/IL**, MDL, MDL/IL $\ddag$, MDL/CL*. Note suggested vertebrate mappings are analogues not strict homologues. ’Alien’ is the fictional xenomorph or *Internecivus raptus* designed by H R Giger

**Table 15:** Heuristic conclusion for full analysis data set (= training + test data sets)—‘cutting designed’ chelal micro-arthropod feeding habit (velocity ratio threshold $< 0.276$)

Gamasine Family	Species	Mite. is.	Prey/Food is at a.	Which is relatively ... body $\dag$	Prey or Food relatively.	Requiring a... **	Chela takes .	In...$\ddag$	With oral area being...*	Suggested animal analogue
Ascidae	*Cheiroseius borealis*	Pygmy	Short range	Away from	Wiggler/Soft	Powerful grip	Small food	Little chunks	Tiny mouthfuls	Carrion shark
Eviphididae	*Eviphis ostrinus*	Pygmy	Long range	Away from	Wiggler/Soft	Powerful grip	Small food	Little chunks	Tiny mouthfuls	Small Ichthyosaur, Small plesiosaur
Laelapidae	*Hypoaspis aculeifer*	Giant	Long range	Away from	Wiggler/Soft	Powerful grip	Big prey	Major grab	Well stuffed	$\male$ lion, Small tiger, Crocodile
Laelapidae	*Hypoaspis angustiscutata*	Giant	Long range	Away from	Hard/Struggles	Powerful grip	Big prey	Major grab	Well stuffed	Large crocodile
Macrochelidae	*Geholaspis* sp.	Giant	Long range	Away from	Hard/Struggles	Feeble effort	Big prey	Major grab	Well stuffed	Tiger, $\female$ lion
Pachylaelapidae	*Pachylaelaps furcifer*	Giant	Long range	Close to	Wiggler/Soft	Powerful grip	Big prey	Little chunks	Tiny mouthfuls	Dog
Pachylaelapidae	*Pachylaelaps leauchlii*	Giant	Long range	Close to	Wiggler/Soft	Powerful grip	Big prey	Little chunks	Tiny mouthfuls	Dog
Pachylaelapidae	*Pachyseius humeralis*	Pygmy	Short range	Close to	Wiggler/Soft	Powerful grip	Small food	Little chunks	Tiny mouthfuls	Small shark
Parasitidae	*Parasitus lunaris* DN	Pygmy	Long range	Away from	Wiggler/Soft	Powerful grip	Big prey	Major grab	Well stuffed	$\male$ lion, Small tiger, Small crocodile
Parasitidae	*Pergamasus cornutus*	Pygmy	Short range	Close to	Wiggler/Soft	Powerful grip	Big prey	Major grab	Well stuffed	Orca
Parasitidae	*Pergamasus crassipes*	Giant	Long range	Close to	Hard/Struggles	Feeble effort	Big prey	Major grab	Well stuffed	Large shark
Parasitidae	*Pergamasus mirabilis*	Giant	Long range	Away from	Hard/Struggles	Feeble effort	Big prey	Major grab	Well stuffed	Tiger, $\female$ lion
Parasitidae	*Pergamasus misellus*	Pygmy	Short range	Close to	Wiggler/Soft	Powerful grip	Small food	Major grab	Well stuffed	Dolphin
Parasitidae	*Pergamasus oxygynelloides*	Pygmy	Short range	Close to	Wiggler/Soft	Powerful grip	Big prey	Major grab	Well stuffed	Orca
Parasitidae	*Pergamasus quisquillarum*	Giant	Long range	Away from	Hard/Struggles	Feeble effort	Big prey	Major grab	Well stuffed	Tiger, $\female$ lion
Parasitidae	*Pergamasus runciger*	Giant	Long range	Close to	Hard/Struggles	Feeble effort	Big prey	Major grab	Well stuffed	Large shark
Parasitidae	*Pergamasus* sp.	Pygmy	Short range	Close to	Wiggler/Soft	Feeble effort	Big prey	Major grab	Well stuffed	Shark
Parasitidae	*Pergamasus suecicus*	Pygmy	Short range	Away from	Wiggler/Soft	Powerful grip	Small food	Major grab	Well stuffed	Greedy carrion shark after flesh
Parasitidae	*Porrhostaspis lunulata*	Giant	Long range	Close to	Wiggler/Soft	Powerful grip	Big prey	Little chunks	Well stuffed	Hunting dog, Leopard
Veigaiidae	*Veigaia cerva*	Giant	Long range	Away from	Hard/Struggles	Feeble effort	Big prey	Major grab	Well stuffed	Giant tiger, Big $\female$ lion, Large crocodile
Veigaiidae	*Veigaia decurtata*	Pygmy	Long range	Away from	Wiggler/Soft	Powerful grip	Big prey	Major grab	Well stuffed	$\male$ lion, Small tiger, Small crocodile
Veigaiidae	*Veigaia exigua*	Pygmy	Long range	Away from	Wiggler/Soft	Powerful grip	Big prey	Major grab	Well stuffed	$\male$ lion, Small tiger, Small crocodile
Veigaiidae	*Veigaia nemorensis* (new)	Giant	Long range	Away from	Hard/Struggles	Feeble effort	Big prey	Major grab	Well stuffed	Tiger, $\female$ lion
Veigaiidae	*Veigaia nemorensis* (old)	Pygmy	Long range	Away from	Hard/Struggles	Feeble effort	Big prey	Major grab	Well stuffed	Small tiger
−	*Polyaspis* n.sp. DN	Giant	Long range	Away from	Wiggler/Soft	Powerful grip	Big prey	Major grab	Well stuffed	$\female$ lion, Big tiger, Crocodile
–	*Trachytes aegrota*	Giant	Long range	Away from	Wiggler/Soft	Powerful grip	Small food	Little chunks	Tiny mouthfuls	Large Ichthyosaur, Plesiosaur

All female unless otherwise stated. Sorted by family. All have gamasine body form bar the four uropodoids. Annotation as Table 13 so data order of columns is IL, CL, CL/IL $\dag$, F2AV, F2AV/IL**, MDL, MDL/IL $\ddag$, MDL/CL *. Note suggested vertebrate mappings are analogues not strict homologues. ’Alien’ is the fictional xenomorph or *Internecivus raptus* designed by H R Giger

Heuristics cover any approach to problem solving or self-discovery that employs a practical method that is not guaranteed to be optimal, perfect or rational, but which is nevertheless sufficient for reaching an immediate, short-term goal. Dividing the range of the measurements (IL, CL, MDL, and F2AV) around each mean can produce a ‘high-low’ cut that can be described in approximate verbal terms. Similar can be done for relative reach (CL/IL), relative gape (MDL/IL), MDL/CL and F2AV/IL (Table 13). Allocating colloquial phrases to the measures above and below their mean enables one to pose an analogue of each mite as a matching vertebrate (to aid interpretation of a mite’s role not to yield an exact homologue)—Tables 14, 15. Such predatory interim roles need further investigation, refinement and confirmation but do show a degree of commonality with mesostigmatid taxonomic hierarchy. Buryn and Brandl (1992)’s approach with size adjusted residuals did pick up the signal of: whether the prey was close to, or relatively away form the predator (CL/IL); whether there was a little chunk or, a major grab of food (MDL/IL); and, perhaps if the oral area was well stuffed to overflowing or just full of tiny mouthfuls (MDL/CL). However, by taking a strict morphometric approach which partialed out size and ignored any mechanics, they missed: the importance of small mites (IL) and the commonality of large mesostigmatids with solifugids; the importance of prey attack radius or range (CL); the importance of prey/food toughness (F2AV); the importance of chelal grip (relative crunch power F2AV/IL); and, the gape (MDL) needed for the actual size of prey consumed. They unfortunately missed too the key insight of a strength-speed contrast that velocity ratio offers. All of these biological considerations affect the predatory role that a mite (or indeed a vertebrate) might play in an ecosystem.

Increased cheliceral chelal crunch force (F2AV) against prey tissues is resisted by any evolved compensating reciprocal adaptations in the prey. Beetles that attack oribatid mites for instance have certain adaptations (Jałoszyński 2018), and oribatid mites have developed different body designs amenable to resisting crushing (Schmelzle and Blüthgen 2019). It would intriguing to look for such reciprocal adjustments in the popular prey of specialist free-living mesostigmatids highlighted herein this review.

Are there acarine equivalents of crustacean ‘pectinate claws’ (Tshudy and Sorhannus 2000)? These almost impossibly long slender-fingered chelae armed with pectinate (comblike) denticles found in extant and fossil decapods. They have evolved independently in multiple lineages—so there is some sort of consistent trophic selection for them in the wild. Could *Rhodacaroides aegyptiacus* n.sp. described in Willmann (1959) be a mite with chelicerae like this?

### Regarding parasitines

**Fig. 32:**
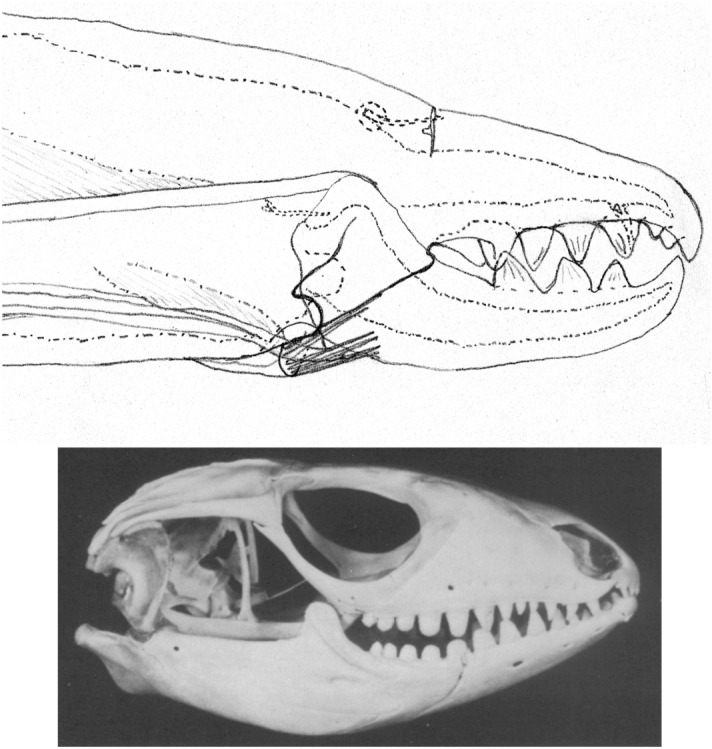
Some mesostigmatid cheliceral chelae can be specialised for hanging on to things hard. *Upper* *Parasitus fucorum* DN. Levator and depressor tendons marked with pennate muscles as dots and dashes. Dot as condyle position. *Lower* Molluscivorous *Tubinaphis* sp. snail crushing lizard ex Dalrymple (1979a) © Journal of Herpetology with permission

Hyatt (1980) includes in his monograph a tabulation of the principal habitat preferences of parasitines, ranging from association with Bumblebees through to a habitat of Fungi (including mushroom beds). This review (see Tables 11, 12 from Hyatt (1980)’s drawings) characterises the chelal velocity ratio for 35 out of 36 of the species scored in his Table 1 p. 241. Six out of 35 (17%) of these would be considered to have a crushing style feeding habit (i.e., $p(wormlike feeder)\ge 0.5$). In declining velocity ratio values these are: *Eugamasus crassitarsis*, *Parasitus beta*, *Parasitellus ignotus*, *Parasitellus fucorum*, *Eugamasus berlesei*, *Eugamasus cavernicola*. Note that two of the eugamasids appear to be designed inconsistently (marked by * in Table). A question arises—do any of the habitats have too many or too few representatives of the worm-like feeding habit (given that for at least the *Parasitus* spp. series the slicing/cutting micro-arthropod killing design persists over all body scales—Fig. 27)? This question can be checked by a simple *z*-test of the proportion of species having a worm-like feeder design being different to 0.17 amongst those parasitines which occur in that habitat (Table 16). Association with Bumblebees (and to some extent other Hymenoptera) exhibits too many worm-like feeding crushing design species. The Seashore and Houseplants/greenhouse soil (and perhaps Manure, dung, sewage) have generally too few worm-like feeding design species than expected. This is at an extreme for Association with corpses/sexton-beetles or in the Fungal habitat. One interpretation of this is that hymenopteran associates in general need crushing design chelae to hang on to insect body hairs during phoresy (Fig. 32). Another conclusion is that in comparison to habitats in general, temporary accumulations of ‘debris’ (i.e., seashore; manure, dung, sewage; corpses) and anywhere rich in fungi (mushroom beds; houseplants/greenhouse soil) are likely to have many more micro-arthropods infesting them than have opportunities for the few worm-like feeding design specialist parasitines. A conclusion like this is found in Seeman and Nahrung (2018)—phoretic or parasitic mites are bigger than free-living mites. So if big mites tend to be crusher-style feeders, then you would expect more mites with crushing adapted chelicerae in phoretic mites (albeit that large adductive force F2 might also be an adaptation for holding onto host hairs during transport). That both phoretic and parasitic mesostigmatids are big is intriguing, i.e., why are there few tiny parasitic Mesostigmata—tiny parasites certainly occur in the Prostigmata? More work is needed to understand evolutionary trade-offs in mites.

**Table 16:** Variation of habitat in terms of over and under-representation of worm-like feeding designs for parasitines based upon cheliceral chela velocity ratios estimated from drawings in Hyatt (1980) versus null of $6/35 = 17\%$ (two-sided *z*-test, no multiple testing correction)

Parasitine habitat	Frequency of worm-like crushing cheliceral design	Over or under represented	*p*-value
Bumblebees	3/8	Over	$\le 0.05$
Other Hymenoptera	1/3	Over	ns $\dag$
Scarabaeid or ground beetles	1/7	under	ns
Other insects	1/7	under	ns
Mammals or their nests	3/18	under	ns
Birds or their nests	1/10	under	ns
Seashore	1/14	under	$<0.1$
Arable grassland	2/12	under	ns
Leaf litter, mosses	4/17	Over	ns
Houseplant/greenhouse soil	0/4	under	$< 0.1$
Hay, straw, grain	1/7	under	ns
Compost (rotting vegetation)	2/12	under	ns
Manure, dung, sewage	1/11	under	ns $\dag$
Corpses/sexton-beetles	0/7	under	$<0.06$
Fungi (including mushroom beds)	0/7	under	$<0.06$

$\dag$Almost significant at $p=0.1$ level

### Regarding uropodines and idiosomal sclerotisation

The suborder Uropodina is a vast 300 genus-group recently listed in detail by Halliday (2015). What might overlaying estimates of: size, reach, relative reach, aspect ratio, gape, relative gape, gape/reach, velocity ratio, chelal crunch force, relative crunch force etc (cf. Tables 3, 10, and 13); reveal about this poorly known group of predators and fungivores? Can vertebrate animal analogues (like in Tables 14 and 15) be suggested for all the type-species listed by Halliday (2015)? How probable is their trophic assignation from the velocity ratio model (cf. Table 8)? Could any predicted as ‘worm-like’ prey feeders be confirmed as consuming nematodes using specific molecular markers for their prey (see Heidemann et al. 2014)? Soil nematodes are very small (0.3–5.0 mm long as adults)—would the gape of confirmed nematode-feeding uropodoids be suitable to grasp such (thus examining hypothesis *(iii)* more)? Free-living uropodoids seemingly can grasp food morsels in the size range of small pollen grains (cf. Table 3; pollen size$= 15\text {-}200\,\mu \text {m}$). Large fungal spores can be over than 20 microns, while the tiniest spores are only 4–5 microns across and hyphae have an average diameter of $4\text {-}6\,\mu \text {m}$. So could the gape of high aspect ratio uropodoids, predicted by their velocity ratio values to be possible saprophages, in turn be related to the size of fungal spores that they eat or the typical mycelium/hyphal widths of fungi that they might browse (again probing hypothesis *(iii)* more)? Algae have a great range of shapes and sizes, from spherical cells with $0.5\,\mu \text {m}$ diameter to 60 m long multicellular thalli. Walter (1987) does not list mesostigmatids in his experimental tests of mycophagous microarthropods, but certainly the gape of uropodoids would allow the gripping (if not consumption) of some soil algal material. More biological observations are needed.

If the evolutionary origin of body sclerotisation in the uropodoids was like that of turtle armour (Yong 2016; Black 2020; Schoch and Sues 2020), where evolutionarily the fossorial habit engendered first ventral strengthening between the legs for facilitating burrowing (through increased locomotory stability), before a secondary dorsal carapace was developed, then a key ancestral mechanical innovation in mesostigmatids could have been the ventral sternal shield and ‘coxal’ strengthening (as the worm-like ancestral body shrank under segmental consolidation). Given this: the position of the coxal gland openings (Bowman 1984) which debouch excess fluid ventrally; the ventral deuterosternal groove on the infracapitulum with the tritosternum (Wernz and Krantz 1976); the gnathosomal groove; and, the gnathotectum etc then all would facilitate liquid recycling needed for predation on watery prey (Bowman 2014, 2017b, 2019) when burrowing, delving into and excavating soil pores for food or shelter. Coxae in arachnids are discussed in Van der Hammen (1977a) where he points out that some of the most primitive arachnid members have been found in the deeper layers of soil. A more recent discussion can be found in Klompen et al. (2015). Are there primitive eudaphic mesostigmatids with essentially little dorsal sclerotisation? Could then the subsequent evolution of dorsal strengthening be sclerotisation to protect and stabilise the internal organs of such mesostigmatids from the stresses of digging (and spreading muscle attachment loads arising from the increasing power requirements of predating large prey)?

### Regarding primitive relatives of mesostigmatids

The anactinotrichid Holothyrida are often pointed to as close relatives of mesostigmatids. Holothyrids have: coxal glands (Thon 1905; Alberti and Seeman 2004); chelicerae that retract into their idiosoma (Alberti et al. 2006); and, vertically orientated and acting cheliceral chelal digits (Klompen *pers.comm.*). Whether their gnathosoma is enclosed above by a gnathotectum is not clear. Their gnathosoma seemingly can be klinorhynchid or airorhynchid. The chelicerae apparently can be like gamasines with no extra basal segment, or with an extra baso-basal segment like uropodines. Allothyrids have a tritosternum, holothyrids and neothyrids do not. Allothyrids consume the body fluids of dead arthropods and dead crustaceans (Walter and Proctor 1998) being deemed scavengers ingesting fluids only (Van der Hammen 1972a). Table 17 gives some approximate estimates extracted from illustrations in various key papers on this group. With one exception, all species are predicted from their chelal velocity ratio to have the ‘slicing/slashing kill’ style of feeding. Velocity ratio values are consilient with those of predatory crab chelae, predatory solifugids, veigaiids and *Polyaspis* n.sp. The one estimated chelal crunch force value (F2AV for *Diplothyrus lecorrei*) concurs well with those of large predatory gamasines (compare Tables 17 to 3). Note this uses the baso-basal segment in this calculation. Holothyrids appear to have significant reach and gape (but their IL was not available to better judge this).

**Table 17:** Approximate estimates extracted from illustrations in key papers on Holothyrida

Taxon	Sex	DSL	BSL	BBSL	Reach (CL)	Gape (MDL)	Crunch force (F2AV)	L1	L2	Velocity ratio (VR)	HDS	HBS	HBBS	Reference
**Allothyridae**														
*Australothyrus ocellatus*	$\female$			$\ddag$						0.230				Van der Hammen (1983)
**Hollothyridae**														
*Hammenius grandjeani gressitti*	$\male$									0.275 $\dag$				Lehtinen (1995)
*Hammenius insularis*	$\male$									0.275 $\dag$				Lehtinen (1995)
*Hammenius (Leiothyrus) holthuisi*	$\male$									0.193				Van der Hammen (1983)
*Hammenius (Leiothyrus) nitidissimus*	$\female$									0.251 $\dag$				Lehtinen (1995)
*Hammenius (Thonius) longipes*	$\male$									0.195 $\dag$				Lehtinen (1995)
*Hammenius (Thonius) mendi*	$\male$									0.283				Lehtinen (1995)
*Hammenius (Thonius) montanus*	$\male$									0.227				Van der Hammen (1983)
*Haplothyrus hyatti*	$\male$									0.226				Lehtinen (1995)
*Haplothyrus* sp. BMNH	DN									0.206				Lehtinen (1995)
*Lindothyrus rubellus*	Adult									0.325				Lehtinen (1995)
**Neothyridae**														
*Diplothyrus lecorrei*	$\female$	327.2	346.0	294.4 $\ddag$	967.6	265.4	4551.8 $\ddag$	51.6	253.2	0.204	104.7	140.0	173.7 $\ddag$	Klompen (2010)
*Diplothyrus lehtineni*	$\female$					107.5		29.2	94.6	0.308				Vázquez et al. (2016)
*Diplothyrus lehtineni*	$\male$	303.0	222.7	-		168.1		45.7	158.3	0.289	100.2	134.0	-	Vázquez et al. (2016)

Abbreviations as in previous tables. Length measures in $\,\mu \text {m}$. $\ddag$ = Extra baso-basal cheliceral segment as in uropodoids. $\dag$ = Incomplete moveable digit illustrated, rough estimate. WDS, WBS, WBBS taken to = HDS, HBS, WBBS respectively

The anactinochrid Opilioacarida (Van der Hammen 1970c, 1972a) are an ancient group (Dunlop and de Oliveira Bernardi 2014) which may be even more primitive relatives of mesostigmatids. These have vertically acting chelal digits in independently acting somewhat bent downwards facing tubular chelicerae free of any covering dorsally (https://youtu.be/gOoIwun923E). Cheliceral segmentation seemingly appears gamasine-like (Klompen *pers. comm.*). There is an unfused duplex setae tritosternum-like structure ventrally—Van der Hammen 1972a; Klompen *pers. comm.*). Table 18 gives some approximate estimates extracted from illustrations in various key papers on this group. Opilioacarids are thought to be solid food eaters, consuming various things including living and recently dead arthropods (Walter and Proctor 1998). All species are predicted to have the ‘crushing kill’ style of feeding from the velocity ratio model. None of the velocity ratio values are as high as herbivores like horses; phtyophagous oribatids; or *Alliphis siculus*. Rather, they span the range of those in saprophagous astigmatids, the chelae of crushing crabs, and the cheliceral chelae of *Cilliba cassidea* and *Glyphtholaspis confusa*. Their chelal crunch force values are three to six times that of the larger free-living predatory mesostigmatids studied herein (compare Table 18 to Table 3). Their reach and gape approximate those of the predatory mesostigmatids studied herein, but their IL is expected to be bigger (if it was measured).

A follow-up study focusing upon the mechanics of cheliceral design and if this might relate to Van der Hammen (1970b)’s scheme of proposed evolutionary changes in the gnathosoma is suggested for both groups. There is still plenty for acarologists to do in discerning all of the affinities of mites and ticks (see Dunlop and Alberti 2008).

**Table 18:** Approximate estimates extracted from illustrations in key papers on Opilioacarida

Taxon	Sex	DSL	BSL	Reach (CL)	Gape (MDL)	Crunch force (F2AV)	L1	L2	Velocity ratio (VR)	HDS	HBS	Reference
*Brasilacarus cocaris*	Female	269.7	70.8	340.4	89.0	17345.4	44.2	87.9	0.503	147.3	201.8	Vázquez et al. (2015)
*Brasilacarus cocaris*	Male	229.3	61.0	290.4	77.1	7348.7	34.3	74.7	0.459	96.5	130.8	Vázquez et al. (2015)
*Brasilacarus cocaris*	Female				76.2							Vázquez et al. (2015)
*Caribeacarus armasi*	Male	198.8	85.2	284.0	77.3	10957.5	39.7	73.6	0.539	114.7	149.8	Vázquez and Klompen (2009)
*Caribeacarus armasi*	Male	204.4	91.8	296.2	80.7	11388.1	41.3	75.5	0.546	115.6	150.8	Klompen et al. (2015)
*Caribeacarus brasiliensis*	Male	227.6	201.2	428.7	90.1	11728.5	34.9	88.8	0.393	137.6	161.0	de Oliveira Barnardi et al. (2013)
*Neocarus bajacalifornicus chamelaensis*	Male	167.7	128.8	296.5	59.6	7854.2	27.7	60.9	0.455	104.9	127.2	Vázquez and Klompen (2009)
*Neocarus belizensis*	Female								0.506			Vázquez et al. (2018)
*Neocarus belizensis*	Male								0.528			Vázquez et al. (2018)
*Neocarus caipora*	Female	381.2	256.7	637.9	125.6	34429.1	58.8	126.8	0.464	220.2	255.9	de Oliveira Barnardi et al. (2014)
*Neocarus calakmulensis*	Female	148.5	115.6	264.1	56.8	4088.5	20.7	54.7	0.379	81.3	97.4	Vázquez and Klompen (2009)
*Neocarus chactemalensis*	Female	239.8	169.3	409.1	93.3	13456.3	36.9	86.5	0.427	141.7	173.2	Vázquez and Klompen (2015)
*Neocarus comalensis*	Female				73.5		27.7	74.7	0.371			Vázquez and Klompen (2015)
*Neocarus proteus*	Larva	151.9	86.8	238.7	50.6	4259.0	21.0	50.4	0.417	80.9	96.3	de Oliveira Barnardi et al. (2013)
*Neocarus proteus*	DN	171.6	112.6	284.2	57.6	5611.8	24.5	56.5	0.434	92.9	102.8	de Oliveira Barnardi et al. (2013)
*Neocarus proteus*	TN	190.5	105.8	296.3	59.9	5046.3	20.4	59.1	0.346	96.5	112.5	de Oliveira Barnardi et al. (2013)
*Neocarus proteus*	Female	201.7	131.2	332.9	72.5	7287.2	30.7	70.8	0.433	100.7	122.3	de Oliveira Barnardi et al. (2013)
*Neocarus texanus*	Female				89.2		39.0	93.2	0.418			Vázquez and Klompen (2015)
*Neocarus veracruzensis*	Female				86.5		38.9	83.0	0.468			Vázquez and Klompen (2015)
*Neocarus veracruzensis*	Male	233.4	133.2	366.6	85.7	11060.3	35.7	81.7	0.436	123.6	159.3	Vázquez and Klompen (2009)
*Opilioacarus bajacalifornicus*	-				109.6		55.0	115.1	0.478			Vázquez and Klompen (2002)
*Opilioacarus nicaraguensis*	Male				98.0		40.7	89.5	0.455			Vázquez and Klompen (2002)
*Opilioacarus nohbecanus*	–				83.7		32.5	75.7	0.429			Vázquez and Klompen (2002)
*Opilioacarus siankaanensis*	Female								0.487			Vázquez and Klompen (2002)
*Opilioacarus siankaanensis*	Female				75.9		32.5	76.2	0.427			Vázquez and Klompen (2002)
*Opilioacarus siankaanensis*	Male				76.7		29.6	73.7	0.401			Vázquez and Klompen (2002)
*Opilioacarus texanus*	–				72.7		26.9	67.6	0.397	105.8	–	Vázquez and Klompen (2002)
*Salfacarus antisirananensis*	Male	254.8	177.5	432.3	95.6	19415.3	41.7	91.5	0.455	152.0	221.3	Vázquez and Klompen (2010)
*Salfacarus antisirananensis*	Larva	103.9	40.0	143.9	34.7	2218.0	14.2	30.8	0.461	61.8	64.7	Klompen et al. (2015)
*Salfacarus kirindiensis*	Female	259.3	185.2	444.5	104.9	11110.3	32.0	93.5	0.342	142.4	172.7	Vázquez and Klompen (2010)
*Salfacarus mahafaliensis*	Female	278.4	205.7	484.0	123.5	16471.0	41.6	109.5	0.379	170.9	201.4	Vázquez and Klompen (2010)
*Salfacarus ranobensis*	Male	226.0	162.5	388.5	91.0	15294.8	39.6	85.5	0.463	136.6	191.7	Vázquez and Klompen (2010)

Abbreviations as in previous tables. Length measures in $\,\mu \text {m}$. WDS, WBS taken to = HDS, HBS respectively

## Conclusion

Hurlbutt (1968) was right, animals “...which are markedly different in size and structure of mouthparts...show differences in feeding habits”. Buryn and Brandl (1992) were slightly unlucky in the span of morphotypes that they sampled. Using a strong morphometric focus is not necessary in ecomorphological investigations and can cloud conclusions. Mechanically, predatory mesostigmatids *do* have chelate-dentate cheliceral designs with clear purposes—even across confounding taxonomic boundaries. Although the functional groupings used herein are artificial constructs and are not necessarily pre-existing components of nature (Walter and Proctor 2013), micro-arthropod versus worm-like prey feeders are distinct respectively as prey ‘cutters’ ($\equiv$ chelae like scissors) versus prey ‘crushers/manglers’ ($\equiv$ chelae like pliers). A measurement study of mesostigmatid prey physical hardness would confirm this as has already been validated in crabs (Preston et al. 1996). Could the methods of Schmitzle and Blüthgen (2019) be useful here? These two contrasting killing-style designs match the trade-off between strength and speed.

More work is needed to see if the sizes (and uses in feeding) of mesostigmatid leg 1 and pedipalps are also correlated with their cheliceral and chelal adaptations (i.e., if evolutionary concerted or not), so that one could be sure that crushing is related to nearby food and slicing to far-away food (one interpretation of Buryn and Brandl 1992’s morphometric conclusion). Similarly, amongst non-ambush predators, cheliceral and chelal designs may vary between mesostigmatids that pursue prey (thus are likely to be diet specialists with a narrow range of food sizes and types yet being habitat generalists—i.e., Type I of Schoener 1969) versus those mesostigmatids that merely search for prey (thus are likely to be diet generalists with a wide range of food sizes and types and habitat specialists—i.e., Type II of Schoener 1969). Of course in practice mites feed on many different foodstuffs not just one type and so caution in any mechanical simulations is advised (Milne 2008).

Scale matters. Just two functional group designs do not fit all—such a result is necessary but insufficient to account for everything (Rotenberry 1980). Subtle variations in gnathosomal and chelal shape appear to be related to detailed feeding ecology in mesostigmatids. Paying attention to small organisms (Paine 1996) indicates possible ‘predator–scavenger’ mesostigmatids or perhaps ‘predator–fungivore’ mesostigmatids. Some species within these may be mesocarnivores or hypocarnivores (Van Valkenburgh 2007).

One could argue that soil and plants offer more (and smaller) microhabitats to be exploited and thus be available for occupations by only the smaller species of free-living mesostigmatids (Seeman and Nahrung 2018). Indeed for sure, a modest size pollen-feeding design variant is detected herein. At small body sizes, designs for non-predation (or at least scavenging of prey fragments) occur especially in uropodoid forms by design or perhaps opportunistically (Rotenberry 1980). Mites as a sub-class have remained small over geological time (Sidorchuk 2018). A few members of the Microgyniina are still tiny but many of the primitive Sejina and Trygynaspida are pretty big and their taxonomic outgroups: holothyrids and opilioacarids (Van der Hammen 1972a), are behemoths of the mite world. Notwithstanding this, if mesostigmatids started out very small themselves (i.e., with a mesocarnivorous weak but fairly rapid action cheliceral chela) then the results of this study herein indicate that they, back then, could not only have been predatory in habit but also, given an appropriate high velocity ratio chelal design, saprophagous consumers of particulate matter (see Walter and Proctor 1998). In that way, the results of this paper supports Walter and Proctor (2013)’s view on the acarine plesiomorphic ‘basal’ habit rather than the alternative ancient fluid feeding habit proposed by Krantz (1978).

Of course matters may be more complicated. Dunlop and Alberti (2008) gives an excellent synthesis of the different views regarding the origin of acarines. Molecular evidence of acarine relationships is an active current area (e.g., Dobson and Barker 1999; Klompen 2000; Rojas et al. 2001; Lekveishvili and Klompen 2004a; Klompen et al. 2006; Jeyaprakash and Hoy 2009; Dunlop and Selden 2009; Pepato and Klimov 2015; Estrada-Peña and de la Fuente 2018) with final agreement of exact phylogeny to come (perhaps comparative embryology might help; Van der Hammen 1972b?). However, if one poses a plesiomorphic fluid-feeding arachnid ancestor out there, then phylogenetically within the Arachnida, we should see a less parsimonious trajectory, i.e., mites going from that fluid-feeding habit to particulate feeding (perhaps through the evolution of small size?) and then ‘rediscovering’ prey fluid feeding as the body of anactinotrichid taxa increased in physical size under Cope’s rule.

As Stanley (1973) says “...as more extreme niches are occupied the amount of evolutionary displacement needed is about as easy to achieve, with a given organization, by a change in size of a given factor in either direction”. It is known from vertebrates that hypercarnivory tends to evolve along with an increase in body mass resulting in predators that regularly take prey at least half their size or larger (Van Valkenburgh 2007). Being the right size is important (Roff 1981)—a predatory (only) habit, like that of solifugids, starts for sure in mesostigmatids at a cheliceral length (reach) of $350\,\mu \text {m}$ or more, or a chelal gape of $150\,\mu \text {m}$ or more (i.e., for mites from around $500\text {-}650\,\mu \text {m}$ in size). This may represent a discontinuous ‘switch point’ much as in beetle mandible evolution (Hanley 2001; Tatsuta et al. 2004). Larger body size expands the range of potential prey sizes, reduces the risk of intraguild predation, and favours victory in interspecific encounters (Van Valkenburgh 2007).

Predatory specialisms appear to often match mite genera. Perdomo et al. (2012) was right, cheliceral form is a first quick and inexpensive filter for evaluating diet in mites. Ecologists *can* use a simple check of mesostigmatid trophic morphology based upon chelal lever arm velocity ratio to probabilistically infer their main feeding habit type from their field specimens. Then pose the detail of how the particular mite uses their mouthparts to access food in their micro-habitat and how they possess various digit teeth for different specific actions.

Irrespective of what next steps are taken, it is worth recalling as Futuyma (1979) says: “Biological thought is so permeated by the recognition that many, perhaps most, features have functions that we often forget that organisms are not perfect. In many ways they are suboptimally constructed compared with the ideal forms that an engineer might design”.

Only the acarologists’ lack of imagination restricts how cheliceral chelal velocity ratio values could be used in research. After all: “Every mite has it’s moments”!

## Footnotes

1 For the distinction between functional groups and guilds, see detail and references in Walter and Proctor (2013).
